# Inversion model for extracting chemically resolved depth profiles across liquid interfaces of various configurations from XPS data: PROPHESY

**DOI:** 10.1107/S1600577523006124

**Published:** 2023-08-23

**Authors:** Matthew Ozon, Konstantin Tumashevich, Jack J. Lin, Nønne L. Prisle

**Affiliations:** a Center for Atmospheric Research, University of Oulu, PO Box 4500, Finland; ESRF – The European Synchrotron, France

**Keywords:** X-ray photoelectron spectroscopy, depth profile, inversion algorithm, atmospheric surfaces, acquisition model

## Abstract

PROPHESY, a technique for the reconstruction of surface-depth profiles from X-ray photoelectron spectroscopy data, is introduced. The inversion methodology is based on a Bayesian framework and primal-dual convex optimization. The acquisition model is developed for several geometries representing different sample types: plane (bulk sample), cylinder (liquid microjet) and sphere (droplet). The methodology is tested and characterized with respect to simulated data as a proof of concept. Possible limitations of the method due to uncertainty in the attenuation length of the photo-emitted electron are illustrated.

## Abstract 2

The PROPHESY framework for obtaining absolute depth profiles from discrete X-ray photoelectron spectra is introduced. It is composed of (i) a model for an X-ray photoelectron spectroscopy experiment accounting for sample geometry, and (ii) an inversion model to reconstruct the concentration profile at the surface of a liquid sample. As a proof of concept, it is applied to simulated data.

## 1. Introduction

Atmospheric aerosols affect Earth’s radiative balance by absorbing and scattering solar radiation (direct aerosol climate effect) as well as by modifying cloud properties as nucleation seeds for cloud droplets (indirect aerosol climate effects) (Ramanathan *et al.*, 2001 ▸; Schulze *et al.*, 2020 ▸). Aerosols still constitute the major uncertainty in estimating the global radiative climate forcing (Arias *et al.*, 2021 ▸), hindering the scientific understanding of climate change. Part of the uncertainty is due to an incomplete understanding of how liquid droplets interact with water and other gas-phase chemicals present in the atmosphere (Heitto *et al.*, 2022 ▸). The interaction between the gas and condensed phase is mediated by the droplet surface which governs the mass transfer between phases. Droplet surfaces are chemically and physically distinct from the bulk and comprise a significant fraction of the condensed atmospheric phases, due to the high surface area to volume ratios (SA/V) of atmospheric droplets (Prisle *et al.*, 2010 ▸; Bzdek *et al.*, 2020 ▸; Prisle, 2021 ▸; Lin *et al.*, 2021 ▸). For surface-active species, the reaction rate at the droplet surface is the rate-limiting step for heterogeneous OH oxidation reactions (Huang *et al.*, 2018 ▸). Although considerable progress has been achieved in the surface characterization of aqueous systems, significant gaps remain, particularly in the unexplored transition region between the surface and bulk.

Direct measurements of particle and aqueous surfaces of atmospheric relevance have become possible with recent developments in X-ray photoelectron spectroscopy (XPS). While traditionally applied to solid state matter (Cardona & Ley, 1978 ▸), improvements in ambient-pressure measurement capabilities such as better analyzer pre-lenses, optimized differential pumping and liquid microjet technology have enabled XPS measurements on liquids, while the increased photon flux at the latest generation of synchrotron radiation facilities has allowed for measurements on dilute aqueous solutions of atmospheric relevance while targeting a wider range of atmospherically relevant elements deeper below the surface. Samples irradiated by X-rays emit photoelectrons (PEs) due to the photoelectric effect. PE count rates are recorded as a function of PE kinetic energy *K*_e_, and the location and intensities of peaks in the PE spectrum are used to determine the identity and abundance of chemically distinct species.

The attenuation of the PE signal is determined by the quantity and nature of interactions that the PEs undergo in the sample. These interactions can be classified as either elastic or inelastic. The inelastic scattering in water is characterized by the inelastic cross-section (Emfietzoglou, 2003 ▸; Emfietzoglou *et al.*, 2013 ▸) and is related to the inelastic mean free path (IMFP) (Thürmer *et al.*, 2013 ▸; Nguyen-Truong, 2018 ▸; Suzuki *et al.*, 2014 ▸; Ottosson *et al.*, 2010 ▸). Similarly, the electron elastic scattering is determined by the elastic cross-section (Shin *et al.*, 2018 ▸; Triggiani *et al.*, 2023 ▸) and is related to the electron elastic mean free path (EMFP). Both EMFP and IMFP depend on the kinetic energy of the photoelectron and the transport medium, particularly water (Sinha & Antony, 2021 ▸). The numerical values for the IMFP and the EMFP are still uncertain even for pure water (Nguyen-Truong, 2018 ▸; Sinha & Antony, 2021 ▸). However, for *K*_e_ ranging from 200 to 2000 eV, predictions for the IMFP and the effective attenuation length (EAL) align reasonably well with experimental measurements (Nguyen-Truong, 2018 ▸; Sinha & Antony, 2021 ▸). The PE signal decays exponentially with the depth of origin of the PEs, which makes XPS a highly surface-sensitive measurement technique.

XPS has been successfully applied for aqueous solutions in the form of a liquid microjet (LJ) (Winter, 2009 ▸), with a high curvature mimicking the geometry of droplets. This has been used to investigate solutions of atmospherically relevant concentrations and compounds such as alcohols (Walz *et al.*, 2015 ▸, 2016 ▸; Kirschner *et al.*, 2021 ▸), amines (Ekholm *et al.*, 2018 ▸; Werner *et al.*, 2018 ▸), carboxylic acids and carboxylates (Ottosson *et al.*, 2011 ▸, 2012 ▸; Prisle *et al.*, 2012 ▸; Werner *et al.*, 2016 ▸, 2018 ▸; Ekholm *et al.*, 2018 ▸), formaldehyde (Ottosson *et al.*, 2008 ▸) and various inorganic salts, including sodium chloride, sodium sulfate, ammonium sulfate and ammonium chloride (Winter, 2009 ▸; Prisle *et al.*, 2012 ▸; Öhrwall *et al.*, 2015 ▸). These LJ XPS experiments have determined the surface-specific compositions and molecular-level structures of aqueous solutions with immediate atmospheric relevance. Consistent with observations of atmospheric halogen chemistry (Braun *et al.*, 2017 ▸), measurements of aqueous KBr and KI solutions showed enhanced halide concentrations at the surface (Ghosal *et al.*, 2005 ▸). The surface propensities of C4–C6 alcohols were found to vary with positional isomerism (Walz *et al.*, 2015 ▸, 2016 ▸) and the chain length of the straight chain alcohols (Walz *et al.*, 2016 ▸). XPS revealed by direct observation that the protonation equilibria between atmospheric organic acid/base conjugate pairs were significantly shifted in favor of the neutral species in the aqueous surface (Werner *et al.*, 2018 ▸). Various organic acids, including decanoic acid (Prisle *et al.*, 2012 ▸), succinic acid (Werner *et al.*, 2014 ▸), propionic and octanoic acid (Öhrwall *et al.*, 2015 ▸) and butyric acid (Werner *et al.*, 2018 ▸) were observed to be even further enhanced at the surface in the presence of ammonium ions. This could have large potential implications for heterogeneous aerosol and cloud chemistry in the atmosphere, due to the high pH sensitivity of many reactions involved (Pye *et al.*, 2020 ▸).

To our knowledge, XPS measurements have not been carried out directly on aqueous droplets of atmospheric interest. Specific surface enhancement of certain species was observed in free-flying water–salt clusters and dry, submicrometre aerosol particles. In solvated, sub-2 nm RbBr clusters, bromide was found to reside closer to the cluster surface than rubidium (Hautala *et al.*, 2017 ▸), Mg^2+^ were found to be strongly enriched at the surface of submicrometre aerosol particles generated from solutions of MgBr_2_ and NaBr mixtures, in contrast to particles generated from solutions containing MgCl_2_ and CaCl_2_ mixtures (Pelimanni *et al.*, 2022 ▸). In submicrometre aerosols with inorganic composition meant to mimic sea-salt aerosols, the surface enhancement of magnesium has been found to depend both on the size of the aerosol particle and the type of organic species in the particle (Patanen *et al.*, 2022 ▸). Measurements on mixtures of artificial sea salt and acetic acid as free-flying, dried particles suggest the formation of a core-shell structure (Unger *et al.*, 2020 ▸), which differs from the observations with electron microscopy on analogous particles deposited on hygroscopic substrates (Ault *et al.*, 2013 ▸; Chi *et al.*, 2015 ▸). XPS measurements have provided evidence of water-mediated chemical changes at the surfaces of submicrometre nanoparticles composed of pure NaCl, malonic acid or sucrose, deposited onto a substrate below the respective particle deliquescence points (Lin *et al.*, 2021 ▸).

Direct, surface-sensitive measurements such as XPS are necessary to independently validate modeling approaches to estimate the surface composition of aqueous droplets. Several thermodynamic models have been developed to describe bulk/surface partitioning of solutes in atmospheric models. They can be broadly classified according to the equation of state employed to relate thermodynamic variables and the treatment of the surface as an idealized Gibbs dividing surface or a finite mono- or multi-layer (Malila & Prisle, 2018 ▸). Recent efforts have also included the effects of the formation of aggregate structures such as micelles on droplet surface properties (Calderón & Prisle, 2021 ▸). Vepsäläinen *et al.* (2022 ▸) have compared the most commonly used thermodynamic surface frameworks and found large differences in their predicted cloud droplet forming potential of surface active particles. So far, the applicability of these thermodynamic models has been limited by the availability of experimental parameters on relevant systems needed to constrain the models (Prisle, 2021 ▸).

Molecular dynamics (MD) approaches have been utilized in conjunction with XPS experiments to estimate the composition of aqueous surfaces of atmospheric interest, including inorganic species such as NaI (Ottosson *et al.*, 2010 ▸), bromine (Gladich *et al.*, 2020 ▸), potassium fluoride (Brown *et al.*, 2008 ▸) and organic species such as pentanol (Walz *et al.*, 2015 ▸, 2016 ▸), mixtures of butyric acid and *n*-hexyl amine (Werner *et al.*, 2018 ▸), orcinol and resorcinol (Yang *et al.*, 2022 ▸), oleic and stearic acid (Stewart *et al.*, 2022 ▸), octanoic acid and sodium octanoate (Dupuy *et al.*, 2022 ▸) and tetrabutylammonium iodide (Winter *et al.*, 2004 ▸). Small particles of atmospherically relevant size and composition (organic and inorganic compounds) have been simulated to reveal several types of surface enrichment (Karadima *et al.*, 2017 ▸, 2019 ▸). XPS measurement for aqueous solutions similar to those simulated can then be used to validate MD simulated depth profiles. However, for realistic atmospheric aerosols, potentially made up of more than thousands of organic compounds (Donahue *et al.*, 2011 ▸), MD simulations are computationally intense and difficult to perform. Furthermore, parameterizations necessary to describe the molecular interactions in the system, especially for atmospherically relevant multicomponent solutions, are typically not available.

The depth into the solution where the transition from surface to bulk chemistry occurs is key to further increasing our understanding of atmospheric aqueous surfaces. This requires knowledge of the radial density profiles of species that have a depth distribution different from that of the water. For most aqueous solutions containing multiple species, it is currently not known whether the observed surface enhancement is due to differences in the density profiles of the various species or competition for limited surface sites between species with different surface propensities (Prisle *et al.*, 2012 ▸; Werner *et al.*, 2014 ▸, 2018 ▸; Öhrwall *et al.*, 2015 ▸). It is possible to derive relative density profiles using angular-resolved XPS, as demonstrated for solutions containing environmental organic compounds (Dupuy *et al.*, 2022 ▸, 2023 ▸), and in particular organosulfates (Lewis *et al.*, 2019 ▸). For these mixtures, the relative surface abundances of co-solutes observed with XPS was found to originate from different peak intensity, rather than peak depth, of the radial density profiles with respect to the surface. Unfortunately, these angular-resolved measurements are prohibitively time-consuming for the characterization of a wide range of systems. Depth profiles were obtained for alkali halides in aqueous solutions by changing the X-ray photon energy to yield PEs originating from either the aqueous surface or bulk (Ghosal *et al.*, 2005 ▸; Ottosson *et al.*, 2010 ▸), but required additional ion density profiles from MD simulations (Dupuy *et al.*, 2021 ▸).

For thin liquid films, inversion methods for angular-resolved XPS data have been devised based on regularized least-squares using relative intensity ratios (Eschen *et al.*, 1995 ▸; Baschenko, 1991 ▸), and applied to determine the behavior of ternary systems (Pohl *et al.*, 2013 ▸). There, the method generates possible concentration profiles with the genetic algorithm. The measurement model is discretized in layers assuming piecewise constant concentration in each layer. For solids, one inversion methodology relies on inverting the Laplace transform using a series of homogeneous layers (Bussing & Holloway, 1985 ▸) where the ill-posedness of the reconstruction problem is emphasized. This means that many profiles give rise to the same data. The measured data, normalized peak area, must meet criteria so that depth profiles can be reconstructed. Depth profiling can only be carried out if the normalized peak area is monotonically increasing with respect to the attenuation length (Roberts *et al.*, 2009 ▸). The methodology was improved by introducing a maximum entropy method relying on the Bayesian framework (Macak, 2011 ▸; Smith & Livesey, 1992 ▸). The estimated signals satisfy several criteria, including data fidelity (the difference between the measurement and the theoretical prediction using the estimate and the measurement model) and the maximum entropy principle. Other methods based on the Bayesian framework have been developed for the angle-resolved XPS data acquired for solid samples (Paynter, 2009 ▸; Livesey & Smith, 1994 ▸; Szklarczyk *et al.*, 2017 ▸). Related works for analyzing the surface depth composition of solid matter using XPS or Auger electron spectroscopy have been devised (Tougaard, 2021 ▸).

Other experimental setups have been used for studying thin film liquid samples such as those in formamide solution (Wang & Andersson, 2011 ▸; Wang & Morgner, 2011 ▸) and ethylene glycol (Baschenko *et al.*, 1993 ▸), with different constraints and optimization algorithms used for reconstruction. The prevalent approach does not directly reconstruct the depth profile but instead simulates profiles that are consistent with the experimental data using MD. Depth profiling for bulk liquid samples, therefore, remains an open question, in particular for atmospherically relevant aqueous organic solutions.

Here, we introduce PROPHESY (Ozon *et al.*, 2023*a* ▸), a method for the reconstruction of absolute, quantitative and non-isotropic concentration depth profiles using experimental XPS data. The framework is based on Bayesian inversion and requires the raw spectra, peak areas, effective attenuation length of the PEs and the geometry of the sample (such as sample radius and height of the illuminated area), as well as the typical experimental conditions, including photon flux, transmission function, sample bulk concentration, elemental total cross-section and alignment parameter. We first introduce the measurement model in Section 2 and detail the assumptions. In Section 3 we present the optimization model and the numerical details. The results of numerical experiments are described in Section 4, in Section 5 we discuss the assumptions and the potential limitations of the model, and we conclude with highlights of the work in Section 6.

## 2. Forward modeling

### 2.1. Introduction of the model

**Figure 1:**
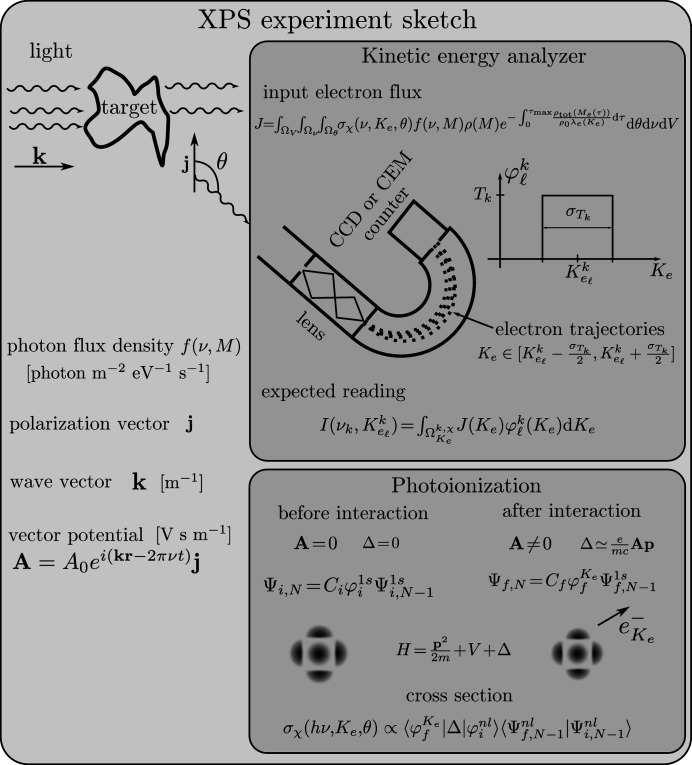
Sketch of the principles of an XPS experiment. A target is irradiated by a photon beam, some of which interacts with matter. From the interaction, PEs are being emitted in every direction following a probability distribution defined by the photoionization cross-section. The cross-section is defined from the initial Ψ_*i*,*N*_ and final Ψ_*f*,*N*_ states of the system made up of the molecules containing the element under investigation. The notation (Hüfner, 2003 ▸) implies that the system has *N* bounded electrons before the interaction with the photon and *N* − 1 after. The state of the photoelectron before the interaction 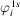 is bounded and after interaction 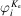 is free with kinetic energy *K*_e_. Only one photoelectron is emitted at a time per molecule. The portion of PEs emitted in the direction of the aperture can be detected by the kinetic energy analyzer. The kinetic energy interval covered by the analyzer depends on the targeted element and the energy of the photon, and it is limited compared with the energy range of all possible PEs.

A sketch of an XPS experiment (Winter & Faubel, 2006 ▸) is depicted in Fig. 1 ▸ where the fundamental components are the photon beam (the light), the probed sample (producing the electron flux) and the measurement device (the kinetic energy analyzer) (Roy & Tremblay, 1990 ▸).

The probing light is characterized by a photon flux density profile (Fedoseenko *et al.*, 2003 ▸; Kachel, 2016 ▸), a vector potential (Meis, 2014 ▸) and an energy whose spread defines the quality of the monochromaticity. The interaction between the beam and the matter generates a PE flux. The signal from the sample is represented using the Beer–Lambert (Paynter, 1981 ▸) model integrated over the volume of the sample Ω_V_ where the signal is proportional to the local concentration ρ(*M*) of effective emitters and attenuated along its way in the sample by all the species in the solution with concentration ρ_tot_(*M*). In the Beer–Lambert attenuation model for XPS, the characteristic length, which we assumed to be the effective attenuation length λ_e_ [m], strongly depends on the kinetic energy *K*_e_ [eV] (Suzuki *et al.*, 2014 ▸; Thürmer *et al.*, 2013 ▸; Ottosson *et al.*, 2010 ▸). The values of the EAL are not precisely known even for pure water (Suzuki *et al.*, 2014 ▸; Nguyen-Truong, 2018 ▸; Sinha & Antony, 2021 ▸), and the values reported by Garcia-Molina *et al.* (2017 ▸) and Tanuma *et al.* (1994 ▸) suggest that they depend on the composition of the sample. We consider the case of a dilute solution for which the attenuation is governed by the solvent, and we consider that even at the interface the EAL is that of the solvent. This assumption introduces modeling errors, especially for samples with strong surface-enhanced species; however, we believe that the model captures the predominant phenomena generating the PE signal and we discuss this assumption in Section 5. The PE signal measured by the kinetic energy analyzer (Wicks & Ingle, 2009 ▸) is represented by the half-circle shape in Fig. 1 ▸. In a simplified approximation, the portion of a PE signal for a given kinetic energy 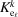 is a weighted sum of the signal around the predefined 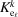 of the measurement (Popović *et al.*, 2017 ▸). The weight function 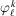 is often termed the point spread function or efficiency of the device. The analyzer has a limited spatial extent, and therefore the PEs in a given solid angle can be detected. The quantity of such PEs is determined by the differential photoionization cross-section density (Cooper, 1962 ▸; Manson & Cooper, 1968 ▸) σ_χ_(ν, *K*_e_, θ) [m^2^ eV^−1^ sterad^−1^], a function determined by the initial Ψ_*i*,*N*_ and final Ψ_*f*,*N*_ states of the system, *e.g.* a carbon atom, where the initial state has only bounded electrons and the final state has one free electron. The cross-section σ_χ_(ν, *K*_e_, θ) depends on the energy *h*ν [eV] of the exciting photon, the kinetic energy *K*_e_ of the emitted electron, the angular direction (θ, see Fig. 1 ▸) of emission relative to the polarization vector of the light and the considered elemental orbital χ, *e.g.* χ = C 1*s*. The kinetic energy spread of σ_χ_(ν, *K*_e_, θ) depends on the central element (Yeh & Lindau, 1985 ▸), in the case of aqueous organic mixtures, *e.g.* C, O, *etc*, as well as on the core-hole lifetime (Nicolas & Miron, 2012 ▸; Ohno & van Riessen, 2003 ▸) and its environment (Toffoli *et al.*, 2007 ▸), *e.g.* neighboring atoms and electrons.

The photoionization cross-section (Cooper, 1962 ▸; Manson & Cooper, 1968 ▸; Hüfner, 2003 ▸) depends on the interaction potential Δ [eV] in the Hamiltonian, which is a perturbation approximated as first order in the vector potential **A** [V s m^−1^] (see Fig. 1 ▸). The cross-section also depends on the electronic configuration (Hüfner, 2003 ▸) and the non-central nature of the resulting potential making the eigenvalues of the system spread around preferred values. The environment of the electron determines the shape of the cross-section and shifts relative to well defined elemental (isolated element) binding energies (Patanen *et al.*, 2013 ▸) which are observed experimentally (Werner *et al.*, 2018 ▸; Öhrwall *et al.*, 2015 ▸; Lin *et al.*, 2021 ▸). The oscillation observed in the total photoionization cross-section σ_χ_(ν) in relation to the photon energy (Björneholm *et al.*, 2014 ▸; Söderström *et al.*, 2012 ▸; Mårtensson *et al.*, 2013 ▸; Travnikova *et al.*, 2019 ▸) cannot be predicted through the elemental calculation alone (Yeh & Lindau, 1985 ▸). This granularity can only be simulated by considering the element environment, such as the neighboring atoms within a molecule.

The total differential photoionization cross-section σ_χ_(ν, θ) [m^2^ sterad^−1^] represents the sum of the density σ_χ_(ν, *K*_e_, θ) over the kinetic energy space and exhibits a dependence on the angle θ between the polarization vector of the light and the direction of the emitted PEs (Seabra *et al.*, 2005 ▸). In the dipole approximation, the angular dependence is determined by the asymmetry factors (Seabra *et al.*, 2005 ▸; Winter & Faubel, 2006 ▸; Yeh & Lindau, 1985 ▸). At the so-called magic angle, 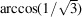 ≃ 54.7, the dipole approximation becomes independent of the asymmetry factors (Ottosson *et al.*, 2010 ▸; Thürmer *et al.*, 2013 ▸).

The multi-electron wavefunction (Ψ_*i*,*N*_ and Ψ_*f*,*N*_) depends on the momentum and the potential energy determined by the local environment. The environment can range from a simple setup such as an isolated atom to a more intricate system like a molecule. The complexity of the surroundings is responsible for the kinetic energy spread. In the case of an isolated atom, the binding energies are sharply distributed, *i.e.* almost quantized if the core-hole lifetime is negligible (Nicolas & Miron, 2012 ▸; Ohno & van Riessen, 2003 ▸). The difference between the photon energy *h*ν and the kinetic energy *K*_e_ of the PE is the binding energy. However, in a more complex system, the binding energies, which are negative eigenvalues of the Hamiltonian, cover ranges of energy around the discrete values of each elemental species found in the system.

### 2.2. Signal of interest: mathematical representation

We model the PE *J*(ν, *K*_e_) [electron s^−1^] signal emerging from the sample and reaching the aperture of a spectrometer using the model from Ozon *et al.* (2023*b* ▸), 

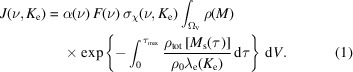

where α(ν) [m^−2^] is the alignment parameter (Ozon *et al.*, 2023*b* ▸), *F*(ν) [photon s^−1^] is the total photon flux at frequency ν [s^−1^], σ_χ_(ν, *K*_e_) [m^2^ eV^−1^] is the photoionization cross-section density for a photoelectron with kinetic energy *K*_e_ [eV] (Toffoli *et al.*, 2007 ▸; Nicolas & Miron, 2012 ▸; Ohno & van Riessen, 2003 ▸) and ρ(*M*) [m^−3^] is the concentration of the target orbital at the location *M* in the sample volume Ω_V_. The attenuation of the PE signal is modeled with the exponential term. In this model, the attenuation occurs along the straight line parameterized with *M*_s_(τ) between *M* = *M*_s_(0) and the point *P* = 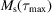 at the aperture of the spectrometer ( 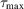 = 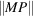 [m]), and is assumed linear with the distance traveled by the PEs. The signal attenuation is characterized by the attenuation length λ_e_(*K*_e_) [m] and by the total concentration ρ_tot_ [m^−3^] whose bulk concentration is ρ_0_ [m^−3^]. We refer to the integral over the volume of the sample Ω_V_ as the geometry factor and denote it *H*(ρ, λ_e_).

The alignment parameter α [m^−2^] mostly accounts for the non-uniformity of the photon beam profile and attenuation of the photon flux in the sample volume Ω_V_, hence this model is equivalent to explicitly modeling the photon beam profile. The parameter α can be interpreted as an average probability density of interaction between the photon beam and the sample.

The attenuation length in this model is the EAL which includes the contributions of elastic and inelastic scattering (Nguyen-Truong, 2018 ▸; Suzuki *et al.*, 2014 ▸; Ottosson *et al.*, 2010 ▸). The EAL is a quantity that is not straightforward to determine either theoretically or experimentally which is a source of uncertainty for the model. The uncertainty in the EAL is in part the motivation for quantifying the errors introduced by the lack of certitude in the EAL. We implicitly assume that the attenuation of the PE signal is governed by the solvent, *e.g.* water, and that it is constant across the sample, *i.e.* the scattering properties do not depend on the spatial location in the sample. This assumption is also a source of uncertainty, especially in the case of concentrated solutions and strongly surface-active substances. We choose to remain in the case for which the total concentration ρ_tot_ is, in absolute terms, predominantly that of the solvent. This is motivated by the fact that for dilute solutions the expected surface enhancement obtained from MD simulations is still small compared with the solvent (Werner *et al.*, 2014 ▸; Minofar *et al.*, 2007 ▸; Mahiuddin *et al.*, 2008 ▸). For instance, in an aqueous solution with a dilute solute at 0.2 *M*, assuming surface enhancement by a factor of ten, the peak concentration is 2 *M*, which is still small compared with the solvent concentration. We believe that a model driven by solvent attenuation can still yield relevant information. When this assumption fails, the model must be modified. We propose a resistive model involving only bulk attenuation lengths in the discussion. The following simplification assumptions were made to formulate the model equation (1): (i) the photon beam profile is uniform and non-attenuated across the sample, (ii) the sample is observed at the magic angle, (iii) the light is monochromatic and linearly polarized, and (iv) attenuation governed by the solvent and the attenuation length is constant across the sample.

The PE signal from samples with spherical or cylindrical geometry (nano-particles/droplets or LJ) is more surface-sensitive compared with the planar case. It is important to consider the geometry of the sample in the model. We show that using the averaged mean escape depth for non-planar geometry does not reflect the modification in the PE signal; in fact, the compensation is more subtle. The focus of the rest of this section is the determination of the attenuation integral 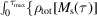 / 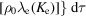 , in particular the limit 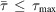 of the parameter of *M*_s_. We assume that the total concentration profile can be approximated by 

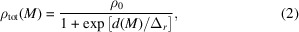

where *d*(*M*) is the signed distance to the surface and Δ_*r*_ is the characteristic transition length associated with the sample. The value Δ_*r*_ determines the thickness of the transition volume, and in practice the integration domain Ω_V_ is limited to the region of space where the distance 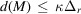 is within κ ∈ [0, 10]. In the limit case, Δ_*r*_ → 0, the volume has sharp edges, *i.e.* the total concentration inside [ 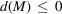 ] the volume is constant at ρ_0_ and 0 outside [*d*(*M*) > 0] the volume. In this case, the integral 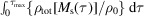 is the distance a photoelectron travels in the sample before leaving the interface towards the analyzer.

**Figure 2:**
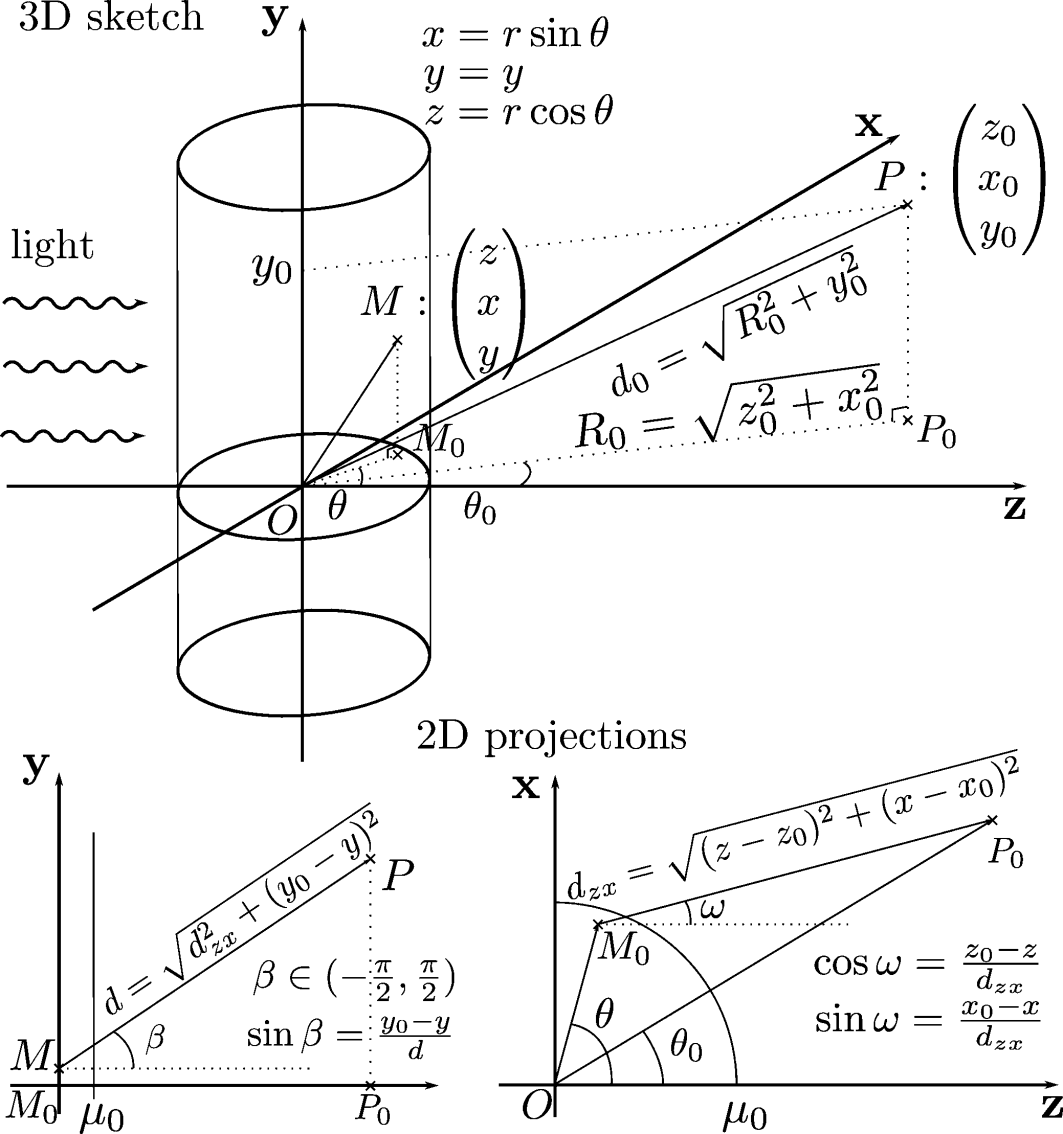
Visual sketch of the cylindrical model. The sample is represented by the section of the cylinder irradiated by the beam. Point *M* is located in the sample and point *P* is on the aperture of the lens of the analyzer. The angular direction (in spherical coordinates) of the line joining *M* and *P* is depicted in the two 2D projections. The complementary polar angle is denoted β and the azimuthal angle is ω. The radial distance *R*_0_ is the projection of the distance between *P* and the origin *O*. If the radius of the sample μ_0_ is such that 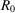 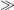 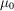 , then the distance *MP* is nearly constant.

Considering any point *M* = (*x*_*M*_, *y*_*M*_, *z*_*M*_) in the liquid and any point *P* = (*x*_0_, *y*_0_, *z*_0_) outside of the liquid, the straight line joining *M* to *P* can be parameterized as 

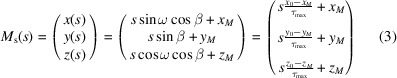

where the direction angles ω and β are depicted in Fig. 2 ▸ and *s* [m] is the parameter of the curve that represents the signed distance from point *M*. The angle ω is between the *z*-axis and the projection *MP* onto the plane *zOx*, and β is taken as the angle between the plane *zOx* and *MP*.

#### 2.2.1. Planar and linear sample

In the planar case, the sample is located in the semi-infinite volume defined by the equation 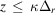 . Because the intensity is exponentially attenuated with the distance, most of the PE signal is coming from the surface layers, such that Ω_V_ can be reduced to the region of space *z* ∈ [−*q*λ_e_, κΔ_*r*_] with 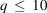 . The depth function is directly *d*[*M*_s_(*s*)] = *z*(*s*) from equation (3). Hence, the planar model is 

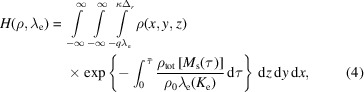

with 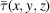 = 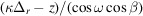 . Note that 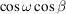 > 0 if the analyzer is not located in the sample. Further, if the illuminated area is pointwise, *i.e.* the photon beam profile [see Section 1 of the supporting information (SI)] 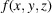 = 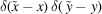 and 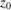 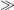 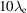 , then 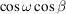 ≃ 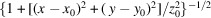 is constant over the surface layer and the model is 

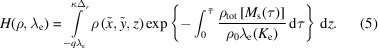

In the limit case, the sharp edge model (Δ_*r*_ → 0), the model simplifies further and the signal takes its usual form, 

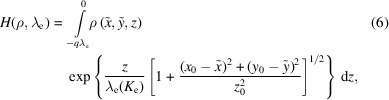

which we refer to as the pointwise model.

#### 2.2.2. Microjet: cylinder approximation

The cylindrical geometry is shown in Fig. 2 ▸. The influence of the cylindrical geometry on the apparent PE attenuation length and further interpretation of XPS data has been rarely addressed (Olivieri *et al.*, 2017 ▸; Dupuy *et al.*, 2021 ▸). The attenuation length λ_e_ is determined by the kinetic energy of the PE; however, the probed depth, or apparent attenuation length, depends on the attenuation length λ_e_ as well as the geometry of the sample.

The propagation direction of the photon beam defines the *z*-axis, the axis of symmetry of the LJ defines the *y*-axis, and the *x*-axis is along the polarization vector supposed to be orthogonal to both other axes. The depth function for the total concentration profile is *d*[*M*_s_(*s*)] = [*x* ^2^(*s*) + *z* ^2^(*s*)]^1/2^ − μ_0_, with the parameterized coordinates from equation (3). The distance 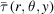 can be found by seeking the intersections between the trajectory and the surface of the cylinder *r* = μ_0_ + κΔ_*r*_. By definition of the parameter *s*, the distance is the positive root of the polynomial,

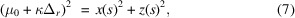

where μ_0_ is the radius of the LJ. Using the canonical polar coordinates, the distance is 

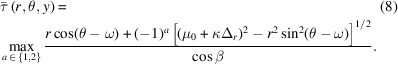

In the extreme case 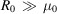 , *i.e.* ω ≃ θ_0_, and 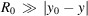 , *i.e.* β ≃ 0, the distance simplifies to 

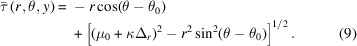

Then, it is necessary to define the integration domain Ω_V_. In polar coordinates, the whole cylinder including the smooth transition layer would be 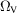 = 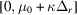 × 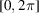 × 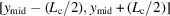 with *L*_c_ the height of the illuminated area and *y*_mid_ its mid-level. The domain can be reduced because only the near-surface layers contribute significantly to the signal similarly to the planar case, and only the side exposed to the analyzer. Indeed, at the surface, the signal is not attenuated [ 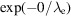 = 1], while deeper in the sample volume the attenuation in strong [ 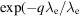 ) = 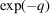 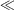 1 for *q* > 2]. Hence, the integration domain becomes 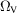 = 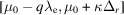 × 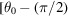 , 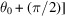 × 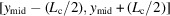 , where the polar angle domain is to be interpreted modulo 2π. Assuming that the concentration ρ is invariant along the *y*-axis and also independent of θ (due to symmetry), one can write ρ(*x*, *y*, *z*) = ρ(*r*). The concentration being invariant along the *y*-axis on the LJ segment used for the measurement, about 5 mm downstream from the nozzle, can be attributed to the aqueous surface stabilizing fast after forming. Using these assumptions, the acquisition model becomes 

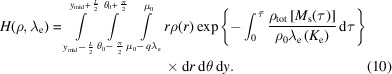

**Figure 3:**
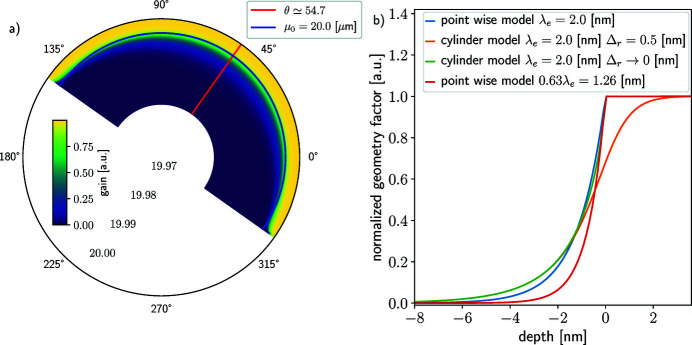
(*a*) Gain of the acquisition model in the near-surface region of the cylinder model with the device set at the magic angle: device far from the sample (*R*_0_ > 100μ_0_). The center of the plot in panel (*a*) is not the center of the cylinder, the near-surface area has been stretched to emphasize the surface sensitivity of the gain. (*b*) Cylinder geometry factor with respect to depth for a uniform illumination (*f* constant): in blue the pointwise model with sharp edge approximation and attenuation length λ_e_ = 2 nm, in green and in orange the cylinder model with the same attenuation length using the sharp and smooth edge approximations, respectively, and in red the pointwise model with the attenuation length replaced with the average mean escape depth ≃ 0.63λ_e_ (Winter & Faubel, 2006 ▸; Dupuy *et al.*, 2021 ▸).

Note that the multiplication by *r* comes from the definition of the infinitesimal volume in polar coordinates d*V* = *r*d*r*dθd*y*. Fig. 3 ▸(*a*) shows that the portion of the sample directly facing the analyzer (θ = θ_0_, *y* = *y*_0_) will produce the signal that comes from the deepest layer of the surface, while the part of the sample which is at an angle will only give information from the top layer. This means that, in the cylinder geometry of the sample, the PE signal is more sensitive to the topmost layer than the deepest layer. Therefore, the apparent probed depth is smaller than the attenuation length and it increases with the radius of the cylinder. As a consequence, the information carried by the signal is mostly coming from the near-surface layers.

Figure 3 ▸(*b*) shows the depth discretization of the geometry factor *H*(ρ, λ_e_) for a uniform illumination [*f*(*r*, θ, *y*) constant] plotted against depth for the attenuation length λ_e_ = 2 nm in three different cases and for the attenuation length λ_e_ ≃ 0.63 × 2 = 1.26 nm corresponding to the average mean escape depth of the geometry (Winter & Faubel, 2006 ▸; Dupuy *et al.*, 2021 ▸). The pointwise model described in Section 2.2.1 is plotted in blue and in red for the attenuation lengths 2 nm and 1.26 nm, respectively. Two cases for the cylinder model are plotted in green and in orange, sharp (Δ_*r*_ = 0 nm) and smooth (Δ_*r*_ = 0.5 nm) edges, respectively. The difference between the cases emphasizes the variation in depth-resolved information obtained from the measurements, even with the same attenuation length of the PE signal. Comparing the pointwise model and the cylinder model under the sharp-edge approximation shows that using the pointwise model to interpret data from a cylindrical sample will lead to an incorrect interpretation of the probing depth that the PE signal comes from, potentially hindering the reconstruction of the concentration profile ρ. Similarly, using the pointwise model with the average mean escape depth (red curve) does not seem to improve the model, rather it seems to underestimate the depth of origin of the PE signal. The reported values for the EAL given by Suzuki *et al.* (2014 ▸) for the cylinder geometry use the correction factor, leading to an underestimated EAL. The comparison between the sharp and smooth edge approximations of the cylinder model shows that a substantial amount of the PE signal originating from the region of space immediately outside the sharp edge delineation surface is not attenuated in the sharp edge approximation. This, if the smooth-edge approximation is to be held as a better description of the liquid–air interface, implies that the sharp-edge model overestimates the signal originating from outside the surface, and hence underestimates the signal emitted inside the sample. However, both approximations for the cylinder model agree on the relative amount of the PE signal emanating from deep in the sample *d*(*M*) < λ_e_. Overall, the models are not equivalent; in particular, the pointwise model should not be used for quantitative data analysis of LJ data. This remark is also valid for planar samples if the illumination is not pointwise, but the effects are less pronounced.

#### 2.2.3. Particle and droplet: sphere approximation

Although no direct XPS measurements on the atmospherically relevant droplets were reported to date, the possibility of the appearance of such experimental stands in the future cannot be ruled out. Here we consider the spherical sample geometry case most closely resembling atmospheric aerosol particles and droplets. The size of the particle or droplet, *e.g.* nano or micro, is not relevant for the model; however, it does matter for the application (Patanen *et al.*, 2022 ▸).

We use the spherical coordinate to describe the location of point *M* in the frame of the particle/droplet, 

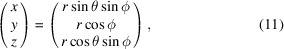

where the azimuth θ is the same as in polar coordinates and ϕ is the polar angle between *Oy* and *OM*. Let ξ be the angle between *MP* and *OM*. Using the same notation as in the cylindrical case, one has

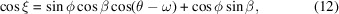

and the distance becomes

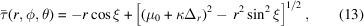

and the depth function is *d*[*M*_s_(*s*)] = ||*M*_s_(*s*)|| − μ_0_.

The integration domain Ω_V_ = [0, μ_0_ + κΔ_*r*_] × [0, 2π] × [0, π] reduces to 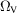 = 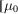 − 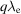 , 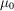 + 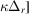 × 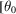 − 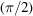 , 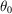 + 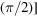 × 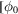 − 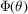 , 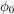 + 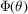 ]; the polar angle must be parameterized with the azimuth θ. The angle domains must account for the definition domains, *i.e.* the azimuth is modulo 2π and the polar angle is in [0, π] which involves potential axial symmetry, in turn modifying the azimuth, hence the parameterization of the polar angle depends on the azimuth. The infinitesimal volume in spherical coordinates is 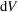 = 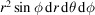 , then, 

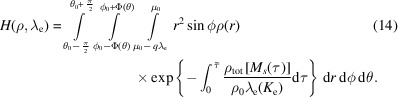

**Figure 4:**
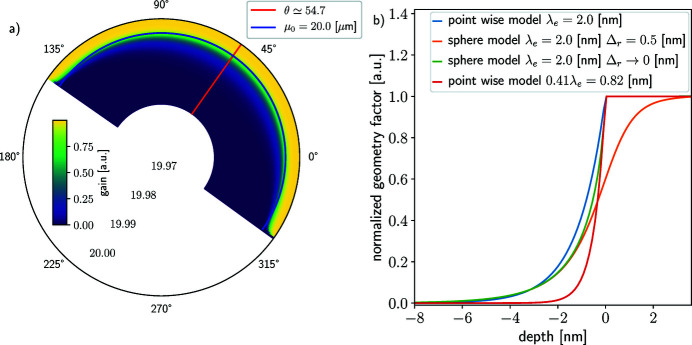
(*a*) Gain of the acquisition model in the near-surface region of the spherical model with the device set at the magic angle: device far from the sample (*R*_0_ > 100μ_0_). The center of the plot in panel (*a*) is not the center of the sphere, the near-surface area has been stretched to emphasize the surface sensitivity of the gain. (*b*) Sphere geometry factor with respect to depth in the case of uniform illumination (*f* constant): in blue the pointwise model with sharp edge approximation and attenuation length λ_e_ = 2 nm, in green and in orange the sphere model with the same attenuation length using the sharp and smooth edge approximations, respectively, and in red the pointwise model with the attenuation length replaced with the average mean escape depth ≃ 0.41λ_e_ (Winter & Faubel, 2006 ▸; Dupuy *et al.*, 2021 ▸).

Figure 4 ▸(*a*) shows the geometry factor described in equation (14) with a uniform illumination [*f*(*r*, θ, ϕ) constant] and Fig. 4 ▸(*b*) shows the gain with respect to depth for a sphere of diameter 20 µm and attenuation length λ_e_ = 2 nm. Overall, the sphere model is similar to the cylinder model; however, the PE signal is even more surface sensitive than its cylinder counterpart. The green and orange curves in Fig. 4 ▸(*b*) represent the sphere geometry factors, with sharp and smooth edge approximation, respectively, which is decreasing faster with respect to depth than the pointwise (and cylinder) model. Similarly to the cylinder model, the sharp-edge model overestimates the signal originating from outside the limit of the sample [*d*(*M*) > μ_0_] compared with the smooth-edge model. However, both approximations lead to very similar models of the signal emanating from the depth below −3 nm. The blue and red curves both show the pointwise model, respectively, for the same attenuation length λ_e_ = 2 nm as the sphere model and for the average mean escape depth λ_e_ ≃ 0.41 × 2 = 0.82 nm. Neither pointwise model is a good approximation for the sphere model. The blue curve is above the green curve at depths in the interval [−3, 0], overestimating the signal originating from these depths. The red curve overcompensates the exacerbation of the surface sensitivity of spherical samples in the range [−6, 0]. Hence, neither pointwise model should be used for quantitative data interpretation of spherical samples.

The probed depth for spherical geometry is even smaller than that of the cylindrical case, making it less favorable for density profile reconstruction. However, the model does not account for the effect of curvature on the density of water at the interface. For the same bulk solution, the transition length Δ_*r*_ may be different between a cylindrical and spherical sample. The lower density of water at the interface implies a smaller attenuation, which may counterbalance the loss of the PE signal in small samples such as nanodroplets. The water density at the liquid–air interface depends on the size of the liquid particle.

### 2.3. Analyzer

In the XPS experiment, the kinetic energy analyzer measures a fraction of the PE signal in equation (1) for *L* predefined kinetic energies 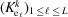 . The resolution of the device is finite, and, for a given kinetic energy, neighboring energies contribute to the measurement which is well approximated by convolution with an efficiency function 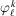 (Popović *et al.*, 2017 ▸; Dupuy *et al.*, 2021 ▸). The expected measurement is 

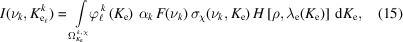

where α_*k*_ = α(ν_*k*_) and 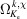 is the kinetic energy interval that covers the support of the photoionization cross-section density σ_χ_(ν_*k*_, *K*_e_). In the most simplistic approximation the spectral blurring effect of the kinetic energy analyzer can be modeled by a rectangle efficiency function with height *T*_*k*_ [s] and width 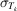 [eV] as depicted in Fig. 1 ▸. More realistic efficiency functions (*e.g.* Gaussian kernel) can be considered; however, this is beyond the scope of this work, and the conclusion is not affected by the choice of efficiency functions. We consider a kinetic energy width 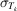 of the same order of magnitude as the distance 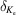 [eV] between two consecutive elements of the collection 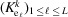 . Furthermore, we assume that the efficiency function 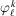 is sufficiently narrow ( 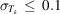 eV) compared with the signal (width ≃ 1 eV), *i.e.* σ(ν_*k*_, *K*_e_)*H*[ρ, λ_e_(*K*_e_)] does not vary much over a kinetic energy range of length 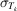 (see SI Section 2.2 for technical details). Therefore the approximate measurement model becomes 

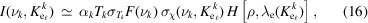

where *T*_*k*_ [s] is the transmission function (Wicks & Ingle, 2009 ▸; Dupuy *et al.*, 2021 ▸), which depends on the kinetic energy, the setup pass energy and the acquisition integration time. The PE signal 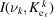 is a count of PEs which is a dimensionless quantity. The formulation (16) is the same as equation (1) in the work of Ottosson *et al.* (2010 ▸); therefore, it introduces the same modeling errors due to the simplifications. Some of the uncertainties can be accounted for during the inversion, either by sampling or marginalizing. In Section 4, we have focused on the uncertainties related to the attenuation length.

### 2.4. Measurement

#### 2.4.1. Spectrum point

By nature, counting PEs is a stochastic process that is achieved with, for example, charged coupled devices (CCDs) or channel electron multipliers (CEMs). A realistic model for the measurement noise is a Poisson distribution either in CCD (Healey & Kondepudy, 1994 ▸; Konnik & Welsh, 2014 ▸) or in CEM (Seah, 1990 ▸; Choi & Kim, 2000 ▸). Here, we consider simulated experiments with electron yields that are sufficiently strong that the Poisson noise distribution can be approximated by an equivalent Gaussian distribution. The photoelectric signal is decomposed into three terms, 

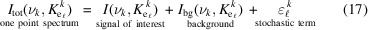

where the noise 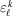 is characterized by 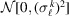 with 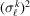 = 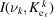 + 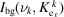 . The cross-section density in this model gives a measure of the probability of a photon interacting with the core-level electrons of chemical species and resulting from different intramolecular interactions (Patanen *et al.*, 2013 ▸). The remaining unknown for σ_χ_(ν_*k*_, *K*_e_) is the kinetic energy dependence, but adding all the contributions from the different kinetic energies leads to the total cross-section 

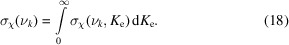

The background signal *I*_bg_ and measurement noise 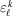 are perturbations of the signal of interest in this study. The background signal stems from other electron interactions, while the noise can be from any source, even external. The background does not contain relevant information for XPS depth profiling and should be removed from the PE signal for data analysis.

#### 2.4.2. Peak area model

For a typical sample system, the XPS spectra show several peaks that can be explained mostly by the different chemical states of the observed element, *e.g.* C–C, C=C, C–O, and the multiplet splitting (Stevie & Donley, 2020 ▸; Major *et al.*, 2020 ▸; Werner *et al.*, 2018 ▸). Each peak in a spectrum thus contains quantitative information on the probability of interaction. For instance, for a system with two chemical states, two peaks are present in the spectrum, and the cross-section density can be written as the sum 

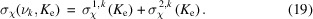

From here, we define the probabilities (*p* ^*m*, *k*^)_*m*_ of interaction photon/chemical-state-*m* with 

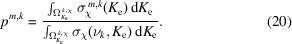

Then we define the probability densities, 

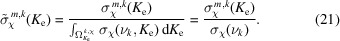

Extending this formulation to the case with 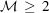 chemical states, the photoionization cross-section density assumes the form 

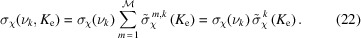

From this definition, the *m*th peak area can be modeled by adding the contribution from each channel, 

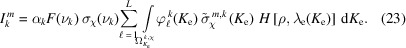

From the spectral data, the peak area can be estimated by peak fitting techniques which are well documented (Kukk *et al.*, 2001 ▸, 2005 ▸; Gengenbach *et al.*, 2021 ▸).

### 2.5. Discretized forward model

From here on, we consider that the cross-section densities have been estimated for each selected peak *m* and for each frequency ν_*k*_, *i.e.* the discretization coefficients 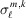 of the photionization cross-section density [see SI equation (7)] are known for each measurement. By compiling the discretized peak area models [see SI equation (14) derived from equation (23)] in a single operator *A* ^*m*^, we get 

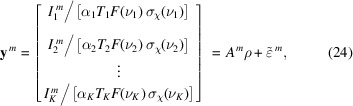

where ρ = [ρ_1_, ρ_2_,…, ρ_*N*_]^*t*^ is the vector of the discretization coefficient of the profile concentration [see SI Section 2, equation (7)], and 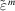 = 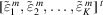 is a 0-mean Gaussian noise vector whose covariance matrix Γ has for diagonal entries the variance of the normalized peak area measurements 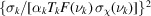 . The variance of the (non-normalized) peak area measurement is 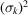 = 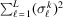 . It is implicitly assumed that the discretization and approximation errors ɛ^*m*,*k*^ [see SI equation (14)] are negligible compared with the measurement noise. The noise level can be determined from the spectra, *e.g.* using the left singular vector of the model to project the data onto (Ozon *et al.*, 2023*b* ▸). The normalized measurement operator for the *m*th peak and all photon energies 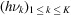 is 

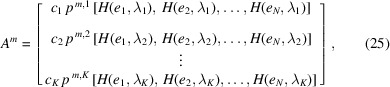

where *T*_*k*_ *c*_*k*_ is the discretization coefficient of the analyzer kernel functions [see SI Section 2.2, equation (13)], and depends on the transmission coefficient *T*_*k*_, the kinetic energy discretization step 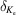 as well as the bandwidth 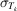 of the kernel functions.

## 3. Numerical methods

An inverse problem (Kaipio & Somersalo, 2006 ▸; Rudin *et al.*, 1992 ▸; Gelb, 1974 ▸) is the opposite of a forward model – it is a model that aims at finding the state ρ of the system knowing the measurements **y** (*e.g.* peak areas) and the forward model *A* ^*m*^ of the experiment and measurement device. In this case, the state ρ is the concentration profile. The measurement model *A* ^*m*^ may refer to either the operator acting on the function ρ or the matrix acting on the vector ρ. The problem of finding the state ρ that leads to the measurement **y** does not have a unique solution; here, many concentration profiles (plausible or not) produce the same data, *i.e.* 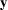 = 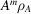 = 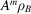 does not imply that 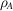 = 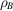 . Therefore, we must formulate the problem to restrict the number of possible solutions. We choose the Bayesian framework because of its versatility and write the probability density 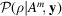 of the state ρ knowing the data **y** and the measurement model *A* ^*m*^. The *a posteriori* 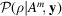 is a measure of how well a concentration profile ρ describes the system – the greater the value 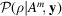 , the better ρ describes the observed system – hence, we seek to maximize the *a posteriori*. Here, the *a posteriori* is modeled as the product of two Gaussian distributions. For counting measurements, or sum of counts, the likelihood 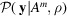 is well modeled by a Poisson distribution, but since the peak area, *i.e.* the parameter ( 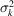 ) of the distribution, is in practice greater than a count of 30, the likelihood is approximated by a Gaussian distribution with variance 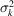 , *i.e.* 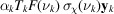 ≃ 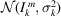 . The *a priori* 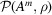 models the knowledge that we have of the system regardless of the data, and it is also modeled as a Gaussian. Since the optimization only seeks for the values of the state ρ, one does not need the data distribution to find the maximum *a posteriori* (MAP) estimate 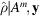 . Overall, the optimization problem can be written as the argument ( 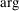 ) that maximizes ( 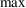 ) the *a posteriori* distribution, formally, 

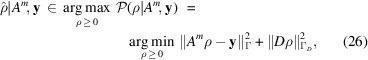

where the positivity constraint is added because, by definition, the concentration is a positive quantity. The formulation (26) implies that we seek the solution (*i.e.* the argument ρ) that maximizes the *a posteriori* probability density [ 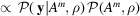 ], earning 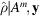 the name Maximum *a posteriori*. The equity between 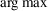 (‘argument that maximizes’) and 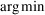 (‘argument that minimizes’) can be shown using the strictly decreasing property of the 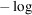 -function which transforms the maximization of the *a posteriori* into a regularized least-squares problem in the case of a Gaussian approximation. The data covariance matrix Γ is diagonal because the noise from the different measurements, *i.e.* peak area, is stochastically independent and the diagonal entries are 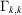 = 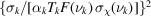 . The variance of the peak area of the *k*th photon energy is 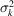 = 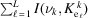 + 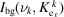 [see SI Section 3, equation (19)]. The regularization operator *D* is the second-order difference operator of order *n*_*d*_ that makes the sought solution smooth by promoting piecewise linear profiles. The covariance matrix Γ_*D*_ defines the strength and correlation related to the regularizing *a priori* [see SI Section (4), equation (27), for numerical definition]. In this form, the optimization problem is not numerically advantageous because it implies reconstructing the profile at each depth of the discretized profile, even though it is expected to be constant below a few nanometres deep, and the boundary value (far out away from the sample) is 0 m^−3^. Therefore, we separate the matrices *A* ^*m*^, *D* and the vector ρ into blocks mapping different depth intervals, *i.e.* bulk, boundary and surface depth, and reorganize the problem to solve only for the surface depth profile. We refer to the rearranged formulation as the truncated model and the technical details are presented in SI Section 5. The boundary ρ_1_ and the bulk values ρ_*B*_ are treated as random variables so as to reflect uncertainty in their values. We solve the optimization problem (26) using an algorithm described in the paper by Chambolle & Pock (2011 ▸) because of its convergence properties and its ease of implementation (see SI Section 6.1 for implementation details).

The MAP estimate 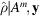 depends on the measurement model and the noise in the data, so, for applicability to experimental data (as opposed to simulated data), it is important to know how much it is affected by these quantities and show that the method is robust enough against measurement noise and attenuation length uncertainty. We define the variability as the covariance with respect to either *A* ^*m*^ or **y**, respectively, 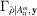 and 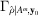 . These two covariances can be estimated by sampling the noise space and the measurement operator space and computing the estimates for each sample,

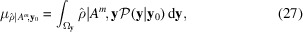




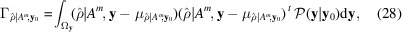




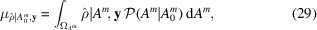




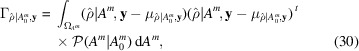

where 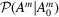 is the probability distribution of the measurement operator knowing the true model 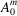 – it reflects the modeling uncertainties. The probability distribution 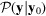 is chosen as the measurement noise distribution instead of the uniform distribution over all possible values of **y** which would be uninformative. Detailed definitions of the probability distributions 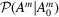 and 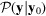 are given in SI Section 4. The integrals are evaluated by means of sampling [from 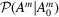 and 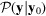 ] because of the low dimensionality of the data space and the parameter space of the measurement model.

In addition to the variability of the state estimates 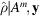 , the model has uncertainties inherent to the posterior distribution. We choose to estimate the *a posteriori* covariance as a proxy for the inverse model uncertainty 

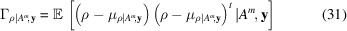

with the *a posteriori* mean 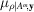 = 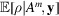 . The covariance matrix is estimated with the Metropolis–Hastings (MH) algorithm that has been described several times independently – Metropolis in 1949 (Metropolis & Ulam, 1949 ▸) and Hastings in 1970 (Hastings, 1970 ▸) (see SI Section 6.2). The uncertainties tied to the noise and measurement models are estimated by the marginalization over the respective spaces. We denote 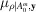 = 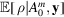 and 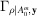 = 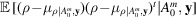 the marginal mean and covariance over the measurement operator space, and 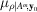 = 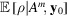 and 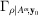 = 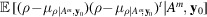 the marginal mean and covariance over the measurement noise space. The covariance 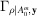 represents the uncertainty due to the uncertainty in the measurement model and 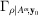 is the uncertainty due to the measurement noise.

The peak area model equation (23) is discretized assuming that the attenuation length is constant over the kinetic energy interval 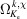 for each photon energy measurement. The discretization is made by projecting the concentration profile ρ and the photoionization cross-section density σ_χ_(ν_*k*_, *K*_e_) onto linear basis functions in their respective spaces. The details of the discretization are given in SI Section 2. The measurement model is discretized over the surface depth 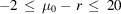 nm; however, the profile reconstruction is carried out over the depth of the truncated model from δ_0_ [nm] to the threshold value δ_*B*_ [nm] whose values depend on the profile. Beyond the threshold depth δ_*B*_, the concentration is assumed to be that of the bulk solution ρ_*B*_, and the concentration at the edge *r* = μ_0_ + δ_0_ of the sample is 0. The resulting reconstruction is based on the truncated model, *i.e.* the values of the concentration profile are estimated for *r* ∈ [μ_0_ − δ_*B*_, μ_0_ + δ_0_) (see SI Section 5 for more details).

## 4. Results

We show the performance of the PROPHESY framework, first introduced for estimating the alignment parameter α_*k*_, and completed in Section 3 using the measurement model described in Section 2. As a proof of concept, we simulated data so that the results can be compared with the true profiles – the ground truth (GT). The geometry factor is the main focus because it is the part of the model that bears depth information. Parameters that do not carry depth information are normalized. Hence, α_*k*_ *T*_*k*_ *F*(ν_*k*_)σ_C1*s*_(ν_*k*_)ρ_*B*_ is used as a normalizing factor for the peak area data. Furthermore, the alignment parameter α_*k*_ or the multiplicative factor α_*k*_ *T*_*k*_ can be estimated from raw data (Ozon *et al.*, 2023*b* ▸), supporting the use of absolute peak area, instead of relative peak area ratio such as C 1*s*/O 1*s*. The APE method (part of PROPHESY) can be used to estimate the multiplicative factor α_*k*_ *T*_*k*_ *F*(ν_*k*_)σ_C1*s*_(ν_*k*_)ρ_*B*_ which includes the photoionization cross-section of C 1*s* in the chemical state of interest, *i.e.* the cross-section including the oscillations (Björneholm *et al.*, 2014 ▸; Söderström *et al.*, 2012 ▸; Mårtensson *et al.*, 2013 ▸; Travnikova *et al.*, 2019 ▸). Four plausible concentration profiles (Krisch *et al.*, 2007 ▸; Jungwirth & Winter, 2008 ▸; Winter *et al.*, 2004 ▸; Winter & Faubel, 2006 ▸) are considered: one that is completely depleted at the surface, 

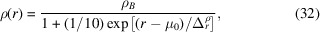

with 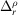 = 0.1 nm, and three that show surface enhancement, 

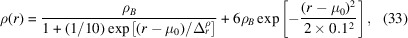




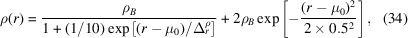




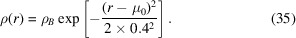

We investigate three aspects of the model that affect the profile reconstruction: (i) the accessible range of attenuation lengths, (ii) uncertainty in the attenuation length and (iii) measurement noise. We choose the transition length parameter Δ_*r*_ = 0.25 nm for the total concentration ρ_tot_, equation (2).

To prevent the inverse ‘crime’ (Kaipio & Somersalo, 2006 ▸) and avoid unrealistically good reconstructions, the measurement model used for the profile reconstruction is not the same as the one used for generating the data. The measurement model *A* ^*m*^ used for the inversion is computed with different discretization than that used for data simulation.

The characterization of the MAP reconstruction equation (26) with respect to the model uncertainty is carried out by randomly sampling the measurement model from the distribution 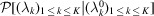 (see SI Section 4) where 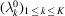 are the attenuation lengths used for simulating the data and computing the reconstruction for each model sample. The marginalization (estimate 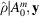 and covariance matrix 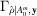 ) are computed from the reconstructions obtained with the model samples. Similarly, the marginalization (estimate 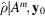 and covariance matrix 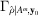 ) over the measurement noise space is computed from reconstructions obtained with data samples drawn from 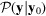 (see SI Section 4).

Here, we focus on PEs with kinetic energy in the interval [200,1600] eV for which plausible EAL is in the interval [1.28, 5.5] nm. The distribution 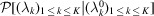 reflects the uncertainty in the current knowledge of EAL. For instance, the EAL and IMFP reported in several studies show that their predicted values agree within some uncertainty range which can be used to build 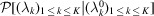 (Suzuki *et al.*, 2014 ▸; Shino­tsuka *et al.*, 2017 ▸; Nguyen-Truong, 2018 ▸; Emfietzoglou & Nikjoo, 2007 ▸; Garcia-Molina *et al.*, 2017 ▸). In the case of a semi-empirical model (Emfietzoglou & Nikjoo, 2007 ▸), the distribution can be built upon the parameters used for fitting the model to the data. The support, *i.e.* the interval where the function is not null, of the attenuation length distribution is the interval 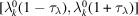 where τ_λ_ is the uncertainty rate.

The amplitude of the *a priori* in the optimization problem (26), *i.e.* the norm of the covariance matrix Γ_*D*_, is controlled by a scalar value σ_*D*_ (see SI Section 4). The values for σ_*D*_ can be determined from criteria such as the L-curve (Stolzenburg *et al.*, 2022 ▸) so that the choice is more objective than ours. Throughout the numerical experiments we use the regularization parameter listed in Table 1 ▸. The correlation length δ_*D*_ controlling the amplitude of the off-diagonal elements of Γ_*D*_ is 0.22 nm which corresponds to the depth discretization step.

### 4.1. Attenuation length sampling domain

**Figure 5:**
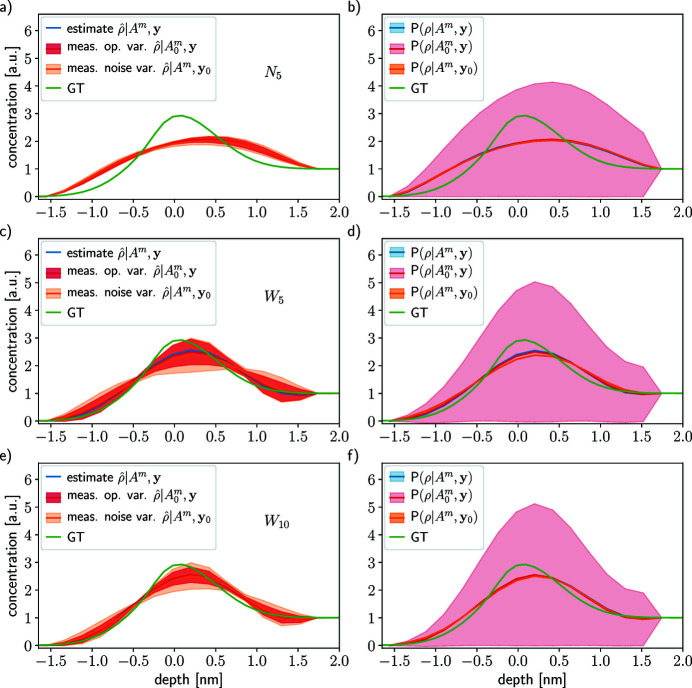
Reconstruction of concentration profile for three different simulated experimental acquisition setups: (*a*) and (*b*) five attenuation lengths over the range [1.62, 1.95] nm (*N*_5_); (*c*) and (*d*) five attenuation lengths over the range [1.28, 5.5] nm (*W*_5_); and (*e*) and (*f*) ten attenuation lengths over the range [1.28, 5.5] nm (*W*_10_). The panels (*a*), (*c*) and (*e*) show the estimates and the different variability, with respect to the measurement noise in orange ( 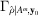 ), and with respect to the measurement model error in red ( 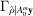 ). The panels (*b*), (*d*) and (*f*) show the conditional posterior probability 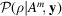 (blue), and the marginals 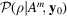 (orange) and 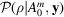 (red).

Figure 5 ▸ shows the results of the inversion model using the framework PROPHESY applied to simulated data using the density profile plotted in green. In this example, the GT profile [equation (34)] vanishes 1.5 nm away from the surface and reaches bulk concentration before 1.5 nm into the sample. For the sake of characterization of the methodology, we consider a wide interval of attenuation length from 1.28 nm to 5.5 nm, even though it does not correspond to technologically accessible values. The reason for this choice of interval is to show what can be gained (or not) by measuring at attenuation lengths that might not be considered. Three probing cases are considered, simulating three possible acquisition setups, one using ten attenuation lengths and two using only five. The attenuation lengths are regularly sampled in the ranges [1.62, 1.95] nm, case *N*_5_, and [1.28, 5.5] nm, case *W*_5_, for the cases with five attenuation lengths, and in the range [1.28, 5.5] nm for the case with ten, case *W*_10_. The case *W*_10_ is used as the default setup in the remainder. In the three cases, the noise level is representative of experiments (see Section 4.3), and the uncertainty in the attenuation length is low (independent error level 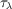 = 0.5%, see Section 4.2.1).

In the left column of Fig. 5 ▸, the estimates resulting from the optimization problem (26) are depicted in blue and the variability with respect to the measurement operator and acquisition noise are shown in red and orange, respectively. As expected, the profile reconstruction is better in the case *W*_10_ than in the cases *N*_5_ and *W*_5_: more information and denser sampling are better than less information and sparser sampling.

The MAP estimates 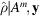 (blue curves) obtained from the inversion model equation (26) and the marginalizations 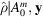 (red curves) and 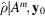 (orange curves) capture the structure of the concentration profile in the cases *W*_5_ and *W*_10_ contrary to the case *N*_5_. This is visible for profile equation (34) as well as other profiles shown in SI Section 7.1. In all cases (*N*_5_, *W*_5_ and *W*_10_) the variabilities 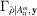 (red shaded areas) of the estimates reflect the low level 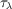 = 0.5% of uncertainty in the attenuation length – the red shaded areas are small. The noise level is also reflected in the variabilities 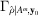 (orange shaded areas), in particular for the cases *W*_5_ and *W*_10_. In the case *N*_5_, the depth information in the data is very little, therefore most of the reconstruction is carried out by the *a priori*.

For both variabilities ( 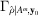 and 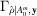 ), nodes appear, which seems to indicate that the concentration at some depths is reconstructed better than in others depending on the choice of probed attenuation lengths. Depending on the concentration profile, the node locations vary; however, in the cases *W*_5_ and *W*_10_, they are located just before and after the structure (peak or step) of the profile. The reason for this is not clear, but the information is not uniformly sampled even though the attenuation length is.

In the right-hand columns in Fig. 5 ▸, the mean and variance of the different probability distributions are depicted: 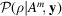 in blue, the marginalization over the noise space 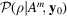 in orange, and the marginalization over the measurement operator space 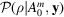 in red. The conditional means μ_ρ|•_ and covariances Γ_ρ|•_ do not need to coincide with the true profile since the estimate corresponds to the maximum of the conditional, not the mean. The two marginals are broader than 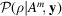 , but the effect is striking for the marginalization over the measurement operator space. The strong broadening of 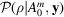 shows that the measurement model is highly sensitive to the values of the attenuation length, while the weak broadening of 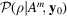 shows that the model is not too sensitive to the noise in the data. This means that a small deviation in the data can easily be accommodated by the model since the *a posteriori* is built upon the statistical model of the measurement noise, whereas a small error in the model parameters is not straightforwardly accounted for and might compromise the reconstruction process. However, and contrary to the model, the estimate 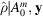 sampled with respect to the attenuation lengths does not exhibit strong variability, and the sensitivity of the inversion model is decreased by increasing the number of sampled attenuation lengths.

Overall, Fig. 5 ▸ shows that more numerous attenuation lengths probed over a wider range is better than few probed λ_e_ over a narrow range. These results hold for all the tested concentration profile equations (32), (33) and (35) (see SI Section 7.1, Figs. 2, 3 and 4, respectively, therein). We advise probing as many attenuation lengths as possible in the widest range for depth profiling.

### 4.2. Model error: λ_e_ uncertainty

In the forward model, the attenuation length is the EAL of the solvent, assuming that the attenuation is governed by the solvent and that the boundary effects at the interface are negligible. We assume this to be representative of dilute solutions, even though it does not capture the whole complexity of the signal formation at the interface. For pure water, the EAL and IMFP are still under investigation and their values are subject to debate (Suzuki *et al.*, 2014 ▸; Nguyen-Truong, 2018 ▸). Furthermore, the values of predicted and measured EAL and IMFP suggest that they depend on the composition of the solution (Garcia-Molina *et al.*, 2017 ▸). Therefore, for the reconstruction of depth profiles, it is not clear what values should be used, which is a source of uncertainty. As a consequence, we suggest using plausible values as well as their uncertainty range as a starting point to characterize the effect on the reconstruction. In this section, we consider two types of errors, (i) small independent errors representing the granularity not captured by current models, and (ii) a large global error accounting for the disagreement between models, *i.e.* the slope of the EAL model. More refined error models could be investigated, such as that proposed in SI Section 4 based on a semi-empirical model; however, for the sake of clarity we choose to focus on these two limit cases.

#### 4.2.1. Independent errors

We consider the case of small independent perturbations in the values of the attenuation lengths, to reflect the small variations of values not captured by current EAL (and IMFP) models, *e.g.* experimental fit using smooth exponential-polynomial functions (Emfietzoglou & Nikjoo, 2007 ▸). The model error probability for levels 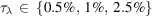 are modeled using 

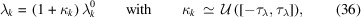

where the random variables 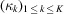 are independent and uniformly distributed.

**Figure 6:**
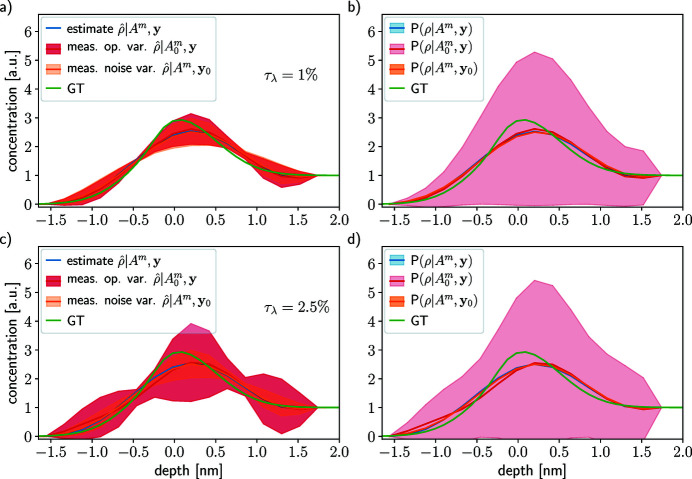
Profile reconstruction in the case of *W*_10_ for two levels of attenuation length uncertainty: (*a*) and (*b*) 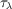 = 1 and (*c*) and (*d*) 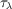 = 2.5%. The green curves represent the GT. In panels (*a*) and (*c*), the profile reconstructions are plotted in blue ( 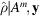 ), orange ( 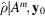 ) and red ( 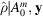 ) with their respective variabilities as shaded areas. In panels (*b*) and (*d*), the *a posteriori* [ 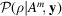 ] is represented in blue, and the marginals in orange [ 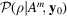 ] and red [ 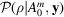 ].

Figure 6 ▸ shows two profile reconstructions in the case of *W*_10_ with higher levels ( 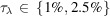 ) of uncertainty in the attenuation length λ_e_ than in Fig. 5 ▸. The MAP estimates 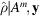 (blue curves) and the measurement noise marginal estimates 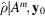 (orange curve) and their variabilities 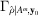 (orange shaded area) are the same as for the *W*_10_ case in Fig. 5 ▸ because these quantities were evaluated with the true value of the attenuation length. The quality of the marginalization 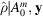 of the estimates is the same across the different uncertainty levels despite minor fluctuations. However, the variabilities 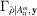 are different for each uncertainty level, and the norm of the variability increases with the level of uncertainty [ 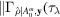 = 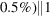 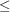 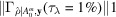 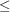 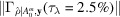 ]. The width of 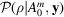 is not affected by the level of uncertainty τ_λ_ even though fluctuation is observed due to the sampling nature of the estimation of the covariance matrices 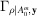 . The sensitivity of the inversion model does not increase with τ_λ_, but the variability does. The same results are observed for all the tested profiles (see SI Section 7.2.1).

#### 4.2.2. Global error

**Figure 7:**
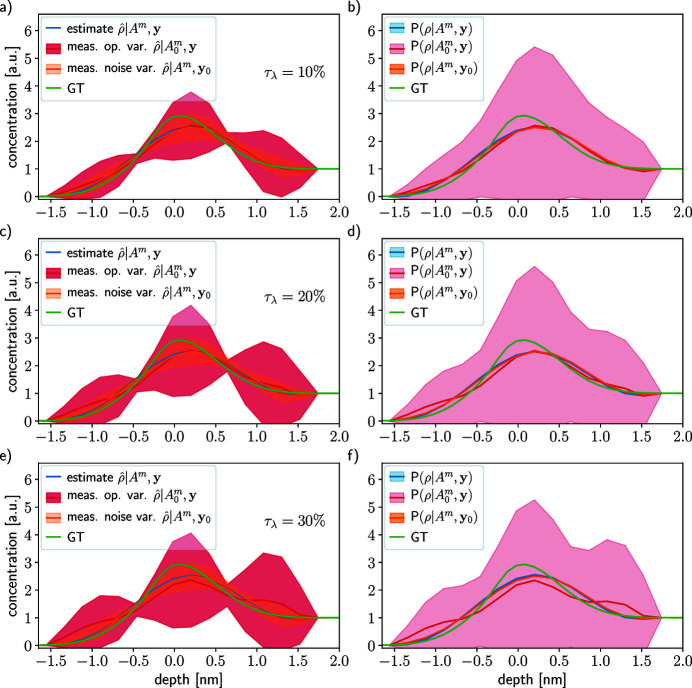
Profile reconstruction in the case of *W*_10_ for three levels of global attenuation length uncertainty: (*a*) and (*b*) 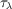 = 10%, (*c*) and (*d*) 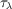 = 20%, and (*e*) and (f) 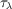 = 30%. The green curves represent the GT. In panels (*a*), (*c*) and (*e*) the profile reconstructions are plotted in blue ( 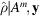 ), orange ( 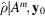 ) and red ( 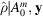 ) with their respective variabilities as shaded areas. In panels (*b*), (*d*) and (*f*) the *a posteriori* [ 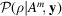 ] is represented in blue and the marginals in orange [ 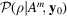 ] and red [ 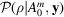 ].

In this section, we consider a different type of uncertainty in the model parameter compared with Section 4.2.1. Contrary to independent errors, the global error represents the possible disagreement between two attenuation length models, *e.g.* model derived from IXS-D2 and IXS-D3 (Emfietzoglou & Nikjoo, 2007 ▸). The purpose of investigating this level of uncertainty is to show that it is still possible to use the inversion model even though large errors in the attenuation lengths are to be expected or if the interpretation of the attenuation length is not clear. We model the global uncertainty with attenuation length generated from 

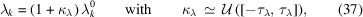

where the same error factor κ_λ_ applies to all *K* attenuation lengths. We consider three levels of uncertainty, 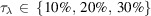


Figure 7 ▸ show the results in the case of *W*_10_ for different uncertainty levels. The estimates 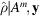 (blue curve) and 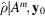 (orange curve) are the same as in Fig. 5 ▸ for the same case. The quantities of interest are the estimates 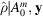 (red curve) and their variabilities 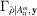 (red shaded area). Similarly to the independent error results, the quality of estimates for each uncertainty level is the same even though some fluctuations appear. The variability increases in norm with τ_λ_ and stays acceptable for surface depths. The probability model 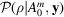 is broad compared with 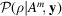 and 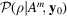 ; however, it does not vary with the uncertainty level τ_λ_. Again, the model is sensitive to the error in the attenuation length values, but the estimates do not vary substantially. The same observation is made for the other profiles (see SI Section 7.2.2).

### 4.3. Data noise

**Figure 8:**
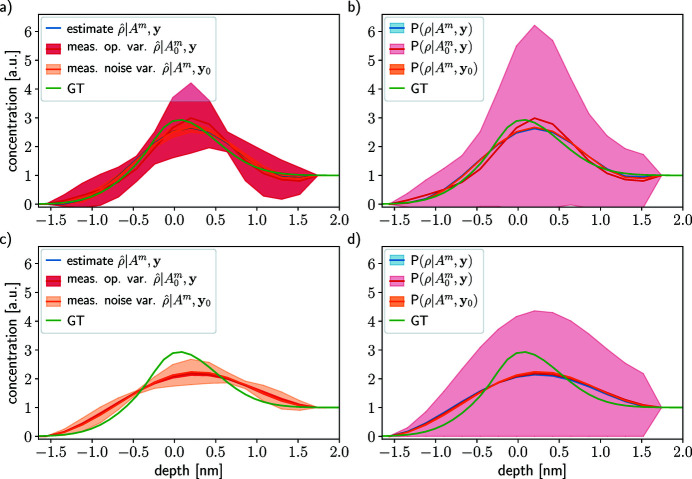
Reconstruction of concentration profiles for two levels of acquisition noise: panels (*a*) and (*b*) very low (σ_*k*_ = 0.01, SNR = 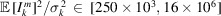 ), and panels (*c*) and (*d*) very high (σ_*k*_ = 0.5, SNR ∈ [100, 6400]). In panels (*a*) and (*c*) the profile reconstructions are plotted in blue ( 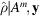 ), orange ( 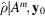 ) and red ( 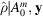 ) with their respective variabilities as shaded areas. In panels (*b*) and (*d*) the *a posteriori* [ 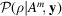 ] is represented in blue and the marginals in orange [ 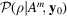 ] and red [ 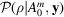 ]. For the noise variability ρ|*A* ^*m*^, **y**_0_ and the noise marginal 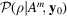 , the true values of λ_e_ have been used to emphasize the effect of uncertain attenuation lengths.

Figure 8 ▸ shows reconstructions for two acquisition noise levels, one lower (σ_*k*_ = 0.01, peak area signal-to-noise ratio (SNR, 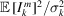 ) ∈ [654 × 10^3^, 43 × 10^6^]) and one higher (σ_*k*_ = 0.5, SNR ∈ [262, 17 × 10^3^]) than that of the reference case *W*_10_ (σ_*k*_ = 0.1, SNR ∈ [6544, 427 × 10^3^]) in Section 4.1 (see Fig 5 ▸). The MAP estimate 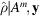 is slightly better at low noise level than at high noise level, and the variability 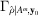 with respect to the measurement noise indicates the same – it is growing with respect to the noise level. On the contrary, the variability 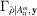 is decreasing with respect to the noise level. This can be explained by a ratio, akin to the SNR, between the error in the model and in the data, *e.g.* 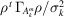 , where 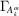 is the covariance matrix of the peak area model for the *k*th measurement, ρ the GT profile and 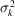 the variance of the noise. The model covariance matrix is defined by 

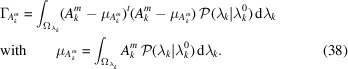

Since the model error level 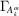 is the same across the cases, and the noise level σ_*k*_ varies, this ratio decreases with respect to the noise level. This means that the contribution to the overall uncertainty in the reconstruction transits from being mostly due to the noise to coming mostly from errors in the model.

The ratio 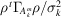 is a proxy for directing our effort for improving the quality of information that can be retrieved from such experiments. At some point, the quality of the data is sufficiently good compared with the quality of the measurement model, and so any improvement in the data quality will not result in any improvement in the reconstruction. For XPS data inversion, the measurement model must be precise to allow for high-quality depth profile reconstruction.

As much as repeating the acquisitions many times is conceptually very simple, in practice it is not always possible to dedicate the time needed for improving the data quality to the point where noise becomes negligible. However, since the *likelihood* model is based on the stochastic acquisition nature of the measurements (*e.g.* Poisson for counting processes), the inversion model can handle reasonably noisy data. Furthermore, sampling the parameters of the model is also (experimentally) time-consuming. For instance, sampling the attenuation length means changing the acquisition setup so that many values ( 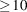 ) are probed over the range of interest. However, it is possible to numerically account for model uncertainties. Nevertheless, it is still necessary to estimate the true level of error (Suzuki *et al.*, 2014 ▸; Nguyen-Truong, 2018 ▸).

## 5. Discussion

### 5.1. Model assumptions

#### 5.1.1. Light

The model devised in equation (1) relies on and reflects a set of assumptions. First, it is assumed that the illumination is monochromatic. It is a good approximation as long as the bandwidth of the photon beam *f*(ν, *M*) is sufficiently smaller than the length-dependent variation of the cross-section density σ_χ_(ν_*k*_, *K*_e_), *i.e.* the range of kinetic energy over which the variation starts being significant. The light bandwidth depends on the setup of the source, but for the sources of interest at synchrotron facilities in the framework of XPS the bandwidths are expected to be in the range between 0.01 eV (low energy) and 0.2 eV (high energy) (Weiss *et al.*, 2001 ▸; Kachel, 2016 ▸). An important parameter that controls the bandwidth is the exit slit opening. For instance, at the HIPPIE beamline (Zhu *et al.*, 2021 ▸), the spectral resolution of the light for a central photon energy *h*ν = 867 eV goes from 15000 to 5000 for exit slit openings of 20 µm and 100 µm, respectively. In this case, the energy spread ranges from 0.06 eV to 0.17 eV. Therefore, for O 1*s* the cross-section density σ_O1*s*_(867, *K*_e_) can be resolved with high fidelity. However, for C 1*s* the exit slit opening is wider. Hence, the cross-section density σ_C1*s*_(867, *K*_e_) cannot be resolved with the same resolution as for O 1*s*. The resolution can be numerically enhanced using a deconvolution algorithm (see Fister *et al.*, 2007 ▸) provided that the photon beam spectrum is known. For typical C 1*s*, the peak width is of the order of 1 eV, therefore even with a spectral resolution of 0.2 eV the peak can be resolved with high precision, and will not severely impact the reconstruction.

The light is assumed to be spatially uniform and non-attenuated by the sample (Berger, 1998 ▸). For instance, a profile example can be found in the work of Fedoseenko *et al.* (2003 ▸) that would make the uniformity assumption hold in one direction (horizontal) and not in the other dimension (vertical). Contrary to the previous assumption, these ones are easily handled by the model, provided that the profile (*f*) of the photon beam is known everywhere in space. For instance, the profile could be acquired with a camera, assuming the absorption by the sample is negligible. However, the uniform light assumption does not hinder the reconstruction process as long as the spot size is comparable with or larger than the diameter of the sample. Under these conditions the model assuming uniform illumination is equivalent to the model including the beam profile (Ozon *et al.*, 2023*b* ▸).

Furthermore, the source is supposed to be perfectly linearly polarized; however, this assumption depends on the quality of the source. The consequence of the degree of linear polarization (Petrova *et al.*, 2019 ▸) is that, even at the magic angle, the cross-section depends on the asymmetry factors. Indeed, the magic angle is defined with respect to the polarization vector (see Fig. 1 ▸), assumed to be unique. Hence, if the polarization is distributed, then so is the magic angle. However, the analyzer is at one location in space, therefore it cannot measure at the magic angle for all polarizations. As a consequence, the cross-section depends on the asymmetry factor of the dipole model. The effect of the imperfection of linearity is expected to be minor because, while the light is not perfectly linearly polarized, the quality is very high (Weiss *et al.*, 2001 ▸; Petrova *et al.*, 2019 ▸; Cant *et al.*, 2023 ▸). Furthermore, the forward model can be modified to accommodate for non-polarized light by substituting the differential cross-section density σ_χ_(ν, *K*_e_, θ) by its angular average.

#### 5.1.2. Core orbitals

For core orbitals, the cross-section is considered to be well modeled by the dipole approximation. Since the acquisition setup takes advantage of the magic angle for which the asymmetry factor does not play a role, then this assumption is weak. However, if photon energy higher than 2000 eV is considered, the dipole approximation starts deviating from measurements (Cant *et al.*, 2023 ▸), which introduces modeling errors. Note that if the acquisition setup changes, then it is possible to change the model to account for asymmetry. The modifications are straightforward to account for the angular dependence if the asymmetry coefficients are known (multiplicative factor depending on the angular direction). Hence, the model is not limited to the core orbitals, although it has here been presented for this specific application. However, the asymmetry coefficients are in practice not well known, in particular for non-isolated non-elemental cross-sections.

In practice, the aperture of the analyzer is not pointwise, rather it covers a range of solid angles, and the magic angle cannot be exactly achieved (or may not be an optimal choice for the experiment). Hence, using the elemental photoionization cross-section at the magic angle inherently adds uncertainty to the forward model. Furthermore, the elemental photoionization cross-section does not exhibit oscillatory behavior contrary to what is observed (Björneholm *et al.*, 2014 ▸; Söderström *et al.*, 2012 ▸; Mårtensson *et al.*, 2013 ▸; Travnikova *et al.*, 2019 ▸) for the target orbitals in complex systems (*e.g.* molecules), which is another source of uncertainty. These modeling errors can be lumped together in the multiplicative factor of the model equation (23) which can be estimated as a whole from raw data (Ozon *et al.*, 2023*b* ▸). Hence, the asymmetry coefficient and oscillation of the photoionization cross-section do not need to be known for practical application of the reconstruction profile.

#### 5.1.3. Geometry

The geometry of the sample is approximated by simple shapes, such as a plane, cylinder or sphere, which allows for invariant assumptions. For instance, in the cylinder case, the concentration is assumed to be invariant along the *y*-axis (microjet symmetry axis) and θ-axis (angle in polar coordinates). To verify these geometry invariances, it is possible to simulate the system. For instance, the invariances can appear out of the simulation of the LJ with fluid mechanics equations, instead of being stated as an assumption based on the symmetry of the sample. However, using the fluid mechanics equations in the canonical frame of reference, *i.e.* cylindrical coordinates, these invariances appear if the diffusion is negligible. These invariances are especially important to make the problem tractable since they reduce the species density ρ to a one-dimensional profile which is a statistical average over the other dimensions.

The smooth-edge model relies on a parametric form of the total concentration profile ρ_tot_ whose shape is an approximation. The only parameter of interest for the forward model in this parametric profile is the width Δ_*r*_. This approximation brings errors to the forward model which translates to potential discrepancies in the reconstruction, particularly if the liquid–air transition length is not well modeled by the parameter Δ_*r*_.

Implicitly, with the forward model we assume that the PE signal is attenuated only due to interactions with water. Furthermore, for dilute solutions, water is assumed to be overwhelmingly predominant, even at the edge. For instance, with a concentration of 200 m*M* of solute in the bulk solution and ten times that at the interface, and considering the peak located where water is half its bulk concentration, water concentration is still much larger (55.5/2 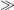 0.2 × 10) than the solute. This means that the total concentration in the model is well approximated by the water concentration. If the bulk solution is not dilute, then this approximation may no longer hold and the total concentration should account for the solute concentration. For instance, in some cases of atmospheric nano-particles, the dilute assumption would fail (Karadima *et al.*, 2017 ▸). Under this assumption, the forward model would be substantially modified, and not only the attenuation length in water should intervene in the model. Additionally, the inversion model would also be modified, and multiple target elements should be used to make the reconstruction possible.

The last significant geometrical assumption pertains to the orientation of the analyzer. It is assumed that the center of the sample, *i.e.* the symmetry axis for a cylinder or center for a sphere, is on the optical axis of the analyzer lens. Failure to meet this assumption means that the distance function is offset, and the analyzer does not see an aligned symmetrical object. From the current model point of view the distance function 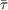 already accounts for potential misalignment, but the analyzer model must now account for the loss of symmetry. The proportional coefficient *T*_*k*_ in equation (16) cannot translate this change, neither can equation (15) since it implicitly assumes (optical) axial symmetry by omitting the angular dependence and spread of the imaged object; in equation (15) the incoming signal is concentrated in a ray on the optical axis. A more comprehensive analyzer model (Guilet *et al.*, 2022 ▸; Wicks & Ingle, 2009 ▸; Seah, 1990 ▸) would account for the different angles.

#### 5.1.4. Inverse model

The measurement model is very low rank because of the very limited number of measurements. However, the inversion is stabilized by assuming that the sought concentration profile belongs to a category of well behaved functions. This is introduced by the smoothness assumption to create an *a priori* that helps to stabilize the numerical process. It entails that if the true concentration profile does exhibit a sharp peak or if it is chaotic (with variations that seem as if they are random), then the method cannot retrieve such a profile. When comparing Fig. 5 ▸ and SI Fig. 3, despite the difference in height and width of the peak, the two retrieved profiles are alike and correspond to a smooth version of the true profile. The smoothness assumption makes inversion problems become numerically stable and it is fairly easy to deploy; however, it also eliminates fine details such as sharp peaks in the concentration profile. One way to overcome the over-smoothing issue would be to have more data and more informative data. For XPS data, this means finding a way to obtain data from different locations in the sample instead of attenuation data. Ideally, make the attenuation { 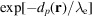 } change in favor of a local excitation, *e.g.* localized light source 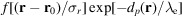 , where the beam profile can be controlled in localization 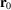 in the surface layers and spread 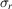 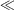 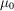 . Note that the localization can either be done by focusing the beam light or using the analyzer lens in a mode that selects a small spot at the surface of the sample. In either case, the number of electron counts would decrease, hence the signal-to-noise ratio would deteriorate.

The strongest assumption in the optimization problem is the known values for certain depths, *i.e.* in the bulk and at the boundary. The difficulty for the bulk concentration ρ_*B*_ is not its value but rather knowing where the bulk starts *r* = μ_0_ − δ_*B*_, *i.e.* determining the thickness value δ_*B*_. In the examples, we chose the value δ_B_ ∈ {1, 1.5} nm depending on the profile, which seems a good cut-off since the concentrations are almost constant from this depth on, and it is still close to the surface layer (region of interest). Similarly, the boundary condition away from the sample, *r* = μ_0_ + κΔ_*r*_, is almost surely 0 for non-volatile compounds, however, the location of the boundary is not clear. Although, it is fair to assume that at 1 nm or 1.5 nm away from the surface the concentration has vanished, depending on the profile.

In the Bayesian model, the likelihood is Gaussian, which is fairly acceptable because of the sources of the noise (Stevie & Donley, 2020 ▸; Wicks & Ingle, 2009 ▸; Watts & Wolstenholme, 2019 ▸) (mixture of several sources, *e.g.* CCD sensor, analyzer, *etc*). However, the probability density for the sparsity in the second-order-difference space has been chosen arbitrarily as Gaussian. Other distributions are acceptable, such as exponential. Another possible regularization would be a learned *a priori* (Lunz *et al.*, 2018 ▸; Li *et al.*, 2020 ▸; Leong *et al.*, 2023 ▸), possibly trained on a set of simulated profiles.

Overall, the strongest assumptions for the design of PROPHESY are:

(i) The geometry approximation of the sample (smooth edge parametric model).

(ii) The values for the attenuation lengths.

However, it is not immediately clear which has the strongest impact on the estimated density profiles.

### 5.2. Investigated uncertainty: attenuation length

Another less direct assumption is the knowledge of the uncertainty in the attenuation length. We only investigated the attenuation length uncertainty effect because it is the most nested and relevant parameter in the model for depth profiling, and also because its value is still an open question. To some extent, the attenuation length can be considered the most meaningful parameter for depth profiling using XPS and similar direct probing. For the parameters α_*k*_, *T*_*k*_, *F*(ν_*k*_) and σ_χ_(ν_*k*_) the uncertainty model is straightforward and can be handled as only one (multiplicative) parameter.

The peak area data are normalized by the estimated values of factors such as the photon flux, in order to make the data independent of the measurement setup. However, the reduced data still bear the uncertainty associated with the normalizing factors. Indeed, while normalizing the data, *e.g.* dividing by the product α_*k*_ *T*_*k*_ *F*(ν_*k*_)σ_χ_(ν_*k*_), the values of the parameters are only known within a range; therefore, the normalization only changes the sources of the uncertainty but does not cancel it. In the non-normalized case the uncertainty is coming from the parameters in the model directly, and in the normalized case these parameters are the true ones (by definition) but the normalized factor is uncertain, hence it introduces the same overall uncertainty. The application to experimental XPS data using relative spectral peak areas is outside the scope of this presentation, but is the topic of ongoing work.

Here, we assumed a distribution for the attenuation length, but the problem is in the parameters of the parameter distribution. For instance, the mean is assumed to be the value at hand while it is actually a value drawn from the true distribution. This has a strong impact on the interpretation of the results. The oscillatory behavior of the concentration profile is, in part, due to the fact that the attenuation lengths are not the right ones, and so the inversion method tries to make the profile fit the data in an (by definition) incorrect way. This setup using the incorrect parameters was willingly chosen in order to observe the effect on reconstructions.

### 5.3. Limitation of the study

The noise is assumed to have the same level σ_*k*_ for each photon energy *h*ν_*k*_, but this is not necessarily the case because of the nature of the counting noise. However, the noise level is representative of the average noise level. In the experimental setup, the noise level in the spectrum can vary considerably between photon energy levels, which translates into different noise levels in the peak area. This trend may impact the quality of reconstruction. However, it is not expected to be significant as long as the noise levels still allow for peak area estimation. The errors in background removal and peak fitting result in errors in the values of the peak areas. These errors are treated as additional noise to the data in the inverse model because they are the results of uncontrolled processes during the fits or errors in the model. In both cases, the errors are random, therefore they are represented by noise and are expected to have the same effect as measurement noise.

We investigated two intervals of error, *i.e.* 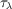 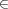 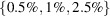 and 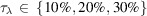 , the first with an error model assuming independence of the errors and the other assuming a global factor in the slope of the EAL model. In both cases the smallest values of τ_λ_ still allow for reconstruction, but for the largest values ( 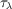 = 2.5% and 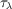 = 30%) the reconstruction shows large variabilities. A more detailed error model for the attenuation length such as that devised in SI Section 4 should be used in order to characterize more precisely the effect on depth profile reconstruction, and we believe this should be the focus of more investigation.

In the forward model, we assume the attenuation length to be a known and well defined quantity, and that it is independent of the composition of the sample. In cases of interest, even though the bulk solution is dilute, for surface-enhanced species the interface might no longer be considered dilute. Therefore, the attenuation length depends on the chemical compounds involved in the experiment. We propose a modification to the attenuation coefficient to account for the attenuation due to the different chemical compounds where the integral in the exponential function becomes 

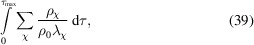

with λ_χ_ the attenuation length in a solution or solid made up of the pure compound χ, and ρ_χ_ the profile concentration of χ. This model is equivalent to a resistive model for the attenuation length 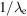 = 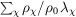 , and in the limit case of dilute solution it is equivalent to the model investigated in this work. A similar formulation was introduced in earlier works (Baschenko, 1991 ▸; Eschen *et al.*, 1995 ▸).

The influence of the total concentration profile is not investigated because in the forward model it plays a similar role as the attenuation length. However, since the model for the total concentration is a simplification of reality, its effect on profile reconstruction should also be the subject of further investigation.

## 6. Conclusions

Throughout this work, we have shown that it is possible to reconstruct absolute, quantitative concentration depth profiles for individual target chemical states in aqueous solutions via their XPS spectra. Hence, the concentration profiles of solution components can be inferred, paving the way for an improved understanding of mechanisms and processes at the liquid–air interface. We focused on reconstructing the depth profile in the depth range [−1.5, 1.5] nm and [−1, 1] nm using attenuation lengths either in the interval [1.62, 1.95] nm or [1.28, 5.5] nm, and showed that the wider interval is more favorable. The quality of the profiles strongly depends on both the quality of the model and the quality of the data. Improving the quality of the data is intrinsically limited by the physics of the experiments and the resources; however, the uncertainty of the measurement model can be considerably reduced by narrowing the uncertainty in the values of the parameters, in particular the values of the attenuation length. A relatively low measurement noise level is favorable for profile reconstruction, but it is not a critical issue because the inversion model is built upon the statistical description of the noise. Hence, for reasonably good signal quality, and assuming that the peak fitting and background removal are of reasonably good quality, improving the SNR is not of the highest priority. The knowledge of the attenuation lengths in the model is more critical than the SNR. However, the level of uncertainty investigated in this work shows that current knowledge of attenuation length should allow for depth profile reconstruction and that the quality and uncertainty of reconstruction are conditional to the attenuation length uncertainty. The number of probed attenuation lengths and their interval do matter for depth profile reconstruction and should be considered as critical parameters determining the accessible depth reconstructions. The wide attenuation length was chosen to showcase the possible gain for the inversion method when applied to reconstruction over the interval [−1.5, 1.5] nm.

Furthermore, the geometry factor *H* is built upon a smooth edge volume approximation of the sample. The approximation of the total concentration ρ_tot_ is chosen arbitrarily to represent the smooth transition between the solution bulk and the outside of the sample. Even though the parametric total concentration is plausible, it remains an approximation of reality that carries errors. Moreover, the transition length parameter Δ_*r*_ has to be defined for each sample, possibly from MD simulations, and the reconstruction is conditional to this value.

The *a posteriori* model (26) is sharp, however only if the parameters of the model are precisely known. We have shown that the MAP estimate of the profile is robust against uncertainty in the attenuation length values and that profiles of interest can be reconstructed, except if the variations are too sharp. Moreover, the marginalization over the measurement operator space is wider than the *a posteriori* model. Besides, the variation in the estimation shows that, despite the amplitude of covariance of the posterior models, the variability in the estimates does not show such variability due to the regularization. Therefore, we conclude that the inversion of XPS data with PROPHESY is possible; however, the reconstructions should be reported along with the uncertainty level in the attenuation length. Finally, for depth profiling XPS experiments, we advocate for more attenuation lengths to be probed and over a wider range.

## 7. Related literature

The following references, not cited in the main body of the paper, have been cited in the supporting information: Baek *et al.* (2015 ▸); Nicholls *et al.* (2012 ▸); Olivieri *et al.* (2015 ▸); Pereya *et al.* (2015 ▸); Siegbahn & Siegbahn (1973 ▸); Thiébaut (2002 ▸); Twomey (1963 ▸). 

## Supplementary Material

Technical details for reproducibility: algorithms, model discretization, mathematical assumptions, potential evolution of the model and other results. DOI: 10.1107/S1600577523006124/ok5094sup1.pdf

## Figures and Tables

**Table 1:** Regularization parameters: normalized standard deviation 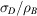 of the amplitude of the second order difference *D*ρ as defined in equation (27) of the supporting information

	ρ			
Sampling case	Eqn (32)	Eqn (33)	Eqn (34)	Eqn (35)
*N*_5_	1	10	1	1
*W*_5_	1	10	1	1
*W*_10_	1	10	1	1
