# Supplementary material for: Inversion model for extracting chemically resolved depth profiles across liquid interfaces of various configurations from XPS data: PROPHESY
Source: J Synchrotron Radiat. 2023 Aug 23;30(Pt 5):941–61. doi: 10.1107/S1600577523006124 (PMC10481271; doi:10.1107/S1600577523006124)
Supplement: Supplementary file 1 [file s-30-00941-sup1.pdf]

JOURNAL OF SYNCHROTRON RADIATION

Volume 30 (2023)

Supporting information for article:

Inversion model for extracting chemically resolved depth profiles across liquid interfaces of various configurations from XPS data: PROPHESY

Matthew Ozon, Konstantin Tumashevich, Jack J. Lin and Nønne L. Prisle

Supporting information: Inversion model for extracting chemically resolved depth profiles across liquid interfaces of various configurations from XPS data: PROPHESY

Matthew Ozon,* Konstantin Tumashevich, Jack J. Lin and

Nønne L. Prisle *

Center for Atmospheric Research, PO BOX 4500, University of Oulu, Finland.

E-mail: matthew.ozon@oulu.fi, nonne.prisle@oulu.fi

X-ray photoelectron spectroscopy, depth profile, inversion algorithm, aqueous surfactants, atmospheric surfaces, acquisition model

1. Assumption leading to the measurement model

In the work of Ottosson et al. 2010 (Ottosson *et al.*, 2010), the PE signal is defined as a function of electron kinetic energy, but it does not include the dependence of the parameters which is left implicit. However, in this work, we need this important information in order to understand where discrepancies may arise and where approximations may come from. In the work of Dupuy et al. 2021 (Dupuy *et al.*, 2021), the PE signal is modeled with integration over the volume of the sample of the product of the density ρ and the exponential attenuation. The beam profile is left out of the volume integral which implies some (left unknown) assumptions. However, the photon flux is a quantity that varies over the integration volume because the beam profile is not flat (Fedoseenko *et al.*, 2003; Kachel, 2016) Also, the amplitude of the flux is attenuated in the sample. Though, the attenuation of the photon flux can be neglected

over the thickness (less than 100 μm) of the sample, e.g. the linear attenuation length of 1 [keV] X-ray in water is around 2.5 mm (Berger, 1998). Therefore, neglecting the attenuation of the photon flux in the sample is a weak assumption for thin samples such as LJ. The variation in space of the differential cross-section density $\sigma_\chi(\nu, K_e, \theta)$ in the sample is also left out because the analyzer is sufficiently far from the sample (e.g. a few radii away) that the measurement angle is constant over the sample. In order to account for the spatial approximations in the photon flux, the alignment parameter α [m^{-2}] is introduced as a multiplicative factor (Ozon *et al.*, 2023; Dupuy *et al.*, 2021). The alignment parameter depends on the acquisition setup and should be estimated for every acquisition, e.g. from raw experimental data.

Our presented model accounts for the spatial and spectral variability of the model parameters, i.e. the photoionization differential cross-section density and the photon flux density. Hence, the electron flux J [electron s^{-1}] reaching the aperture of the measurement device (not including the device itself) is given by the summation of the contribution of all photon energy over the spectral domain Ω_ν, the contribution over the area where the photon beam extends on the sample, and the angle domain Ω_θ that cover the aperture of the kinetic analyzer. We write the photo-electron flux as it was modeled in (Ozon *et al.*, 2023)

$$J_\chi^k(K_e) = \int_{\Omega_V}\int_{\Omega_\nu}\int_{\Omega_\theta} \sigma_\chi(\nu, K_e, \theta) f_k(\nu, M)\rho(M) e^{-\int_0^{\tau_{\max}} \frac{\rho_{\text{tot}}(M_s(\tau))}{\rho_0 \lambda_e(K_e)}\mathrm{d}\tau} \mathrm{d}\theta \mathrm{d}\nu \mathrm{d}V \tag{1}$$

where $f_k(\nu, M)$ [photon m^{-2} eV^{-1}] is the photon spatial and spectral density for the central frequency ν_k, and ρ is the concentration expressed in number of molecules per unit volume [m^{-3}] and represents the concentration of an orbital, e.g. $\chi =$C1s. The concentration ρ_{tot} [m^{-3}] is the molecular concentration of all the species in the sample, e.g. $\rho_{\text{tot}} = \rho_{\text{water}} + \rho_{\text{SDS}}$ for an aqueous solution with SDS. The distance $\int_0^{\tau_{\max}} \frac{\rho_{\text{tot}}(M_s(\tau))}{\rho_0}\mathrm{d}\tau$ is the summation of the relative concentration $\frac{\rho_{\text{tot}}}{\rho_0}$ along the path of the emitted electrons from the emission point $M = (x_M, y_M, z_M)$ along the parametric

curve M_s leading to the surface of the liquid in direction of the analyzer located in $P = (x_0, y_0, z_0)$ at a distance $\tau_{\max} = \sqrt{(x_0 - x_M)^2 + (y_0 - y_M)^2 + (z_0 - z_M)^2}$ [m]. The emitted photoelectrons are assumed to be traveling in straight lines, and the attenuation length characterizes the losses by elastic or inelastic interaction with the sample. In this formulation, it is implicitly assumed that the scattering properties are uniform across the sample, and that outside the sample (where $\rho_{\text{tot}} = 0$) the photoelectrons do not undergo scattering events. The integration domain Ω_V covers the sample and dV is the infinitesimal volume around M. Considering any point M in the liquid and any point P outside of the liquid, the straight line joining M to P can be parameterized as:

$$M_s(s) = \begin{pmatrix} x(s) \\ y(s) \\ z(s) \end{pmatrix} = \begin{pmatrix} s \sin\omega \cos\beta + x_M \\ s \sin\beta + y_M \\ s \cos\omega \cos\beta + z_M \end{pmatrix} = \begin{pmatrix} s\frac{x_0 - x_M}{\tau_{\max}} + x_M \\ s\frac{y_0 - y_M}{\tau_{\max}} + y_M \\ s\frac{z_0 - z_M}{\tau_{\max}} + z_M \end{pmatrix} \tag{2}$$

where the direction angles ω and β are depicted in fig. (2) and s [m] is the parameter of the curve that represents the signed distance from point M. The angle ω is between the z-axis and the projection MP onto the plane zOx, and β is taken as the angle between the plane zOx and MP. Note that the concentrations ρ and ρ_{tot} in the model are not the same, one represents the density of all species ρ_{tot} (overwhelmingly water for aqueous solutions) and the other ρ is the concentration for a given target orbital in the sample. The spectral integration domain Ω_ν covers the support of the spectral density of the light source. The integration domain Ω_θ of the emission angle of the PE depends on the sample and the aperture of the kinetic energy analyzer.

In the case of synchrotron light sources, it is possible to assume that the light is monochromatic (Fedoseenko *et al.*, 2003; Kachel, 2016). The monochromaticity is measured relatively to the central frequency value ν_k as the ratio of the spread Δ_{ν_k} and ν_k. However, the spread Δ_{ν_k} should be compared to the spread of the kinetic energy of the emitted electrons because this is the signal of interest which should not

be blurred by the exciting light. We write $f_k(\nu, M) = F(\nu_k)\delta(\nu - \nu_k)f(M)$ with $F(\nu_k)$ the total photon flux [photon s^{-1}], $f(M)$ the profile density [m^{-2}], and δ the Dirac distribution modeling the spectral density [eV^{-1}], then the electron flux is

$$J_{\chi}^k(K_e) = F(\nu_k) \int_{\Omega_V} \int_{\Omega_\theta} \sigma_\chi(\nu_k, K_e, \theta) f(M) \rho(M) e^{-\int_0^{\tau_{\max}} \frac{\rho_{\text{tot}}(M_S(\tau))}{\rho_0 \lambda_e(K_e)} \mathrm{d}\tau} \mathrm{d}\theta \mathrm{d}V \qquad (3)$$

In the most general case, the angle θ, and its domain of integration Ω_θ, depend on the location in the sample, however, we approximate θ by that of the center of the sample and Ω_θ by the apparent angle of the aperture of the analyzer from the center of the sample. It was shown early on that the location of the sample relative to the analyzer (Siegbahn & Siegbahn, 1973) plays an important role in the measured signal. Also, the location in the sample, e.g. the relative angle of the surface and the solid angle of the aperture (Olivieri *et al.*, 2017), affects the amount of signal that can be measured. In the latter, it is shown that the integration domain should be the intersection between the illuminated sample volume with the observation cone. Rigorously, the signal reaching the aperture of the spectrometer is originating from the volume irradiated by the X-ray source convoluted with the area analyzed by the spectrometer (Guilet *et al.*, 2022). Note that the size of the photon beam also has an effect on the amount of signal coming from the vapor in LJ (Olivieri *et al.*, 2015).

From this assumption, we separate the two integrals

$$J_{\chi}^k(K_e) = F(\nu_k) \int_{\Omega_\theta} \sigma_\chi(\nu_k, K_e, \theta) \mathrm{d}\theta \int_{\Omega_V} f(M) \rho(M) e^{-\int_0^{\tau_{\max}} \frac{\rho_{\text{tot}}(M_S(\tau))}{\rho_0 \lambda_e(K_e)} \mathrm{d}\tau} \mathrm{d}V. \qquad (4)$$

Denoting the first integral as $\alpha_\theta \sigma_\chi(\nu_k, K_e)$, then we have

$$J_{\chi}^k(K_e) = \alpha_\theta F(\nu_k) \sigma_\chi(\nu_k, K_e) \int_{\Omega_V} f(M) \rho(M) e^{-\int_0^{\tau_{\max}} \frac{\rho_{\text{tot}}(M_S(\tau))}{\rho_0 \lambda_e(K_e)} \mathrm{d}\tau} \mathrm{d}V. \qquad (5)$$

Finally, we use the alignment parameter α [m^{-2}] an average probability density of interaction between the photon beam and the sample (Ozon *et al.*, 2023) to account for approximation of the photon beam profile f by a flat profile with effective photon

density $\alpha F(\nu_k)$ and the limited angular opening of the analyzer α_θ we have

$$J_\chi^k(K_e) = \alpha F(\nu_k)\sigma_\chi(\nu_k, K_e) \int_{\Omega_V} \rho(M) e^{-\int_0^{\tau_{\max}} \frac{\rho_{\text{tot}}(M_s(\tau))}{\rho_0 \lambda_e(K_e)} \mathrm{d}\tau} \mathrm{d}V. \tag{6}$$

For the sake of clarity, the volume integral in (6), which bears the geometry information, will be denoted $H(\rho, \lambda_e)$.

2. Discretization

We estimate the different cross-section densities $\tilde{\sigma}_\chi^{m,k}$ and the profile concentration ρ from a very limited number of acquisitions. Typically, the number of frequencies ν_k used for probing the sample is no more than $K = 5$. Ideally, we should have access to sufficiently many ($K = 20$) frequencies so that the collection of attenuation lengths $(\lambda_k)_{1 \leqslant k \leqslant K}$ would finely cover the range $[0, Z_{\max}]$ with $Z_{\max}$ [m] the maximum depth at which we want to reconstruct the concentration profile.

2.1. Depth and kinetic energy

To limit the challenge, we do not seek solutions in an infinite space of functions, but rather in a finite subspace. We approximate the sought functions by

$$\rho(r) = \sum_{n=1}^{N} \rho_n e_n(r) \qquad \text{and} \qquad \tilde{\sigma}_\chi^{m,k}(K_e) = \sum_{\ell=1}^{L} \sigma_\ell^{m,k} f_\ell(K_e) \tag{7}$$

with $(e_n)_{1 \leqslant n \leqslant N}$ and $(f_\ell)_{1 \leqslant \ell \leqslant L}$ two basis of the linear interpolation function subspace. The coefficient $(\rho_n)_{1 \leqslant n \leqslant N}$ and $(\sigma_\ell^{m,k})_{1 \leqslant \ell \leqslant L}$ are the values of the functions ρ and $\tilde{\sigma}_\chi^{m,k}$ evaluated at the discretization nodes of their respective domain $(r_n)_{1 \leqslant n \leqslant N}$ and $(K_{e_\ell}^k)_{1 \leqslant \ell \leqslant L}$. Using these approximations in the peak model eq. (23), we obtain

the approximation:

$$\begin{aligned} I_k^m &= \alpha_k F(\nu_k)\sigma_\chi(\nu_k)\int_{\Omega_{K_e}^{k,\chi}}\varphi_\ell^k(K_e)\sum_{\ell=1}^{L}\sigma_\ell^{m,k} f_\ell(K_e)H(\rho,\lambda_e(K_e))\mathrm{d}K_e + \varepsilon^{m,k} \\ &= \alpha_k F(\nu_k)\sigma_\chi(\nu_k)\sum_{\ell=1}^{L}\sigma_\ell^{m,k}\sum_{n=1}^{N}H_{\ell,n}^k\rho_n + \varepsilon^{m,k} \\ &= \alpha_k F(\nu_k)\sigma_\chi(\nu_k)(\sigma^{m,k})^t H^k\rho + \varepsilon^{m,k} \end{aligned} \tag{8}$$

where $\sigma^{m,k} = [\sigma_1^{m,k}\,\sigma_2^{m,k}\,\ldots\,\sigma_L^{m,k}]^t$ and $\rho = [\rho_1\,\rho_2\,\ldots\,\rho_N]^t$. The discretization noise $\varepsilon^{m,k}$ is the error due to the approximation of the functions ρ and $\tilde{\sigma}_\chi^{m,k}$ by their piecewise linear approximations. The matrix element for the kth frequency is:

$$H_{\ell,n}^k = \int_{\Omega_{K_e}^{k,\chi}}\varphi_\ell^k(K_e)f_\ell(K_e)H(e_n,\lambda_e(K_e))\mathrm{d}K_e. \tag{9}$$

Note that $H_{\ell,n}^k$ does not depend on the specific peak m, rather it is a sample-geometry factor.

2.2. Attenuation length

In practice, for the measurement of a single spectrum (collection of PE signals centered around a reference kinetic energy, K_k e.g. $K_k = \frac{K_{e_1}^k + K_{e_L}^k}{2}$), the variation in attenuation length λ_e is rather small (Thürmer *et al.*, 2013; Ottosson *et al.*, 2010) compared with the central value. For instance, for $\lambda_e(K_k) = 2$ [nm] the variation is of the order of 10^{-2} [nm] over a range of kinetic energy of a few eV. Therefore, we assume that it can be approximated by

$$\lambda_e(K_e) = \lambda_k + \frac{\partial\lambda_e}{\partial K_e}(K_k)(K_e - K_k) + O\left((K_e - K_k)^2\right) \tag{10}$$

with $\lambda_k = \lambda_e(K_k)$. The Landau notation $O\left((K_e - K_k)^2\right)$ stands for the higher order terms, i.e. all the terms of order higher than or equal to 2 in this case. Substituting SI(10) in the model SI(9), we obtain

$$\begin{aligned} H(e_n,\lambda_e(K_e)) &= H(e_n,\lambda_k) \\ &\quad + \frac{\partial\lambda_e}{\partial K_e}(K_k)\frac{K_e - K_k}{\lambda_k^2}H(e_n d_P,\lambda_k) + O\left((K_e - K_k)^2\right) \end{aligned} \tag{11}$$

then, provided that $\frac{\partial \lambda_e}{\partial K_e}(K_k)\frac{K_e-K_k}{\lambda_k^2}H(e_n d_P,\lambda_0) \ll H(e_n,\lambda_k)$, we have

$$H(e_n,\lambda_e(K_e)) = H(e_n,\lambda_k) + \iota_n^k \tag{12}$$

where ι_n^k stands for the approximation error. The condition holds for the integration domain, i.e. the support of the cross-section density $\sigma_\chi(\nu_k, K_e)$, whose length ΔK_e ($\Delta K_e \leqslant |K_{e_L} - K_{e_1}| \lessapprox 5$ [eV]) is much smaller than the range over which the variation in the attenuation length λ_e are observable, $\simeq 100$ [eV]. The matrix element simplifies further and becomes

$$\begin{aligned} H_{\ell,n}^k &= H(e_n,\lambda_k)\int_{\Omega_{K_e}^{k,\chi}} \varphi_\ell^k(K_e) f_\ell(K_e)\mathrm{d}K_e + \iota_{\ell,n}^k \\ &= T_k c_k H(e_n,\lambda_k) + \iota_{\ell,n}^k. \end{aligned} \tag{13}$$

which is in essence the outer product of a discretization matrix over the kinetic energy space and that over the depth space. Therefore, the rank of H^k is 1, making it impossible to recover depth information from one spectrum. The coefficient $T_k c_k$ is the discretization of the kernel function φ_ℓ^k onto the basis functions $(f_\ell)_{1\leqslant\ell\leqslant L}$, and does not depend on the index ℓ because the kernel and basis functions have the same form for all index ℓ. From here, eq. SI(8) becomes

$$I_k^m = \alpha_k T_k c_k F(\nu_k)\sigma_\chi(\nu_k) p^{m,k}[H(e_1,\lambda_k),\, H(e_2,\lambda_k)\, \ldots\, H(e_N,\lambda_k)]\rho + \varepsilon^{m,k} \tag{14}$$

where $\varepsilon^{m,k}$ now also includes the approximation errors described by $(\iota_{\ell,n}^k)_{1\leqslant\ell\leqslant L}$.

3. Photoionization cross-section density estimation

We assume that the background of the spectra have been estimated and removed from the spectral data, for instance using the package SPANCF (Kukk *et al.*, 2001; Kukk *et al.*, 2005) or any other algorithm for background removal (Baek *et al.*, 2015). We propose an alternative to current C1s peak fitting methods (Kukk *et al.*, 2001; Kukk *et al.*, 2005; Major *et al.*, 2020) that does not rely on parametric peaks to be fitted.

This is a model-free method whose output is the probability density $\tilde{\sigma}^k_{\mathrm{C1s}}$ and the area I_k, i.e. the total PE count from the signal of interest. The smooth output $\tilde{\sigma}^k_{\mathrm{C1s}}$ is denoised and can be used as input for a regular peak fitting routine, e.g. SPANCF (Kukk *et al.*, 2001; Kukk *et al.*, 2005). The method is written in terms of C1s, but is not limited to this orbital or element.

For each kinetic energy in the kth spectrum, by combining eq. 16, eq. 22 and eq. SI(12), we have

$$I(\nu_k, K^k_{e_\ell}) = \alpha_k T_k \sigma_{T_k} F(\nu_k)\sigma_{\mathrm{C1s}}(\nu_k)\left(H(\rho,\lambda_k) + \sum_{n=1}^{N}\rho_n \iota^k_n\right)\tilde{\sigma}^k_{\mathrm{C1s}}(K^k_{e_\ell}) \quad (15)$$

By definition, adding up the contribution of each kinetic energy leads to the total count I_k:

$$I_k = \alpha_k T_k F(\nu_k)\frac{\sigma_{T_k}}{\delta_{K_e}}\sigma_{\mathrm{C1s}}(\nu_k)\left(H(\rho,\lambda_k) + \sum_{n=1}^{N}\rho_n \iota^k_n\right)\sum_{\ell=1}^{L}\delta_{K_e}\tilde{\sigma}^k_{\mathrm{C1s}}(K^k_{e_\ell}) \quad (16)$$

where the discretization step δ_{K_e} [eV] is assumed constant. The discrete sum over ℓ is identified as the Riemann quadrature, hence $1 = \sum_{\ell=1}^{L}\delta_{K_e}\tilde{\sigma}^k_{\mathrm{C1s}}(K^k_{e_\ell}) + O(\delta^2_{K_e})$. By construction, the approximation error is at most linear in the kinetic energy step, $\iota^k_n = O\left(\delta_{K_e}\right)$, and from the definition we have $\delta_{K_e} \simeq \sigma_{T_k}$ and $\delta_{K_e} \ll \Delta_{K_e}$. Hence, the total count becomes:

$$I_k = \alpha_k T_k F(\nu_k)\frac{\sigma_{T_k}}{\delta_{K_e}}\sigma_{\mathrm{C1s}}(\nu_k) + O(\delta_{K_e}). \quad (17)$$

Therefore, the spectrum acquisition model can be simplified using the total count I_k so that

$$I(\nu_k, K^k_{e_\ell}) = I_k\delta_{K_e}\tilde{\sigma}^k_{\mathrm{C1s}}(K^k_{e_\ell}) + O(\delta^2_{K_e}) + \varepsilon^k_\ell \quad (18)$$

We assume that the discretization error are negligible compared with the noise level, and that the measurement noise terms $\varepsilon^k_\ell \sim \mathcal{N}(0,(\sigma^k_\ell)^2)$ are mutually independent. The total count I_k and its variance σ^2_k can then be estimated from the measurements

$$I_k = \sum_{\ell=1}^{L} I(\nu_k, K^k_{e_\ell}) \quad \text{and} \quad \sigma^2_k = \sum_{\ell=1}^{L}(\sigma^k_\ell)^2 = \sum_{\ell=1}^{L} I(\nu_k, K^k_{e_\ell}) + I_{\mathrm{bg}}(\nu_k, K^k_{e_\ell}). \quad (19)$$

From here, the coefficients of the photoionization cross-section density can be estimated by solving:

$$\begin{bmatrix} I(\nu_k, K_{e_1}^k) \\ I(\nu_k, K_{e_2}^k) \\ \vdots \\ I(\nu_k, K_{e_L}^k) \\ 1 \\ 0 \\ 0 \\ \vdots \\ 0 \end{bmatrix} = \begin{bmatrix} I_k \delta_{K_e} \mathbb{I}_L \\ \begin{bmatrix} \delta_1 & \delta_2 & \dots & \delta_L \end{bmatrix} \\ D \end{bmatrix} \begin{bmatrix} \tilde{\sigma}_{\mathrm{C1s}}^k(K_{e_1}^k) \\ \tilde{\sigma}_{\mathrm{C1s}}^k(K_{e_2}^k) \\ \vdots \\ \tilde{\sigma}_{\mathrm{C1s}}^k(K_{e_L}^k) \end{bmatrix} + \begin{bmatrix} \varepsilon_1^k \\ \varepsilon_2^k \\ \dots \\ \varepsilon_L^k \\ \iota_c \\ \varepsilon_{D_1} \\ \varepsilon_{D_2} \\ \dots \\ \varepsilon_{D_{L-n_d}} \end{bmatrix} \tag{20}$$

with $\mathbb{I}_L$ the L-order identity matrix. We write the more compact form:

$$\mathbf{y} = R_k^{\mathrm{reg}} \tilde{\sigma}_{\mathrm{C1s}}^k + \varepsilon, \qquad \varepsilon \sim \mathcal{N}(0, \Gamma_I). \tag{21}$$

We assume that all the perturbation elements in ε are Gaussian distributed with covariance matrix Γ_I that is block diagonal. The first block is diagonal with entries $(\sigma_\ell^k)^2$. The second block is the variance of ι_c should reflect the Riemann integration error amplitude. The last block corresponding to the terms $(\varepsilon_{D_i})_{1 \leqslant i \leqslant L-2}$ bears the smoothness strength information. The operator D is the second order difference operator in dimension L, which regularizes the inversion by seeking somewhat smooth solutions. Additionally, the total photoionization cross-section is a positive number and so is its density function σ_{C1s}. The positivity constraint can be enforced in the optimization algorithm used for seeking the solution of the optimization problem

$$\hat{\sigma}_{\mathrm{C1s}}^k | I_k, \mathbf{y} \in \underset{\tilde{\sigma}_{\mathrm{C1s}}^k \geqslant 0}{\arg\min} \{ \| \mathbf{y} - R_k^{\mathrm{reg}} \tilde{\sigma}_{\mathrm{C1s}}^k \|_{\Gamma_I}^2 \} \tag{22}$$

An algorithm such as VMLM-B (Thiébaut, 2002) can solve the optimization problem SI(22). The a posteriori probability density $\mathsf{P}(\tilde{\sigma}_{\mathrm{C1s}}^k | I_k, \mathbf{y})$ is modelled as the product of three Gaussian functions, one for the PE measurements (likelihood) and two for the a priori (smoothness and integral value). Despite being formed as the product of Gaussian function, the resulting a posteriori is not a Gaussian function. Therefore, one

can not conclude that the maximum of the posterior $\hat{\sigma}^k_{\mathrm{C1s}}|I_k, \mathbf{y}$ is also its expectation. Estimating the uncertainty in $\hat{\sigma}^k_{\mathrm{C1s}}|I_k, \mathbf{y}$ due to the uncertainty in the model, i.e. the variability of the value I_k, may be formulated as the covariance of the estimation

$$\mu_{\hat{\sigma}_{\mathrm{C1s}}|\mathbf{y}} = \int_{\Omega_{I_k}} \hat{\sigma}_{\mathrm{C1s}}|I_k, \mathbf{y}\mathsf{P}(I_k)\mathrm{d}I_k \tag{23}$$

$$\Gamma_{\hat{\sigma}_{\mathrm{C1s}}|\mathbf{y}} = \int_{\Omega_{I_k}} (\hat{\sigma}_{\mathrm{C1s}}|I_k, \mathbf{y} - \mu_{\hat{\sigma}_{\mathrm{C1s}}|\mathbf{y}})(\hat{\sigma}_{\mathrm{C1s}}|I_k, \mathbf{y} - \mu_{\hat{\sigma}_{\mathrm{C1s}}|\mathbf{y}})^t\mathsf{P}(I_k)\mathrm{d}I_k$$

these quantities can be computed by sampling the model, e.g. $I_k \sim \mathcal{N}(\bar{I}_k, \sigma_k^2)$. An example of estimation of $\hat{\sigma}^k_{\mathrm{C1s}}|I_k, \mathbf{y}$ for 5 photon energies is shown in fig. SI1. The square root of the variability diagonal entries is depicted as the shaded area. Note that due to the low dimensionality of the model (1D) and the speed for solving such a problem, it is possible to use simple sampling methods since a small number of values of I_k would be enough to obtain an estimation of the distribution of $\hat{\sigma}_{\mathrm{C1s}}|I_k, \mathbf{y}$.

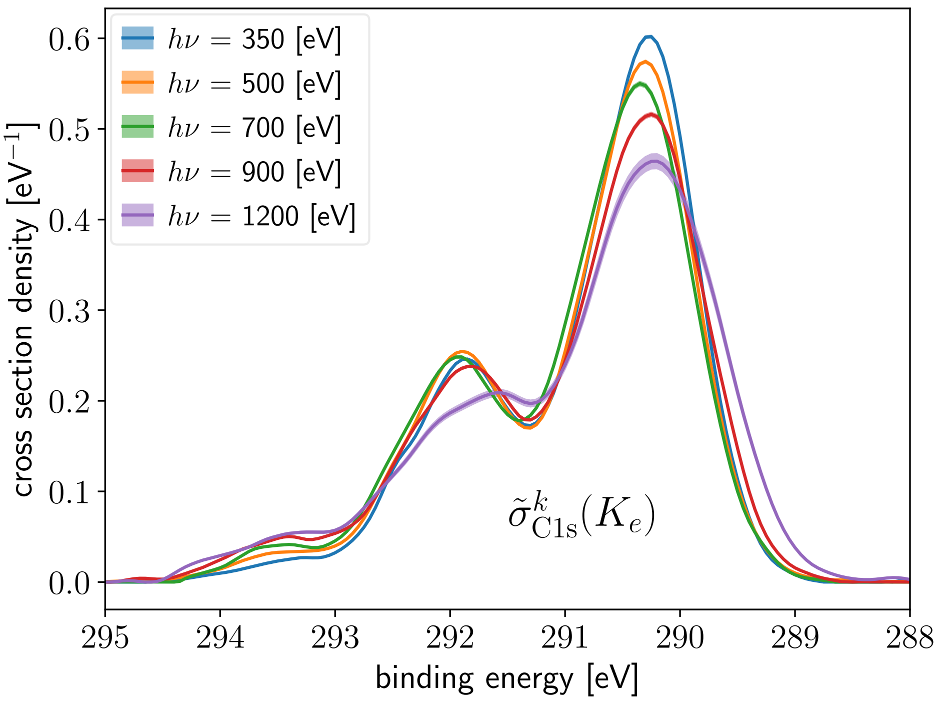

Fig. 1. Examples of the photoionization cross-section density estimations from the PE spectra as described in sec. SI3 for a sample probed with five photon energies. For each photon energy, the estimate and the uncertainty are depicted as solid lines and shaded areas respectively.

4. Detail of the optimization problem eq. (26)

The optimization problem defined in eq. (26) of the paper relies on two probability densities, i.e. the measurement *likelihood* $\mathsf{P}(\mathbf{y}|A^m, \rho)$ and the *a priori* $\mathsf{P}(A^m, \rho)$. Here we detail the meaning and definition of each term. As stated in sec. 3 of the paper, the measurement noise is approximated by a Gaussian distribution because the number of counts is assumed to be greater than 30, making the Gaussian approximation to the Poisson distribution acceptable. We chose this approximation to simplify the implementation of the optimization algorithm, however, it is not a critical assumption and can be modified in a straightforward manner. Here, we have the approximated

likelihood

$$\mathsf{P}(\mathbf{y}|A^m,\rho) = \frac{1}{\sqrt{(2\pi)^K \det\Gamma}} e^{-(\mathbf{y}-A^m\rho)^t\Gamma^{-1}(\mathbf{y}-A^m\rho)}. \tag{24}$$

Additionally, the noise is assumed to be independent because each measurement is acquired from different experimental setups (e.g. different photon energy) and different samples (the piece of the sample used for acquisition is not the same for different photon energy even though the bulk solution is the same). Therefore, the covariance matrix Γ is diagonal with entries $\left(\frac{\sigma_k}{\alpha_k T_k F(\nu_k)\sigma_\chi(\nu_k)}\right)^2$ where the variance $(\sigma_k)^2$ is defined in eq. SI(19). Note that accounting for the Poisson distributed noise implies changing $\mathsf{P}(\mathbf{y}|A^m,\rho)$ in eq. SI(24), with expected values I_k (see eq. SI(19)). Maximizing the likelihood is an under-determined problem because an infinity of solutions ρ lead to $\mathbf{y} = A^m\rho$, hence, we need to constrain the space of possible solutions. The process of constraining the possible solutions is known as regularizing and often relies on *a priori* (Leong *et al.*, 2023). The second probability density in the optimization problem is the *a priori* $\mathsf{P}(A^m,\rho)$, which reflects the knowledge of the state ρ without data, and the uncertainty in the measurement operator. The state ρ and the measurement operator A^m are stochastically independent, hence $\mathsf{P}(A^m,\rho) = \mathsf{P}(A^m)\mathsf{P}(\rho)$. The state *a priori* $\mathsf{P}(\rho)$ represents the probability density of the state ρ. It is interpreted as the plausibility of concentration profiles. This term does not involve nor requires the knowledge of the ground truth. Instead, it represents the properties we expect from a concentration profile. Here, we assume that ρ is not chaotic and is rather smoothly varying with depth (human bias). This is reasonable at the scale/granularity the sample is observed, i.e. averaged over the dimension other than the depth. To represent mathematically this assumption, we resort to the second order difference operator

$$D = \begin{bmatrix} 1 & -2 & 1 & 0 & 0 & \dots & 0 \\ 0 & 1 & -2 & 1 & 0 & \dots & 0 \\ \ddots & \ddots & \ddots & \ddots & \ddots & & \\ 0 & \dots & 0 & 1 & -2 & 1 & 0 \\ 0 & \dots & 0 & 0 & 1 & -2 & 1 \end{bmatrix} \tag{25}$$

an $(N-2)\times N$ matrix which is closely related to the second-order derivative. The choice of difference operator for regularizing optimization problem is ubiquitous and well established in inverse problem (Stolzenburg *et al.*, 2022; Nicholls *et al.*, 2012; Rudin *et al.*, 1992; Twomey, 1963). From here, we write

$$\mathsf{P}(\rho) = \frac{1}{\sqrt{(2\pi)^{N-2}\det\Gamma_D}} e^{-\frac{1}{2}(\mathbf{y}_D - D\rho)^t \Gamma_D^{-1}(\mathbf{y}_D - D\rho)} \tag{26}$$

with $\mathbf{y}_D$ the $(N-2)$-vector of expected values of second order differences, and Γ_D its covariance matrix. Numerically, we choose $\mathbf{y}_D$ as the vector whose entries are all 0, which implies that the expected profile is linear (at least piecewise linear). The covariance matrix plays the role of the moderator of the linear-profile assumption. The diagonal of Γ_D expresses by how much the profile can deviate from linearity, and the off-diagonal elements represent the correlation between the values of the second-order differences at different depth; it is a control over the smoothness of the second-order differences. Formally, the entries of Γ_D are

$$\Gamma_{D_{i,j}} = \frac{N}{K}\sigma_D^2 e^{-\frac{(r_i - r_j)^2}{2\delta_D^2}}, \qquad 1 \leqslant i,j \leqslant N-2 \tag{27}$$

where σ_D [m^{-3}] controls the amplitude of the second order difference, the correlation length δ_D [nm] controls its smoothness, and the ratio $\frac{N}{K}$ ensures scalability. The values for σ_D and δ_D are semi-arbitrary, they are chosen in the ballpark of acceptable amplitude of the second order difference and arbitrarily determined so that the reconstruction is acceptable. The choice for σ_D could be automated with a criteria such as the L-curve (Stolzenburg *et al.*, 2022).

For the optimization problem eq. (26), the probability density $P(A^m)$ does not play role directly because the optimization is against the concentration profile ρ. However, for the quantification of uncertainty in the reconstruction due to the uncertainty in the model, this probability is central. From the peak area model eq. SI(14), the

measurement probability density can be written as

$$\begin{aligned}\mathsf{P}(A^m) &= \mathsf{P}((\tau_k)_{1\leqslant k\leqslant K},(\lambda_k)_{1\leqslant k\leqslant K},\rho_{\text{tot}})\\ &= \mathsf{P}((\lambda_k)_{1\leqslant k\leqslant K}|\rho_{\text{tot}},(\tau_k)_{1\leqslant k\leqslant K})\mathsf{P}(\rho_{\text{tot}},(\tau_k)_{1\leqslant k\leqslant K})\end{aligned} \tag{28}$$

where $\tau_k = (\alpha_k, T_k, c_k, F(\nu_k), \sigma_\xi(\nu_k), p^{m,k})$ gathers all the multiplicative terms of the measurement model. Here, we are interested only in the uncertainty associated with the attenuation lengths $(\lambda_k)_{1\leqslant k\leqslant K}$. As a working assumption, we state that the uncertainties in the attenuation length and those of τ_k and ρ_{tot} are stochastically independent, implying $\mathsf{P}((\lambda_k)_{1\leqslant k\leqslant K}|\rho_{\text{tot}},(\tau_k)_{1\leqslant k\leqslant K}) = \mathsf{P}((\lambda_k)_{1\leqslant k\leqslant K})$. Further, we assume that τ_k and ρ_{tot} are perfectly know, therefore, the probability density of the measurement operator reduces to that of the attenuation lengths $\mathsf{P}(A^m) = \mathsf{P}((\lambda_k)_{1\leqslant k\leqslant K})$. We are interested in the consequences of the deviation of the available attenuation lengths values relative to the true values $(\lambda_k^0)_{1\leqslant k\leqslant K}$, therefore, we choose to write the probability density conditionally to the true values

$$\mathsf{P}(A^m) = \mathsf{P}((\lambda_k)_{1\leqslant k\leqslant K}|(\lambda_k^0)_{1\leqslant k\leqslant K})\mathsf{P}((\lambda_k^0)_{1\leqslant k\leqslant K}). \tag{29}$$

The term $\mathsf{P}((\lambda_k^0)_{1\leqslant k\leqslant K})$ is an *a priori* term that is uninformative for this work, so, we focus on the conditional $\mathsf{P}((\lambda_k)_{1\leqslant k\leqslant K}|(\lambda_k^0)_{1\leqslant k\leqslant K})$. We consider two possible probability densities for describing the uncertainties: 1) independent errors, and 2) global error. The independent errors represent the errors that the granularity of the current models cannot represent. These error are small variations of the order of a few percents (2.5%). On the other hand, the global error reflects uncertainties that could result from a shift in a fit, or differences between two models. These errors are plausibly of the order of 25%. For the independent error, we write for each attenuation length

$$\lambda_k = (1+\kappa_k)\lambda_k^0, \qquad \text{with} \qquad \kappa_k \sim \mathcal{U}([-\tau_\lambda,\tau_\lambda]) \tag{30}$$

where τ_λ is the relative uncertainty level, and $(\kappa_k)_{1\leqslant k\leqslant K}$ are independent uniformly distributed random variables. The global error is also modeled with a relative error term, however, it the same value applies across all attenuation lengths

$$[\lambda_1,\, \lambda_2 \,\ldots\, \lambda_K] = (1+\kappa)[\lambda_1^0,\, \lambda_2^0 \,\ldots\, \lambda_K^0], \quad \text{with} \quad \kappa \sim \mathcal{U}([-\tau_\lambda, \tau_\lambda]). \tag{31}$$

A more refined sampling model may be used for investigating the effect of EAL uncertainty. For instance, the parameters of the following semi-empirical attenuation length model from the work of (Emfietzoglou & Nikjoo, 2007) could be sampled:

$$\lambda_k = \frac{K_k}{A \ln K_k + B + C\frac{1}{K_k}}. \tag{32}$$

The parameters A, B and C are fitted from experimental datasets, e.g. IXS-D2, therefore, their accuracy is limited. This formula is an approximation of the attenuation length variation with respect to the kinetic energy and does not capture the fine variations of the EAL. Furthermore, the model parameters depends both on the dataset used for fitting as well as the fitting algorithm. Therefore, the parameters A, B, and C bear uncertainty. Using this model, the attenuation length uncertainty can be represented with

$$\mathsf{P}(\lambda_k|\lambda_k^0) = \mathsf{P}(A|A^0)\mathsf{P}(B|B^0)\mathsf{P}(C|C^0) \tag{33}$$

where the probability distribution for each parameter may be modeled as a uniform distribution centered on the most likely value of the parameter, e.g. A^0, B^0, and C^0. The attenuation length error investigated in section 4.2.2 is equivalent to studying the effect of the parameter B in eq. SI(32) with $A = 0$ and $C = 0$.

Finally, the data probability $\mathsf{P}(y)$ is not necessary to compute the MAP estimate. Actually, in most practical cases, this probability is intractable. We choose to focus on the noise and write

$$\mathsf{P}(\mathbf{y}) = \mathsf{P}(\mathbf{y}|\mathbf{y}_0)\mathsf{P}(\mathbf{y}_0) \tag{34}$$

where $\mathsf{P}(\mathbf{y}|\mathbf{y}_0)$ is the noise distribution given the non-noisy observation $\mathbf{y}_0$

$$\mathsf{P}(\mathbf{y}|\mathbf{y}_0) = \frac{1}{\sqrt{(2\pi)^K \det \Gamma}} e^{-(\mathbf{y}-\mathbf{y}_0)^t \Gamma^{-1} (\mathbf{y}-\mathbf{y}_0)}. \tag{35}$$

For the noise marginalization, we use the above with $\mathbf{y}_0 = A^m \rho_{\mathrm{GT}}$.

5. Truncated peak area model

Because the optimization problem eq. (26) is not numerically advantageous separate/truncate the known from the unknown values of the concentration profile. The augmented model is

$$\begin{bmatrix} \mathbf{y}^m \\ \mathbf{y}_D \end{bmatrix} = \begin{bmatrix} A^m \\ D \end{bmatrix} \rho + \begin{bmatrix} \varepsilon^m \\ \varepsilon_D \end{bmatrix} = \bar{A}^m \rho + \bar{\varepsilon}^m \tag{36}$$

where ε_D is also a zero-mean Gaussian random vector which bears the meaning of tolerance to deviation from the expected values $\mathbf{y}_D$. The covariance matrix Γ_D represents the strength of the a priori.

The estimation in this form is unstable because we need to discretize the geometry factor H fairly deep in order to capture most of the signal, e.g. 20 nm for a maximum penetration depth of 5 nm. However, the signal is only informative over the first layers of the surface, at most 5 nm. Hence, instead of solving for all the entries in ρ, we will focus a the subset $\rho_S = [\rho_{N_b+1}, \rho_{N_b+2}, \dots \rho_{N_S-1}]^t$ corresponding to the first layers, and set the entries for the deeper layers to the bulk concentration ρ_B, hence the concentration vector can be written as $\rho = [\rho_1, \dots \rho_1, \rho_S^t, \rho_B, \dots \rho_B]^t$. Furthermore, the first component is assumed to be known, $\rho_1 = 0$. By reorganizing and truncating eq. SI(36), we finally obtain:

$$\mathbf{y}_S^m = \begin{bmatrix} \mathbf{y}^m \\ \mathbf{y}_{D_S} \end{bmatrix} - \begin{bmatrix} A_b^m \\ D_b \end{bmatrix} \begin{bmatrix} \rho_1 \\ \vdots \\ \rho_1 \end{bmatrix} - \begin{bmatrix} A_B^m \\ D_B \end{bmatrix} \begin{bmatrix} \rho_B \\ \vdots \\ \rho_B \end{bmatrix} = \begin{bmatrix} A_S^m \\ D_S \end{bmatrix} \rho_S + \begin{bmatrix} \varepsilon_S^m \\ \varepsilon_{D_S} \end{bmatrix} = \bar{A}_S^m \rho_S + \bar{\varepsilon}_S^m \tag{37}$$

where the truncated matrices are

$$A_b^m = \begin{bmatrix} A_{1,1}^m & \dots & A_{1,N_b}^m \\ A_{2,1}^m & \dots & A_{2,N_b}^m \\ \vdots & & \vdots \\ A_{K,1}^m & \dots & A_{K,N_b}^m \end{bmatrix}, A_S^m = \begin{bmatrix} A_{1,N_b+1}^m & \dots & A_{1,N_S-1}^m \\ A_{2,N_b+1}^m & \dots & A_{2,N_S-1}^m \\ \vdots & & \vdots \\ A_{K,N_b+1}^m & \dots & A_{K,N_S-1}^m \end{bmatrix}, A_B^m = \begin{bmatrix} A_{1,N_S}^m & \dots & A_{1,N}^m \\ A_{2,N_S}^m & \dots & A_{2,N}^m \\ \vdots & & \vdots \\ A_{K,N_S}^m & \dots & A_{K,N}^m \end{bmatrix} \tag{38}$$

and

$$D_b = \begin{bmatrix} D_{1,1} & \dots & D_{1,N_b} \\ D_{2,1} & \dots & D_{2,N_b} \\ \vdots & & \vdots \\ D_{K,1} & \dots & D_{K,N_b} \end{bmatrix}, D_S = \begin{bmatrix} D_{1,N_b+1} & \dots & D_{1,N_S-1} \\ D_{2,N_b+1} & \dots & D_{2,N_S-1} \\ \vdots & & \vdots \\ D_{K,N_b+1} & \dots & D_{K,N_S-1} \end{bmatrix}, D_B = \begin{bmatrix} D_{1,N_S} & \dots & D_{1,N} \\ D_{2,N_S} & \dots & D_{2,N} \\ \vdots & & \vdots \\ D_{K,N_S} & \dots & D_{K,N} \end{bmatrix} \tag{39}$$

The data vector $\mathbf{y}_S^m$ is different in size from the data vector of eq. SI(36) since only the subset of rows mapping ρ_S in the difference operator D are retained. Now, the values of the data vector are corrected, the covariance too must be adjusted. For the sake of example, we will only account for the uncertainty in the presumed known concentration values and leave the measurement uncertainty aside. Assuming that both ρ_1 and ρ_B are random variables with variance σ_B^2, the covariance matrix Γ_S^m is then given by:

$$\Gamma_S^m = \begin{bmatrix} \tilde{\Gamma} & 0 \\ 0 & \Gamma_{D_S} \end{bmatrix} + \sigma_B^2 \begin{bmatrix} A_b^m & A_B^m \\ D_b & D_B \end{bmatrix} \begin{bmatrix} A_b^m & A_B^m \\ D_b & D_B \end{bmatrix}^T \tag{40}$$

Note that accounting for the model uncertainties in the covariance model is not required since the optimization problem eq. (26) assumes the model to be known.

6. Algorithms

6.1. Algorithm for computing the inversion

For solving the optimization problem eq. (26) for the formulation described in sec. SI5, we turn to a primal-dual algorithm described in the paper Chambolle & Pock (2011) because of its convergence properties, simplicity for implementing the positivity constraint and the simplicity of implementation. The algorithm described

in algorithm SI6.1 – dubbed ALG2 in the paper Chambolle & Pock (2011) – relies on a primal-dual reformulation of the optimization problem and uses the proximal operator to iteratively converge to an optimal point. The proximal operators can be understood as projections onto convex sets. In the algorithm ALG2, the formulation alternate between projections in the primal space and dual space. In the reformulation 28, the projection in the dual space makes the iterates evolve toward the unconstrained solution, and the projection in the primal space enforces the positivity constraint. Other formulations are possible, there is no unique solution to solve such problem, but the proposed one is sufficiently efficient and intuitive.

Algorithm SI6.1: Statement of Algorithm 2 described by Chambolle & Pock (2011).

Require: $\gamma, \tau^0, \sigma^0 > 0$ with $\tau^0\sigma^0 L_A^2 \leqslant 1, x^0 \in \Omega_\rho, s^0 \in \Omega_{\mathbf{y}}, \bar{x}^0 = x^0$, $N_{\max} \in \mathbb{N}$, $r_y \in [0,1]$, $r_x \in [0,1]$ and $\Gamma_w \in \mathbf{S}_{++}^K$

while $\bar{B}_1 + \bar{B}_2 + \bar{B}_3$ **do**

Dual step

$s^{t+1} = \text{prox}_{\sigma^t F^\star}(s^t + \sigma^t A\bar{x}^t)$

Primal step

$x^{t+1} = \text{prox}_{\tau^t G}(x^t - \tau^t A^T s^{t+1})$

Update hyper-parameters

$\theta^t = \frac{1}{\sqrt{1+2\gamma\tau^t}}$, $\tau^{t+1} = \theta^t\tau^t$, and $\sigma^{t+1} = \frac{\sigma^t}{\theta^t}$

$\bar{x}^{t+1} = x^{t+1} + \theta^t(x^{t+1} - x^t)$

Stopping criterion

$B_1 = \left(\underset{k}{\text{median}}\{\frac{|y_k^m - (Ax^{t+1})_k|}{y_k^m}\} \leqslant r_y\right)$

$B_2 = \left(\frac{\|x^{t+1} - x^t\|_{\Gamma_w}}{\|x^{t+1}\|_{\Gamma_w}} \leqslant r_x\right)$

$B_3 = (t + 1 \geqslant N_{\max})$

$t \leftarrow t + 1$

end while

return x^t

In alg. SI6.1, L_A is the norm of the operator A, following the predication for the acceleration, we set $\sigma_0 = \frac{1}{\tau_0 L_A^2}$ and $\gamma = 2\frac{\|\hat{\rho} - \rho_0\|}{\tau_0}$ with the distance $\|\hat{\rho} - \rho_0\|$ being approximated by a rough upper bound of the true (unknown) distance. The upper bound of the distance $\|\hat{\rho} - \rho_0\|$ is approximated by the distance between two extreme

cases with constant concentrations 0 and ρ_B, hence we set it to $\sqrt{N}\rho_B$. The acceleration parameter should depend on the relative strength between the likelihood and the a priori, but we have fixed that value. It does not seem to be strongly dependent within the tested range. In practice, the symmetric positive definite matrix Γ_w is taken as the identity matrix and the relative tolerance in the data space r_y and in the primal space r_x are both set to an arbitrarily small value, e.g. 10^{-3}.

Using the notations from the paper by Chambolle and Pock 2011 (Chambolle & Pock, 2011), the functions are

$$F(z) = \|\mathbf{y}_S^m - z\|_\Gamma^2 = (\mathbf{y}_S^m - z)^T(\Gamma_S^m)^{-1}(\mathbf{y}_S^m - z) \quad (41)$$

$$G(x) = \begin{cases} 0 & \text{if } \forall i, x_i \geqslant 0 \\ \infty & \text{otherwise} \end{cases} \quad (42)$$

$$A = \bar{A}_S^m \quad (43)$$

and the proximal operators for the convex conjugate $F^\star$ and for G are

$$\mathrm{prox}_{\sigma F^\star}(s) = \left(\mathbb{I} + \frac{\sigma}{2}\Gamma_S^m\right)^{-1}(s - \sigma\mathbf{y}_S^m) \quad (44)$$

$$\mathrm{prox}_{\tau G}(x) = (x)^+ \quad (45)$$

where each entry of the vector $(x)^+$ is the corresponding entry of x if the entry is positive and 0 otherwise.

Beside the apparent complexity of the concepts deployed for the definition of the proximal operator and the convex conjugate, the formula for the operators (and their implementations) are rather simple. The inverse of $\mathbb{I}+\frac{\sigma}{2}\Gamma_S^m$ only needs to be computed once, and it can take advantage of the eigen decomposition of Γ_S^m.

6.2. Sampling using Metropolis-Hastings

The goal of sampling is to estimate the mean $\mu_{\rho|A^m,\mathbf{y}}$ and covariance $\Gamma_{\rho|A^m,\mathbf{y}}$ of the posterior distribution $\mathsf{P}(\rho|A^m, \mathbf{y})$ from samples $(\zeta_i)_{1\leqslant i\leqslant N_{\text{sample}}}$ generated by the algorithm SI6.2. Other quantities defined in section 3 also rely on them. The Metropolis-

Hastings algorithm has been described several times, notably by the authors it is named after, Metropolis in 1949 (Metropolis & Ulam, 1949) and Hastings in 1970 (Hastings, 1970). MH has also been refined in some cases to improve its performances (Pereyra *et al.*, 2015). It is a sampling procedure that is based on the Monte Carlo method. The samples proposed by a transition mechanism, i.e. a way to jump from one state to another, are always accepted if they increase the probability distribution, and they are not always rejected if they induce a decrease of the density. When the proposed sample ρ^{prop} is less favorable, it is accepted with a probability that decreases with the ratio of probability densities

$$\tau^{MH}(\rho^{\text{prop}}, \rho^{\text{curr}}) = \frac{\mathsf{P}(\rho^{\text{prop}}|A^m, \mathbf{y})}{\mathsf{P}(\rho^{\text{curr}}|A^m, \mathbf{y})} \frac{q^{MH}(\rho^{\text{curr}}|\rho^{\text{prop}})}{q^{MH}(\rho^{\text{prop}}|\rho^{\text{curr}})} \tag{46}$$

The transition mechanism q^{MH} should be designed similar to the target sampled distribution so that it efficiently samples $\mathsf{P}(\rho|A^m, \mathbf{y})$. Here we choose a symmetric kernel – $q^{MH}(\rho^{\text{curr}}|\rho^{\text{prop}}) = q^{MH}(\rho^{\text{prop}}|\rho^{\text{curr}})$ – defined by a Gaussian distribution, and the proposed sample is generated as:

$$\rho^{\text{prop}} = \rho^{\text{curr}} + \Gamma_{MH}^{\frac{1}{2}}\eta, \quad \text{with} \quad \eta \sim \mathcal{N}(0, \mathbb{I}_N) \tag{47}$$

with Γ_{MH} a covariance matrix of a correlated process, i.e. the off diagonal coefficients are significantly non-zero. It is defined by the entries

$$(\Gamma_{MH})_{i,j} = (\sigma_i^{MH})^2 e^{-\frac{(i-j)^2}{2\delta_{MH}^2}} \tag{48}$$

where $\sigma_i^{MH} = \sqrt{5} \times 10^{-2}\rho_B \ll \rho_B$ and $\delta_{MH} = 5$ for $N = 100$, and the dimension of ρ_S is $N_S - N_b - 1 \in \{10, 14\}$ depending on the profile. This communication mechanism ensures that the proposed state deviates by a small difference σ^{MH} and that the difference is somewhat smooth δ_{MH}.

Algorithm SI6.2 is formulated in terms of probability density, but for numerical reasons, it is here implemented in terms of the logarithm of the distributions. For sampling

Algorithm SI6.2: Statement of Metropolis-Hasting for sampling the distribution $\mathsf{P}(\rho|A^m, \mathbf{y})$ using a symmetric transition mechanism.

Require: ζ_0 initial concentration profile, number of iterations N_{sample}, q^{MH} communication mechanism

for $i = 1$ to $i \leqslant N_{\text{sample}}$, $i++$ **do**

Draw a sample $\zeta_i^{\text{prop}} \sim q^{MH}(\bullet|\zeta_{i-1})$ from eq. SI(47)

Compute the acceptance rate $\tau^{MH}(\zeta_i^{\text{prop}}, \zeta_{i-1})$ from eq. SI(46)

if $\tau^{MH}(\zeta_i^{\text{prop}}, \zeta_{i-1}) \geqslant 1$ **then**

Accept proposed state unconditionally

$\zeta_i = \zeta_i^{\text{prop}}$

else

Accept proposed state with probability $\tau^{MH}(\zeta_i^{\text{prop}}, \zeta_{i-1})$

Draw $u \sim \mathcal{U}([0,1])$

if $u \geqslant \tau^{MH}(\zeta_i^{\text{prop}}, \zeta_{i-1})$ **then**

$\zeta_i = \zeta_i^{\text{prop}}$

else

$\zeta_i = \zeta_{i-1}$

end if

end if

end for

return collection of states $(\zeta_i)_{1 \leqslant i \leqslant N_{\text{sample}}}$

the distribution $\mathsf{P}(\rho|A^m, \mathbf{y})$, we set the initial state ζ_0 of the collection $(\zeta_i)_{1 \leqslant i \leqslant N_{\text{sample}}}$ to $\hat{\rho}|A^m, \mathbf{y}$, so that the burn in period is very short.

7. Further results

7.1. Attenuation length sampling domain

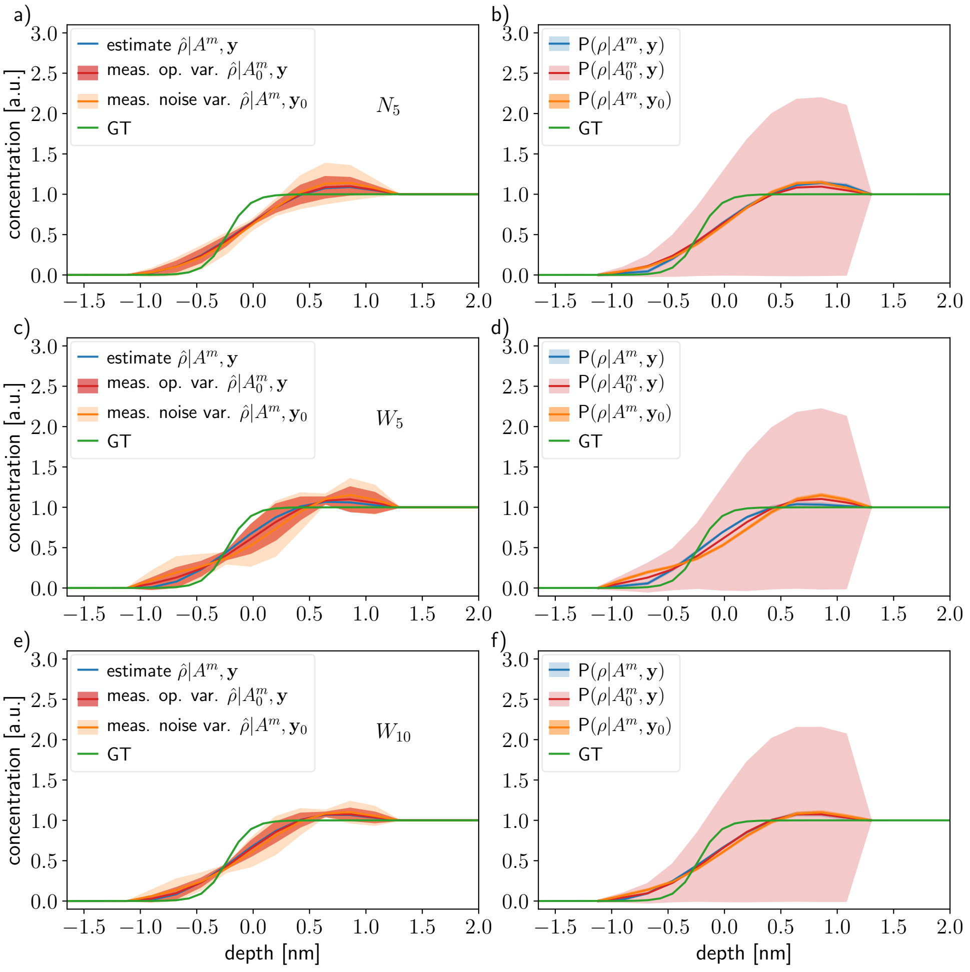

Fig. 2. Reconstruction of concentration profile for three different simulated experimental acquisition setups: a) and b) 5 attenuation lengths over the range $[1.62, 1.95]$ nm (N_5), d)and d) 5 attenuation lengths over the range $[1.28, 5.5]$ nm (W_5), and e) and f) 10 attenuation lengths over the range $[1.28, 5.5]$ nm (W_{10}). The panels a), c), and e) show the estimates and the different variability, with respect to the measurement noise in orange ($\Gamma_{\hat{\rho}|A^m,\mathbf{y}_0}$), and with respect to the measurement model error in red ($\Gamma_{\hat{\rho}|A_0^m\mathbf{y}}$). The panels b) d) and f) show the conditional posterior probability $\mathsf{P}(\rho|A^m, \mathbf{y})$ (blue), and the marginals $\mathsf{P}(\rho|A^m, \mathbf{y}_0)$ (orange) and $\mathsf{P}(\rho|A_0^m, \mathbf{y})$ (red).

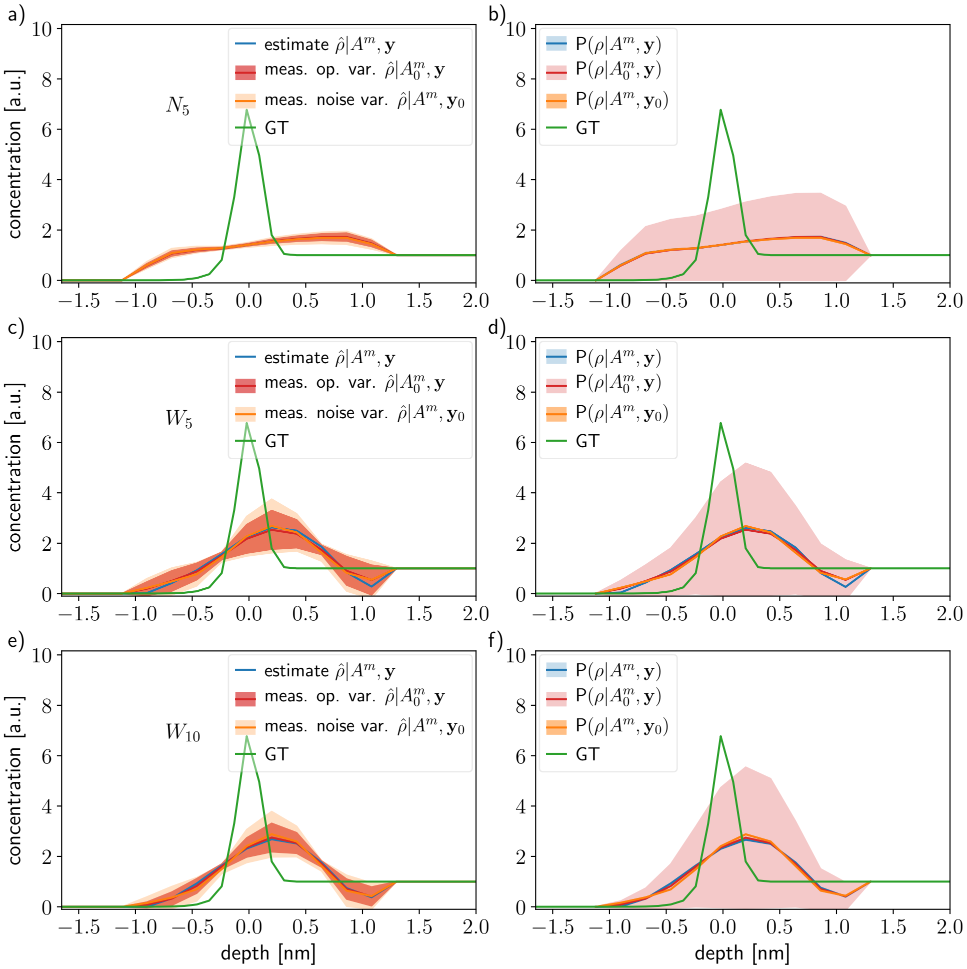

Fig. 3. Reconstruction of concentration profile for three different simulated experimental acquisition setups: a) and b) 5 attenuation lengths over the range $[1.62, 1.95]$ nm (N_5), d)and d) 5 attenuation lengths over the range $[1.28, 5.5]$ nm (W_5), and e) and f) 10 attenuation lengths over the range $[1.28, 5.5]$ nm (W_{10}). The panels a), c) and e) show the estimates and the different variability, with respect to the measurement noise in orange ($\Gamma_{\hat{\rho}|A^m,\mathbf{y}_0}$), and with respect to the measurement model error in red ($\Gamma_{\hat{\rho}|A_0^m\mathbf{y}}$). The panels b) d) and f) show the conditional posterior probability $\mathsf{P}(\rho|A^m, \mathbf{y})$ (blue), and the marginals $\mathsf{P}(\rho|A^m, \mathbf{y}_0)$ (orange) and $\mathsf{P}(\rho|A_0^m, \mathbf{y})$ (red).

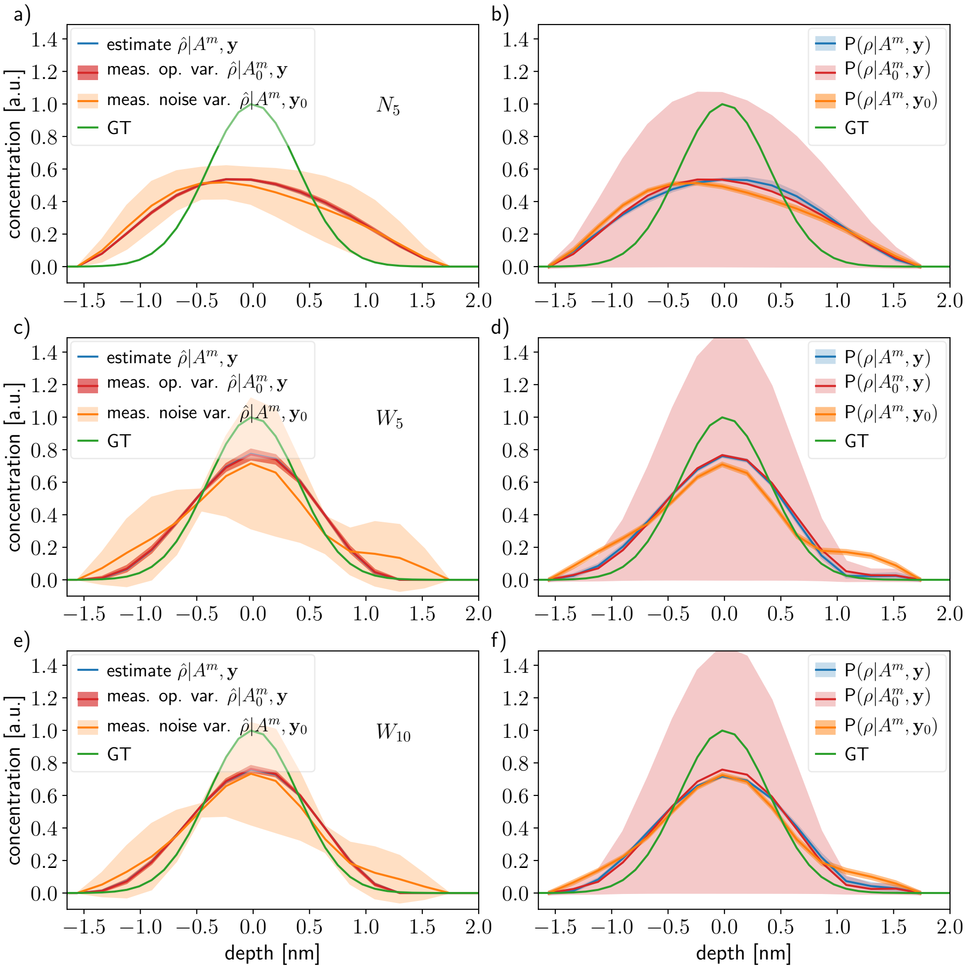

Fig. 4. Reconstruction of concentration profile for three different simulated experimental acquisition setups: a) and b) 5 attenuation lengths over the range $[1.62, 1.95]$ nm (N_5), d)and d) 5 attenuation lengths over the range $[1.28, 5.5]$ nm (W_5), and e) and f) 10 attenuation lengths over the range $[1.28, 5.5]$ nm (W_{10}). The panels a), c), and e) show the estimates and the different variability, with respect to the measurement noise in orange ($\Gamma_{\hat{\rho}|A^m,\mathbf{y}_0}$), and with respect to the measurement model error in red ($\Gamma_{\hat{\rho}|A_0^m\mathbf{y}}$). The panels b) d) and f) show the conditional posterior probability $\mathsf{P}(\rho|A^m,\mathbf{y})$ (blue), and the marginals $\mathsf{P}(\rho|A^m,\mathbf{y}_0)$ (orange) and $\mathsf{P}(\rho|A_0^m,\mathbf{y})$ (red).

7.2. Model error: λ_e uncertainty

7.2.1. Independent errors

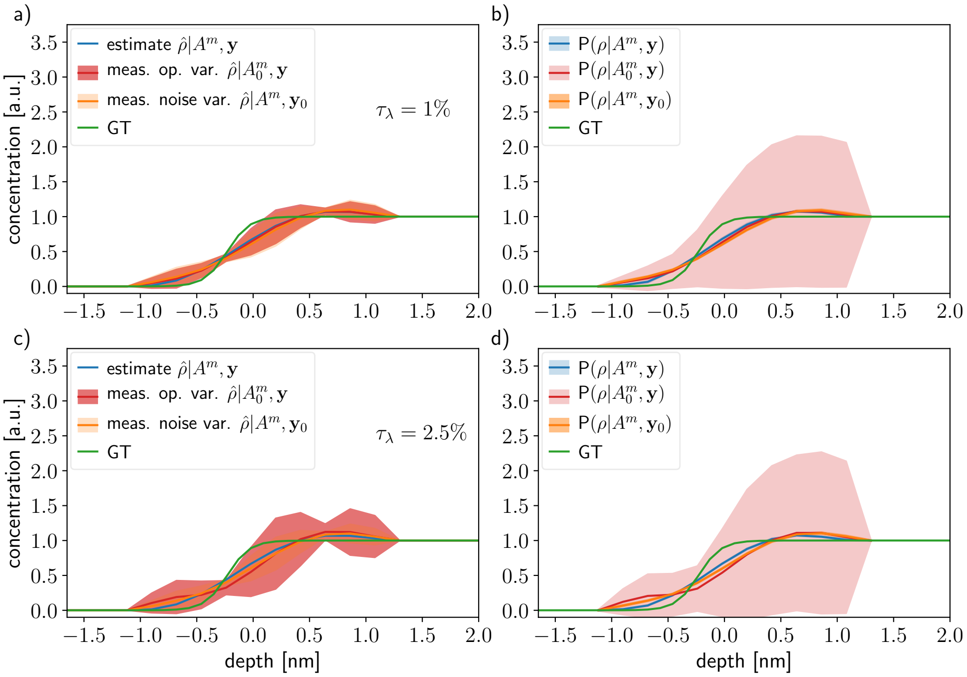

Fig. 5. Profile reconstruction in the case W_{10} for two levels of attenuation length uncertainty: a) and b) $\tau_\lambda = 1\%$, and c) and d) $\tau_\lambda = 2.5\%$. The green curves represent the GT. In panels a) and c), the profile reconstruction are plotted in blue ($\hat{\rho}|A^m, \mathbf{y}$), orange ($\hat{\rho}|A^m, \mathbf{y}_0$) and red ($\hat{\rho}|A_0^m, \mathbf{y}$) with their respective variabilities as shaded areas. In panels b) and d), the a posteriori ($\mathsf{P}(\rho|A^m, \mathbf{y})$) is represented in blue and the marginals in orange ($\mathsf{P}(\rho|A^m, \mathbf{y}_0)$) and red ($\mathsf{P}(\rho|A_0^m, \mathbf{y})$).

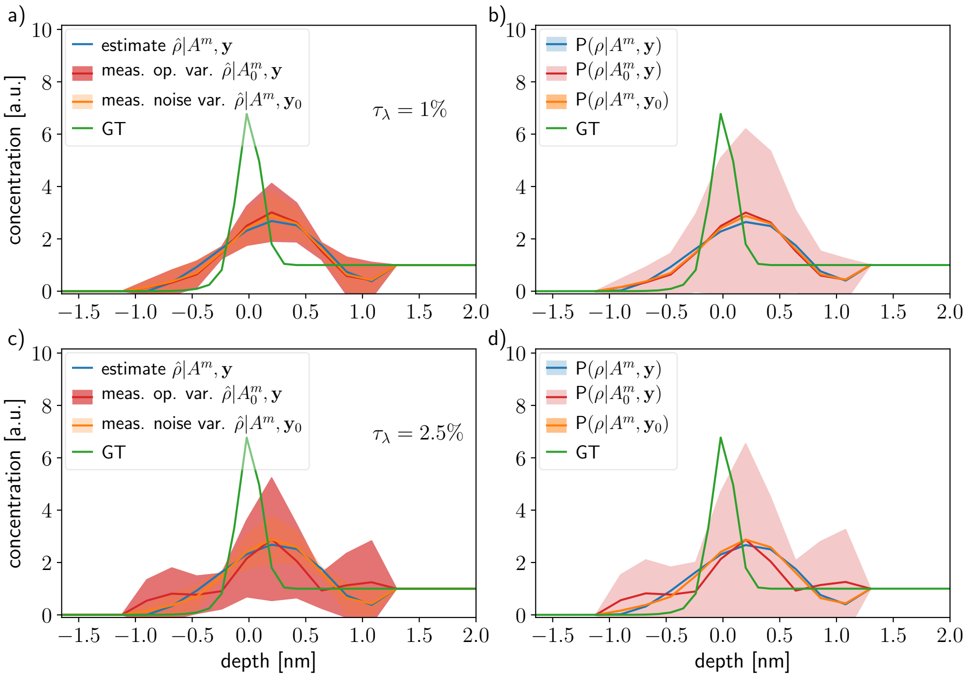

Fig. 6. Profile reconstruction in the case W_{10} for two levels of attenuation length uncertainty: a) and b) $\tau_\lambda = 1\%$, and c) and d) $\tau_\lambda = 2.5\%$. The green curves represent the GT. In panels a) and c), the profile reconstruction are plotted in blue $(\hat{\rho}|A^m, \mathbf{y})$, orange $(\hat{\rho}|A^m, \mathbf{y}_0)$ and red $(\hat{\rho}|A_0^m, \mathbf{y})$ with their respective variabilities as shaded areas. In panels b) and d), the a posteriori $(\mathsf{P}(\rho|A^m, \mathbf{y}))$ is represented in blue, and the marginals in orange $(\mathsf{P}(\rho|A^m, \mathbf{y}_0))$ and red $(\mathsf{P}(\rho|A_0^m, \mathbf{y}))$.

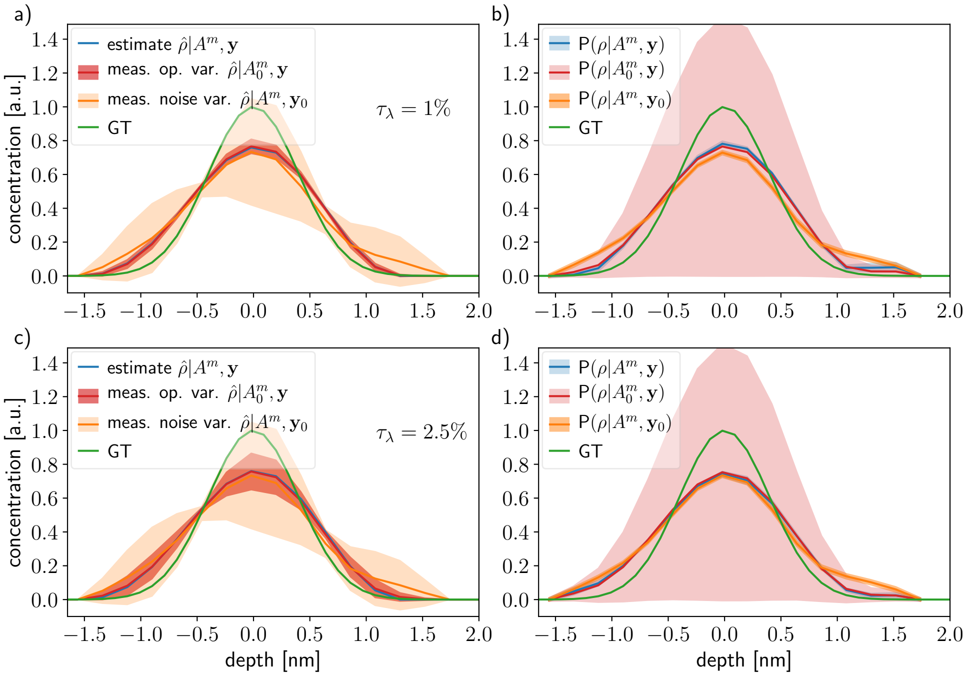

Fig. 7. Profile reconstruction in the case W_{10} for two levels of attenuation length uncertainty: a) and b) $\tau_\lambda = 1\%$, and c) and d) $\tau_\lambda = 2.5\%$. The green curves represent the GT. In panels a) and c), the profile reconstruction are plotted in blue ($\hat{\rho}|A^m, \mathbf{y}$), orange ($\hat{\rho}|A^m, \mathbf{y}_0$) and red ($\hat{\rho}|A^m_0, \mathbf{y}$) with their respective variabilities as shaded areas. In panels b) and d), the a posteriori ($\mathsf{P}(\rho|A^m, \mathbf{y})$) in represented in blue and the marginals in orange ($\mathsf{P}(\rho|A^m, \mathbf{y}_0)$) and red ($\mathsf{P}(\rho|A^m_0, \mathbf{y})$).

7.2.2. Global error

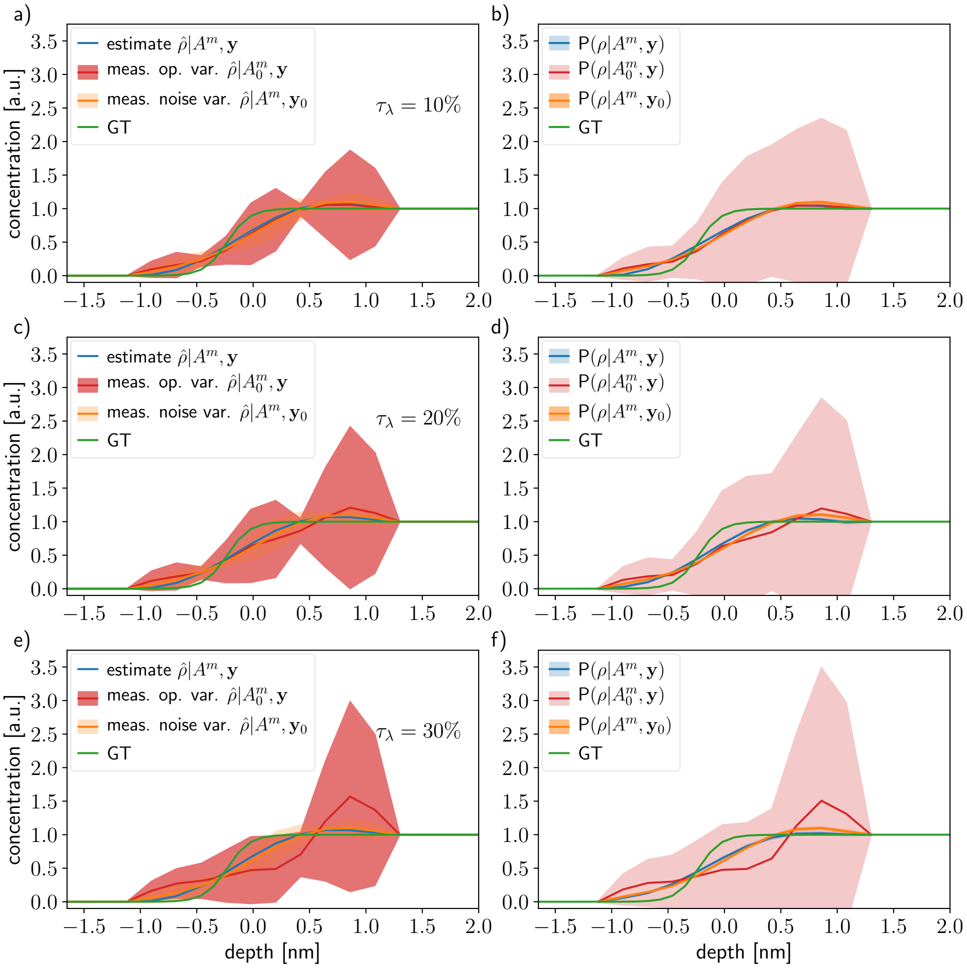

Fig. 8. Profile reconstruction in the case W_{10} for three levels of global attenuation length uncertainty: a) and b) $\tau_\lambda = 10\%$, c) and d) $\tau_\lambda = 20\%$, and e) and f) $\tau_\lambda = 30\%$. The green curves represents the GT. In the panels a), c) and e), the profile reconstruction are plotted in blue ($\hat{\rho}|A^m, \mathbf{y}$), orange ($\hat{\rho}|A^m, \mathbf{y}_0$) and red ($\hat{\rho}|A^m_0, \mathbf{y}$) with their respective variabilities as shaded areas. In the panels b), d), and f), the a posteriori ($\mathsf{P}(\rho|A^m, \mathbf{y})$) in represented in blue and the marginals in orange ($\mathsf{P}(\rho|A^m, \mathbf{y}_0)$) and red ($\mathsf{P}(\rho|A^m_0, \mathbf{y})$).

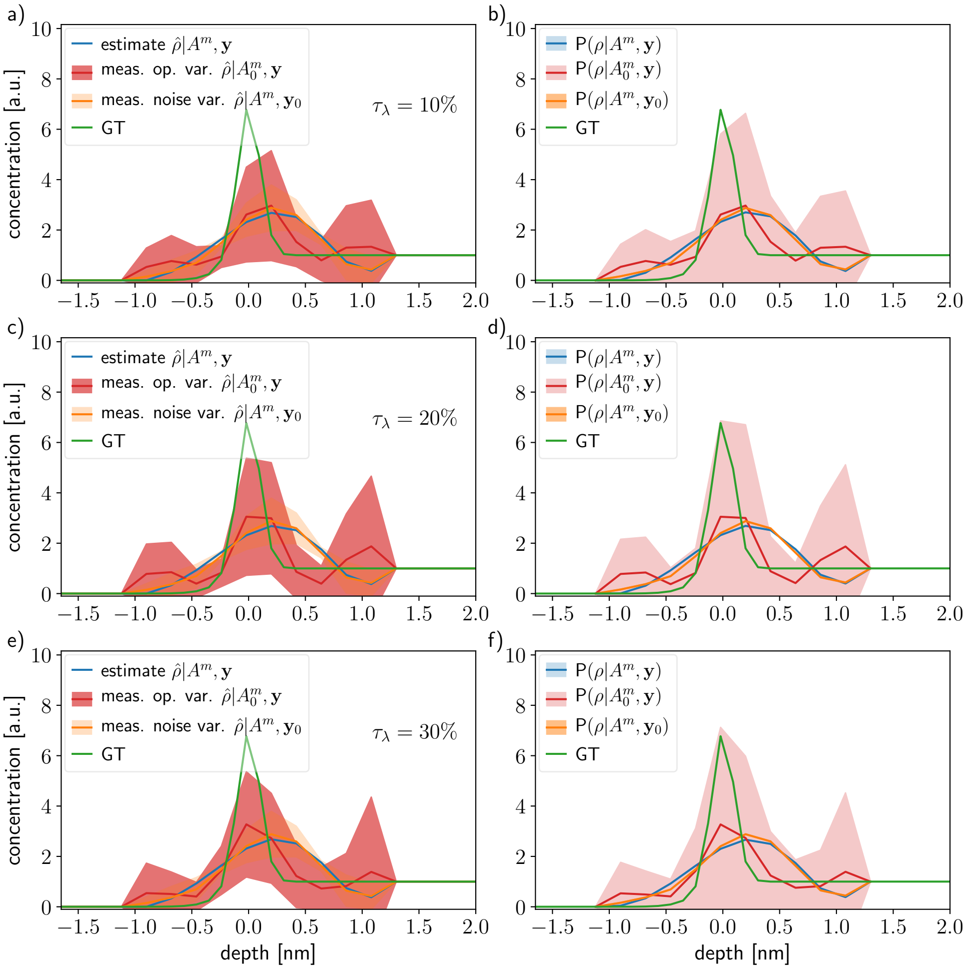

Fig. 9. Profile reconstruction in the case W_{10} for three levels of global attenuation length uncertainty: a) and b) $\tau_\lambda = 10\%$, c) and d) $\tau_\lambda = 20\%$, and e) and f) $\tau_\lambda = 30\%$. The green curves represents the GT. In the panels a), c) and e), the profile reconstruction are plotted in blue $(\hat{\rho}|A^m, \mathbf{y})$, orange $(\hat{\rho}|A^m, \mathbf{y}_0)$ and red $(\hat{\rho}|A_0^m, \mathbf{y})$ with their respective variabilities as shaded areas. In the panels b), d), and f), the a posteriori $(\mathsf{P}(\rho|A^m, \mathbf{y}))$ in represented in blue and the marginals in orange $(\mathsf{P}(\rho|A^m, \mathbf{y}_0))$ and red $(\mathsf{P}(\rho|A_0^m, \mathbf{y}))$.

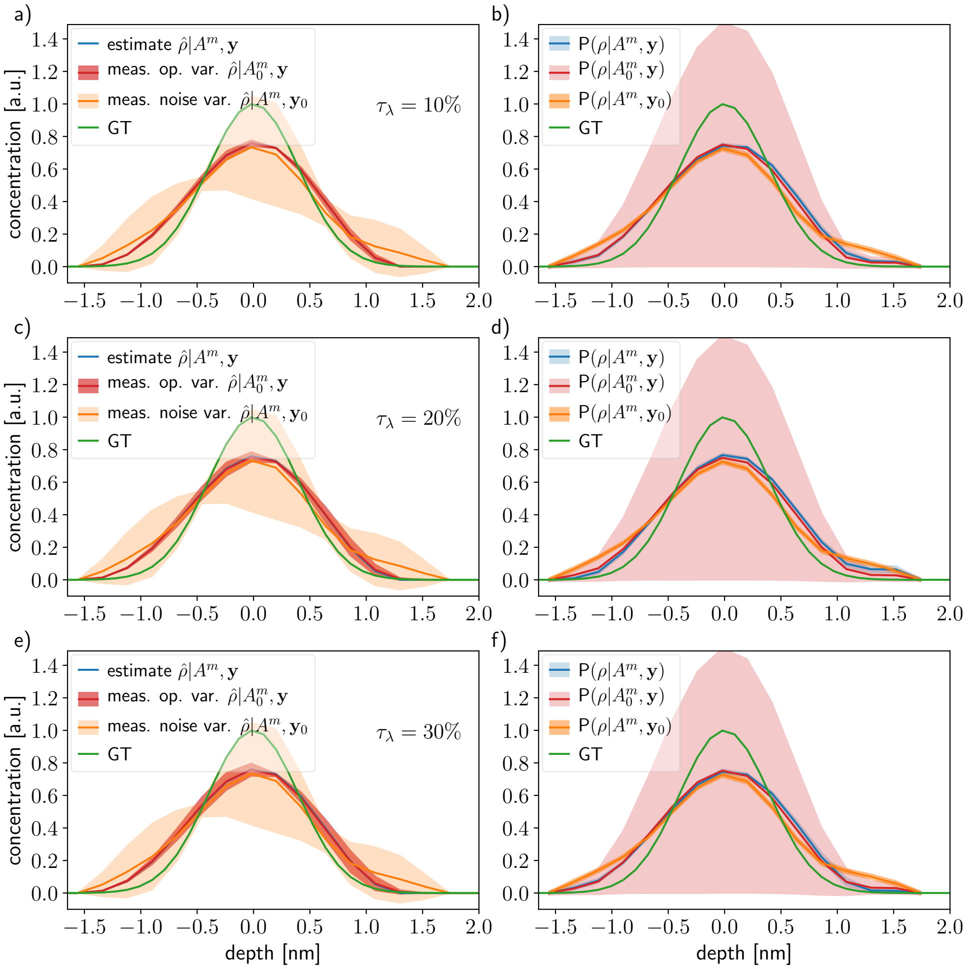

Fig. 10. Profile reconstruction in the case W_{10} for three levels of global attenuation length uncertainty: a) and b) $\tau_\lambda = 10\%$, c) and d) $\tau_\lambda = 20\%$, and e) and f) $\tau_\lambda = 30\%$. The green curves represents the GT. In the panels a), c) and e), the profile reconstruction are plotted in blue ($\hat{\rho}|A^m, \mathbf{y}$), orange ($\hat{\rho}|A^m, \mathbf{y}_0$) and red ($\hat{\rho}|A_0^m, \mathbf{y}$) with their respective variabilities as shaded areas. In the panels b), d), and f), the a posteriori ($\mathsf{P}(\rho|A^m, \mathbf{y})$) in represented in blue and the marginals in orange ($\mathsf{P}(\rho|A^m, \mathbf{y}_0)$) and red ($\mathsf{P}(\rho|A_0^m, \mathbf{y})$).

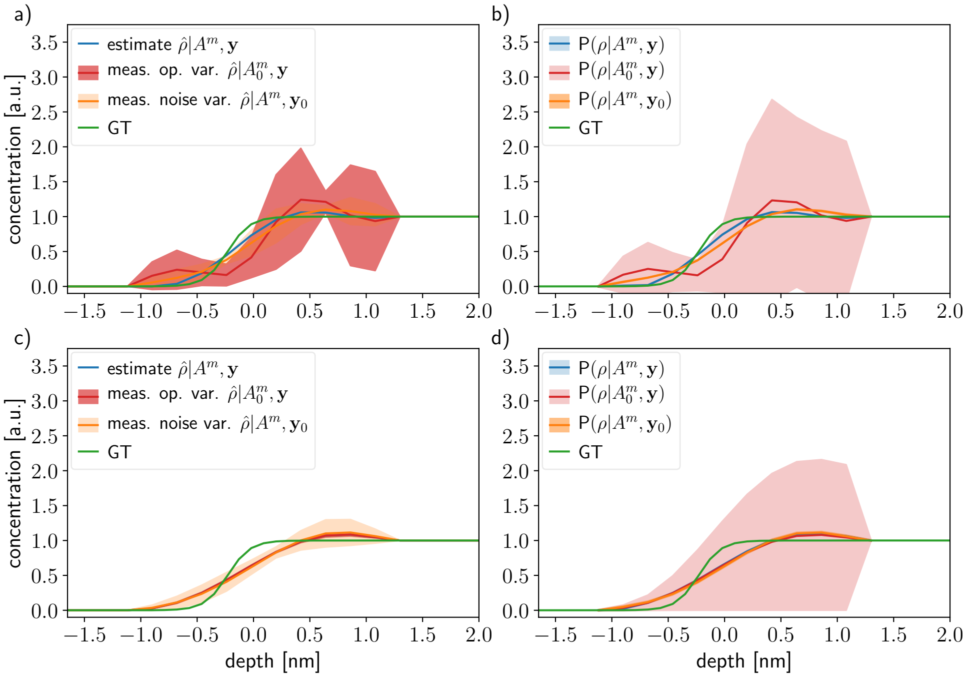

Fig. 11. Reconstruction of concentration profile for two levels of acquisition noise: panels a and b) very low ($\sigma_k = 0.01$, SNR $= \frac{\mathbb{E}[I_k^m]^2}{\sigma_k^2} \in [250 \times 10^3, 16 \times 10^6]$), and panels c) and d) very high ($\sigma_k = 0.5$, SNR $\in [100, 6400]$). In the panels a) and c), the profile reconstruction are plotted in blue ($\hat{\rho}|A^m, \mathbf{y}$), orange ($\hat{\rho}|A^m, \mathbf{y}_0$) and red ($\hat{\rho}|A_0^m, \mathbf{y}$) with their respective variabilities as shaded areas. In the panels b) and d), the a posteriori ($\mathsf{P}(\rho|A^m, \mathbf{y})$) in represented in blue and the marginals in orange ($\mathsf{P}(\rho|A^m, \mathbf{y}_0)$) and red ($\mathsf{P}(\rho|A_0^m, \mathbf{y})$).

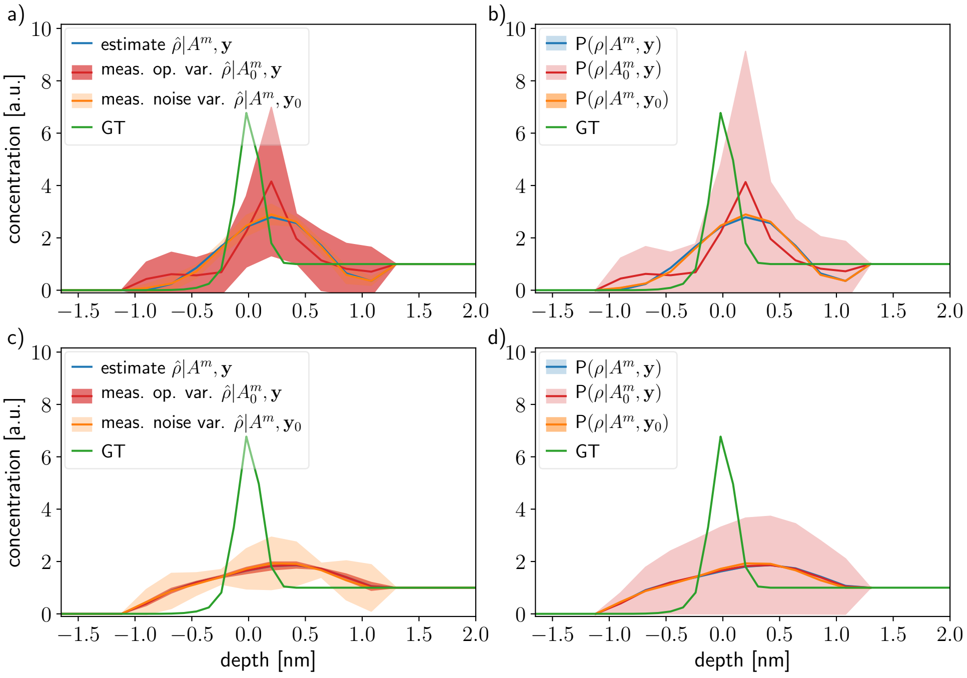

Fig. 12. Reconstruction of concentration profile for two levels of acquisition noise: panels a and b) very low ($\sigma_k = 0.01$, SNR $= \frac{\mathbb{E}[I_k^m]^2}{\sigma_k^2} \in [250 \times 10^3, 16 \times 10^6]$), and panels c) and d) very high ($\sigma_k = 0.5$, SNR $\in [100, 6400]$). In the panels a) and c), the profile reconstruction are plotted in blue ($\hat{\rho}|A^m, \mathbf{y}$), orange ($\hat{\rho}|A^m, \mathbf{y}_0$) and red ($\hat{\rho}|A_0^m, \mathbf{y}$) with their respective variabilities as shaded areas. In the panels b) and d), the a posteriori ($\mathsf{P}(\rho|A^m, \mathbf{y})$) in represented in blue and the marginals in orange ($\mathsf{P}(\rho|A^m, \mathbf{y}_0)$) and red ($\mathsf{P}(\rho|A_0^m, \mathbf{y})$).

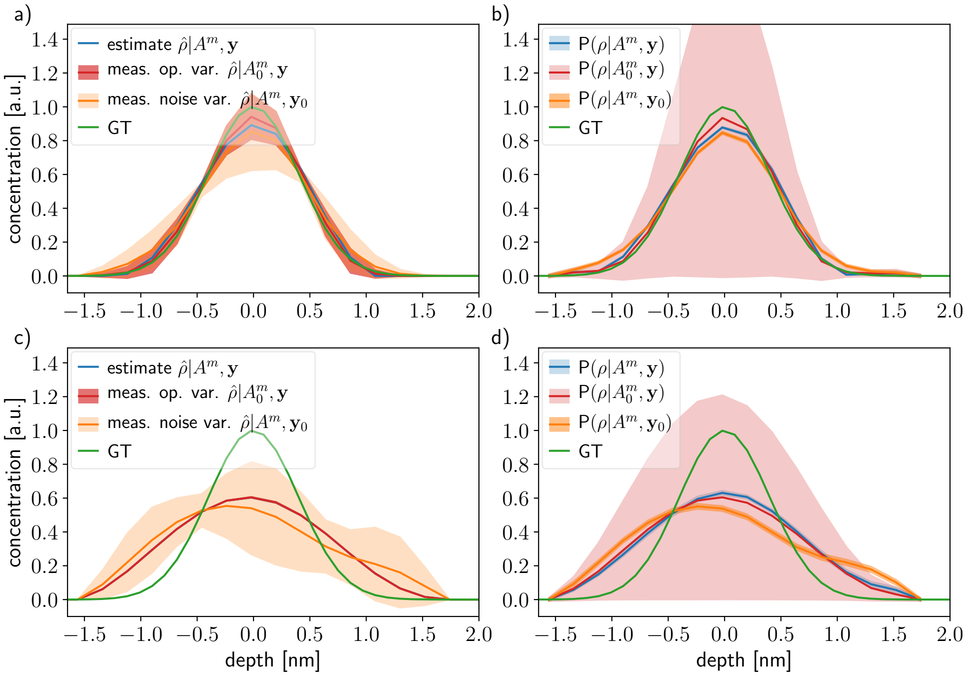

Fig. 13. Reconstruction of concentration profile for two levels of acquisition noise: panels a and b) very low ($\sigma_k = 0.01$, SNR $= \frac{\mathbb{E}[I_k^m]^2}{\sigma_k^2} \in [250 \times 10^3, 16 \times 10^6]$), and panels c) and d) very high ($\sigma_k = 0.5$, SNR $\in [100, 6400]$). In panels a) and c), the profile reconstruction are plotted in blue ($\hat{\rho}|A^m, \mathbf{y}$), orange ($\hat{\rho}|A^m, \mathbf{y}_0$) and red ($\hat{\rho}|A_0^m, \mathbf{y}$) with their respective variabilities as shaded areas. In panels b) and d), the a posteriori ($\mathsf{P}(\rho|A^m, \mathbf{y})$) is represented in blue, and the marginals in orange ($\mathsf{P}(\rho|A^m, \mathbf{y}_0)$) and red ($\mathsf{P}(\rho|A_0^m, \mathbf{y})$).

8. List of notations

8.1. Forward model

Table 1: List of notations and symbols used for the forward model

Name	Units	Definition
$M = (x, y, z)$	$[\mathrm{m}, \mathrm{m}, \mathrm{m}]$	
$M = (r, \theta, y)$	$[\mathrm{m}, \mathrm{rad}, \mathrm{m}]$	point in the sample
$M = (r, \theta, \phi)$	$[\mathrm{m}, \mathrm{rad}, \mathrm{rad}]$	
$P = (x_0, y_0, z_0)$	$[\mathrm{m}, \mathrm{m}, \mathrm{m}]$	
$P = (R_0, \theta_0, y_0)$	$[\mathrm{m}, \mathrm{rad}, \mathrm{m}]$	location of the aperture of the analyzer
$P = (R_0, \theta_0, \phi_0)$	$[\mathrm{m}, \mathrm{rad}, \mathrm{rad}]$	
θ	[rad]	angular direction in polar and spherical coordinates, and angle between the polarization vector of the light and the direction of the emitted PE
Ω_θ	-	angular integration domain
α_θ	[sr]	angular aperture of the analyzer
(ω, β)	[rad,rad]	direction angles of the parametric curve M_s
ξ	[rad]	angle between MP and OM in spherical approximation
s	[m]	signed distance from point M
μ_0	[m]	radius of the LJ
$\tau_{\max}$	[m]	distance $\|MP\|$
$\bar{\tau}$	[m]	upper limit of the distance integral
M_s	-	parametric curve leading to the surface of the liquid in the direction of the analyzer
$d(M)$	[m]	signed distance between M and the sample surface
Δ_r	[m]	characteristic transition length associated with the sample
ρ	$[\mathrm{m}^{-3}]$	concentration of orbital
ρ_{tot}	$[\mathrm{m}^{-3}]$	sum of the concentration of all the species
$H(\rho, \lambda_e)$	[-]	geometry factor
Ω_V	-	integration domain covering the sample
$\Phi(\theta)$	[rad]	azimuth angle amplitude in the volume integration domain Ω_V in spherical coordinates
ℓ	-	kinetic energy index: discretization point
L	-	number of discretization points or channel nodes
λ_e	[m]	attenuation length
K_e	[eV]	kinetic energy
$K_{e_\ell}^k$	[eV]	ℓ^{th} kinetic energy discretization point
φ_ℓ^k	[s]	efficiency function of the analyzer
σ_{T_k}	[eV]	spread in kinetic energy (standard deviation)
T_k	[s]	gain of the analyzer (the transmission function)
$\Omega_{K_e}^{k,\chi}$	[eV]	kinetic energy integration domain covering the photoionization cross-section density support

k	-	photon energy index
K	-	number of photon energy
ν	[Hz]	frequency of the light
$h\nu$	[eV]	photon energy
ν_k	[Hz]	k^{th} central frequency value of the exciting light
Δ_{ν_k}	[Hz]	spectral spread of the light for the kth frequency
$\delta(\nu - \nu_k)$	[eV^{-1}]	Dirac distribution modeling the monochromatic spectral density
$F(\nu_k)$	[photon s^{-1}]	total photon flux
$f(M)$	[m^{-2}]	beam profile: density in the plane orthogonal to the propagation direction of the light
$f(\nu, M)$	[m^{-2} eV^{-1} s^{-1}]	the photon flux density
Ω_ν	[eV]	photon energy integration domain
α_k	[m^{-2}]	alignment parameter
$\Psi_{i,N}$, $\Psi_{f,N}$	-	initial and final states of a quantum system with N electrons
φ_i^{1s}, $\varphi_i^{K_e}$	-	states of the emitted electron before and after the interaction with a photon
Δ	[eV]	interaction potential
$\mathbf{A}$	[V s m^{-1}]	vector potential
χ	-	designation of the orbital of an element, e.g. C1s
σ_χ	-	photoionization cross-section
$\sigma_\chi(\nu)$	[m^2]	total photoionization cross-section
$\sigma_\chi(\nu, \theta)$	[m^2 sr^{-1}]	differential photoionization cross-section
$\sigma_\chi(\nu, K_e)$	[m^2 eV^{-1}]	photoionization cross-section density
$\sigma_\chi(\nu, K_e, \theta)$	[m^2 eV^{-1} sr^{-1}]	differential photoionization cross-section density
m	-	peak area index (related to chemical state)
$\mathfrak{M}$	-	number of peaks per spectra
$p^{m,k}$	-	probabilities of interaction photon/chemical-state-m for a photon energy $h\nu_k$
$\sigma_\chi^{m,k}(K_e)$	[m^{2}eV^{-1}]	m^{th} peak of the photoionization cross-section density for photon energy $h\nu_k$
$\tilde{\sigma}_\chi^k(K_e)$, $\tilde{\sigma}_\chi^{m,k}(K_e)$	[eV^{-1}]	photoionization cross-section probability density with photon energy ν_k and for the mth peak
$I(\nu, K_e, \theta, x, y)$	[m^{-2} eV^{-1} s^{-1}]	spectral density of the PE flux
$J(\nu_k, K_e)$	[electron s^{-1}]	PE rate
$I(\nu_k, K_{e_\ell}^k)$	[counts]	counts of PEs of interest
$I_{\text{bg}}(\nu_k, K_{e_\ell}^k)$	[counts]	background signal
ε_ℓ^k	[counts]	measurement noise of the ℓth point in the spectrum acquired at the photon energy $h\nu_k$
$(\sigma_\ell^k)^2$	[counts^2]	variance of the measurement noise ε_ℓ^k
$I_{\text{tot}}(\nu_k, K_{e_\ell}^k)$	[counts]	total measured signal
I_k^m	[counts]	peak area from species in m^{th} chemical state for the photon energy $h\nu_k$

Table 2: List of notations for the inversion methodologies and results

Name	Units	Definition
$\mathbf{y}$	-	measurement vector
$\mathbf{y}^m$,$\mathbf{y}^m_S$	-	m^{th} normalized peak area measurement vector (complete and truncated model)
$\mathbf{y}_D$,$\mathbf{y}_{D_S}$	-	expected values of the a priori for the concentration profile estimation model (complete and truncated)
ρ	[m^{-3}]	the concentration profile
ρ_S	[m^{-3}]	the concentration profile subset corresponding to the first layers
ρ_B	[m^{-3}]	the bulk concentration
σ_B^2	[(m^{-3})^2]	variance of the known values of the concentration profile (ρ_1 and ρ_B)
δ_B	[m]	thickness of the surface layer: deeper than δ_B, the concentration is that of the bulk
D, D_S, D_b, D_B	-	second order difference operator and its restrictions
I_k	[counts]	total area under the curve: counts of all the PE for the photon energy $h\nu_k$
R_k^{reg}	-	augmented state measurement model for the estimation of the cross-section probability density
H^k, $H^k_{\ell,n}$	[s eV m^3]	matrix and its element for the kth frequency geometry factor including the analyzer function φ_ℓ^k
$\Gamma_{A^m_k}$	[(m^3)^2]	covariance matrix of the peak area model for the kth measurement
A^m, A^m_S, A^m_b, A^m_B	[m^3]	m^{th} normalized peak area operator for all photon energy (complete and truncated model)
$\bar{A}^m$, $\bar{A}^m_S$	-	augmented measurement model for the mth normalized peak area (complete and truncated model)
$\mathsf{P}(\rho\|A^m,\mathbf{y})$	-	*a posteriori* probability density of the concentration profile ρ
$\mathsf{P}(\mathbf{y}\|A^m,\rho)$	-	peak area measurement likelihood probability
$\mathsf{P}(\mathbf{y})$	-	*a priori* probability of any measurement $\mathbf{y}$ or noise distribution
$\mathsf{P}(A^m)$	-	*a priori* probability distribution of the measurement operator, reflects the modeling uncertainties
$\mathsf{P}(A^m,\rho)$	-	joint a priori probability distribution of the measurement operator and the state
$\Omega_{\mathbf{y}}$, Ω_{A^m}	-	set of all possible values of $\mathbf{y}$ and A^m
$\mathsf{P}(I_k)$	-	probability density of the total PE count for the photon energy $h\nu_k$
$\bar{I}_k$	[counts]	mean value of the total PE count for the photon energy $h\nu_k$

Ω_{I_k}	-	interval of all possible values of the total PE count for the photon energy $h\nu_k$
$\|x\|_W^2$	-	notation for the quadratic $x^tW^{-1}x$ with W positive definite
$O\left(x^\gamma\right)$	-	Landau notation for bonded terms of order $\geqslant \gamma$
$\mathbb{I}_L$	-	L-order identity matrix
$\hat{\rho}\|A^m, \mathbf{y}$	$[\mathrm{m}^{-3}]$	MAP estimate of the profile concentration
$\hat{\sigma}^k_{\mathrm{C1s}}\|I_k, \mathbf{y}$	$[\mathrm{eV}^{-1}]$	MAP estimate of the cross-section probability density
$\mathsf{P}(\tilde{\sigma}^k_{\mathrm{C1s}}\|I_k, \mathbf{y})$	-	a posteriori probability density modelling the cross-section density
Γ_D, Γ_{D_S}	-	covariance matrix of the a priori distribution of ρ and ρ_S
σ_k^2	[counts^2]	variance corresponding to the peak areas obtain from the photon energy ν_k
Γ	-	covariance matrix of the peak area measurement noise (diagonal entries $(\frac{\sigma_k}{\alpha_k T_k F(\nu_k)\sigma_{\mathrm{C1s}}(\nu_k)})^2$)
Γ_I	-	covariance matrix of the augmented state (spectrum and a priori) for the estimation of the cross-section density
$\varepsilon^{m,k}$	[counts]	discretization error in the mth peak area measurement model for the photon energy $h\nu_k$
ε^k_ℓ	[counts]	measurement noise in the ℓth point in the spectrum for the photon energy $h\nu_k$
ι^k_n	-	approximation error in the geometry factor when $\lambda_e(K_e) = \lambda_e(K_k)$ for $K_e \in \Omega^{k,\chi}_{K_e}$
ι_c	-	Riemann quadrature error
ε_D, ε_{D_S}	-	a priori error term: their distribution is used for regularization of the inverse problems
ε	-	augmented state error term in the model for the estimation of the cross-section density
$\tilde{\varepsilon}^m$, $\tilde{\varepsilon}^m_k$	$[\mathrm{eV}^{-1}]$	normalized m^{th} peak area measurement noise
$\tilde{\varepsilon}^m_S$	$[\mathrm{eV}^{-1}]$	normalized m^{th} peak area measurement noise with the correction due to the truncation of the profile
$\bar{\varepsilon}^m$, $\bar{\varepsilon}^m_S$	-	noise of the augmented state measurement model for the mth peak area (complete and truncated profile)
$\mu_{\rho\|A^m,\mathbf{y}}$, $\Gamma_{\rho\|A^m,\mathbf{y}}$	$[\mathrm{m}^{-3}]$, $[(\mathrm{m}^{-3})^2]$	mean and covariance matrix of the posterior distribution $\mathsf{P}(\rho\|A^m,\mathbf{y})$
$\mu_{\rho\|A^m_0,\mathbf{y}}$, $\Gamma_{\rho\|A^m_0,\mathbf{y}}$	$[\mathrm{m}^{-3}]$, $[(\mathrm{m}^{-3})^2]$	mean and covariance matrix of the marginal distribution $\mathsf{P}(\rho\|A^m_0,\mathbf{y})$
$\mu_{\rho\|A^m,\mathbf{y}_0}$, $\Gamma_{\rho\|A^m,\mathbf{y}_0}$	$[\mathrm{m}^{-3}]$, $[(\mathrm{m}^{-3})^2]$	mean and covariance matrix of the marginal distribution $\mathsf{P}(\rho\|A^m,\mathbf{y}_0)$
$\mu_{\hat{\rho}\|A^m_0,\mathbf{y}}$, $\Gamma_{\hat{\rho}\|A^m_0,\mathbf{y}}$	$[\mathrm{m}^{-3}]$, $[(\mathrm{m}^{-3})^2]$	mean and covariance matrix of the MAP estimate of the concentration profile for a given $\mathbf{y}$
$\mu_{\hat{\rho}\|A^m,\mathbf{y}_0}$, $\Gamma_{\hat{\rho}\|A^m,\mathbf{y}_0}$	$[\mathrm{m}^{-3}]$, $[(\mathrm{m}^{-3})^2]$	mean and covariance matrix of the MAP estimate

		of the concentration profile for a given A^m
$\mu_{\hat{\sigma}^k_{\mathrm{C1s}}\|\mathbf{y}}$, $\Gamma_{\hat{\sigma}^k_{\mathrm{C1s}}\|\mathbf{y}}$	[eV^{-1}], [(eV^{-1})^2]	mean and covariance matrix of the MAP estimate of the cross-section density for a given $\mathbf{y}$
n	-	depth discretization index
N	-	number of discretization depth points
r_n	[m]	n^{th} discretization depth point
$Z_{\max}$	[m]	maximum depth at which we want to reconstruct the concentration profile
K_k	[eV]	reference kinetic energy for the kth measurement
δ_{K_e}	[eV]	discretization step in the kinetic energy domain
ΔK_e	[eV]	length of the kinetic energy support of the photoionization cross-section density $\sigma_\chi(\nu_k, K_e)$
λ_k	[m]	attenuation lengths $\lambda_e(K_k)$
ρ_n,	[m^{-3}]	value of the concentration profile ρ evaluated at r_n
$(\sigma^{m,k}_\ell)_{1\leqslant\ell\leqslant L}$	[eV^{-1}]	value of the cross-section density $\sigma^{m,k}_\chi$ evaluated at $K^k_{e_\ell}$
e_n	-	linear interpolation basis function in the depth space
f_ℓ	-	linear interpolation basis function in the kinetic energy space
c_k	-	discretization coefficient of $\varphi_{\ell k}$ normalized by T_k
τ_λ	-	uncertainty rate in the value of the attenuation length

8.3. Additional notations

Table 3: List of additional notations

Name	Units	Definition
t	-	iteration index
$N_{\max}$	-	maximum number of iterations
r_x, r_y	-	relative tolerance in the primal and data spaces
x^t, s^t	-	primal and dual variables
γ, τ^t, σ^t, θ^t	-	parameter of ALG2(Chambolle & Pock, 2011)
L_A	-	Lipschitz constant of $\bar{A}^m_S$
Ω_ρ	-	set of possible values of ρ
$\mathbf{S}^K_{++}$	-	set of symmetric definite positive matrix of order K
Γ_w	-	symmetric definite positive matrix of order K used for the stopping criterion of ALG2
B_1, B_2, B_3	-	Boolean variables for the stopping criterion of ALG2(Chambolle & Pock, 2011)
prox	-	proximal operator
G, F	-	primal and dual function defining the inverse problem 28 in the optimization algorithm
ρ^{curr}, ρ^{prop}	[m^{-3}]	current and proposed state for MH(Metropolis & Ulam, 1949; Hastings, 1970)
q^{MH}	-	transition mechanism in MH algorithm: randomly creates a new state from the current state
$\tau^{MH}(\rho^{\text{prop}}, \rho^{\text{curr}})$	-	acceptance rate

Γ_{MH}	$[(\mathrm{m}^{-3})^2]$	covariance matrix of the distribution of the transition mechanism
σ_i^{MH}	$[\mathrm{m}^{-3}]$	i^{th} diagonal entry of Γ_{MH}
δ_{MH}	-	correlation length of Γ_{MH}
N_S	-	dimension of ρ_S as well as Γ_{MH}
N_{sample}	-	number of iteration in MH algorithm
η	-	normally distributed random variable
$\mathcal{N}, \mathcal{U}$	-	normal and uniform distributions

8.4. Acronyms

Table 4: List of acronyms

Name	Definition
XPS	X-ray photoelectron spectroscopy
PE	photoelectron
IMFP	inelastic mean free path
EMFP	elastic mean free path
EAL	effective attenuation length
LJ	liquid microjet
MD	molecular dynamics
CCD	charged couple devices
CEM	channel electron multiplier
SA/V	surface area to volume ratio $[\mathrm{m}^{-1}]$
MH	the Metropolis-Hastings algorithm
VMLM-B	variable metric limited memory with bounds algorithm
GT	ground truth
MAP	maximum a posteriori
SNR	signal-to-noise ratio
W_{10}, W_5	profile reconstruction case based on measurements on 10 and 5 attenuation lengths in the range [1.28, 5.50] nm
N_5	profile reconstruction case based on measurements 5 attenuation lengths in the range [1.62, 1.95] nm

References

Baek, S.-J., Park, A., Ahn, Y.-J. & Choo, J. (2015). *Analyst*, **140**(1), 250–257.

Berger, M. J. (1998). *National Institute of Standards and Technology (NIST)*, **8**, 3587.

Chambolle, A. & Pock, T. (2011). *Journal of Mathematical Imaging and Vision*, **40**(1), 120–145.

Dupuy, R., Richter, C., Winter, B., Meijer, G., Schlögl, R. & Bluhm, H. (2021). *The Journal of Chemical Physics*, **154**(6), 060901.

Emfietzoglou, D. & Nikjoo, H. (2007). *Radiation Research*, **167**(1), 110–120.

Fedoseenko, S., Vyalikh, D., Iossifov, I., Follath, R., Gorovikov, S., Püttner, R., Schmidt, J.-S., Molodtsov, S., Adamchuk, V., Gudat, W. *et al.* (2003). *Nuclear Instruments and Methods in Physics Research Section A: Accelerators, Spectrometers, Detectors and Associated Equipment*, **505**(3), 718–728.

Guilet, S., Bataillou, L., Kerivel, O. & Lazzari, R. (2022). *Journal of Electron Spectroscopy and Related Phenomena*, **258**, 147225.

Hastings, W. K. (1970). *Biometrika*, **57**, 97–109.

Kachel, T. (2016). *Journal of large-scale research facilities JLSRF*, **2**, A72–A72.

Kukk, E., Snell, G., Bozek, J. D., Cheng, W.-T. & Berrah, N. (2001). *Physical Review A*, **63**(6), 062702.

Kukk, E., Ueda, K., Hergenhahn, U., Liu, X.-J., Prümper, G., Yoshida, H., Tamenori, Y., Makochekanwa, C., Tanaka, T., Kitajima, M. *et al.* (2005). *Physical review letters*, **95**(13), 133001.

Leong, O., Gao, A. F., Sun, H. & Bouman, K. L., (2023). Ill-posed image reconstruction without an image prior.

Major, G. H., Fairley, N., Sherwood, P. M., Linford, M. R., Terry, J., Fernandez, V. & Artyushkova, K. (2020). *Journal of Vacuum Science & Technology A: Vacuum, Surfaces, and Films*, **38**(6), 061203.

Metropolis, N. & Ulam, S. (1949). *Journal of the American statistical association*, **44**(247), 335–341.

Nicholls, R. A., Long, F. & Murshudov, G. N. (2012). *Acta Crystallographica Section D: Biological Crystallography*, **68**(4), 404–417.

Olivieri, G., Goel, A., Kleibert, A. & Brown, M. A. (2015). *Journal of synchrotron radiation*, **22**(6), 1528–1530.

Olivieri, G., Parry, K. M., Powell, C. J., Tobias, D. J. & Brown, M. A. (2017). *Physical Chemistry Chemical Physics*, **19**(9), 6330–6333.

Ottosson, N., Faubel, M., Bradforth, S., Jungwirth, P. & Winter, B. (2010). *Journal of Electron Spectroscopy and Related Phenomena*, **177**(2-3), 60–70.

Ozon, M., Tumashevich, K. & Prisle, N. L. (2023). *Journal of synchrotron radiation*, **30**(4), 766–779.

Pereyra, M., Schniter, P., Chouzenoux, E., Pesquet, J.-C., Tourneret, J.-Y., Hero, A. O. & McLaughlin, S. (2015). *IEEE Journal of Selected Topics in Signal Processing*, **10**(2), 224–241.

Rudin, L., Osher, S. & Fatemi, E. (1992). *Physica D: Nonlinear Phenomena*, **60**(1), 259–268.

Siegbahn, H. & Siegbahn, K. (1973). *Journal of Electron Spectroscopy and Related Phenomena*, **2**(3), 319–325.

Stolzenburg, D., Ozon, M., Kulmala, M., Lehtinen, K. E., Lehtipalo, K. & Kangasluoma, J. (2022). *Journal of Aerosol Science*, **159**, 105862.

Thiébaut, E. (2002). In *Astronomical Data Analysis II*, vol. 4847, pp. 174–183. International Society for Optics and Photonics.

Thürmer, S., Seidel, R., Faubel, M., Eberhardt, W., Hemminger, J. C., Bradforth, S. E. & Winter, B. (2013). *Physical review letters*, **111**(17), 173005.

Twomey, S. (1963). *Journal of the ACM (JACM)*, **10**(1), 97–101.
